# The Content of Volatile Organic Compounds in *Calypogeia suecica* (Calypogeiaceae, Marchantiophyta) Confirms Genetic Differentiation of This Liverwort Species into Two Groups

**DOI:** 10.3390/molecules29174258

**Published:** 2024-09-08

**Authors:** Rafał Wawrzyniak, Wiesław Wasiak, Małgorzata Guzowska, Alina Bączkiewicz, Katarzyna Buczkowska

**Affiliations:** 1 Faculty of Chemistry, Adam Mickiewicz University in Poznań, Uniwersytetu Poznańskiego 8, 61-614 Poznań, Poland; wasiakw@amu.edu.pl (W.W.); malguz@amu.edu.pl (M.G.); 2 Faculty of Biology, Adam Mickiewicz University in Poznań, Uniwersytetu Poznańskiego 6, 61-614 Poznań, Poland; alinbacz@amu.edu.pl

**Keywords:** *Calypogeia suecica*, volatile organic compounds, liverworts, genetic groups, HS-SPME, GC-MS

## Abstract

*Calypogeia* is a genus of liverworts in the family Calypogeiaceae. The subject of this study was *Calypogeia suecica*. Samples of the liverwort *Calypogeia suecica* were collected from various places in southern Poland. A total of 25 samples were collected in 2021, and 25 samples were collected in 2022. Volatile organic compounds (VOCs) from liverworts were analyzed by gas chromatography–mass spectrometry (GC–MS). A total of 107 compounds were detected, of which 38 compounds were identified. The identified compounds were dominated by compounds from the sesquiterpene group (up to 34.77%) and sesquiterpenoids (up to 48.24%). The tested samples of *Calypogeia suecica* also contained compounds belonging the aromatic classification (up to 5.46%), aliphatic hydrocarbons (up to 1.66%), and small amounts of monoterpenes (up to 0.17%) and monoterpenoids (up to 0.30%). Due to the observed differences in the composition of VOCs, the tested plant material was divided into two groups, in accordance with genetic diversity.

## 1. Introduction

The genus *Calypogeia* is a representative of the leafy liverworts (from the suborder Jungemanniidae). The genus comprises about 90 species [1]. Most species of the genus occur in tropical and subtropical climates, while in the northern hemisphere, the diversity of species is limited [1,2,3,4]. There are nine species of this genus in Europe: *C. azurea*, *C. arguta*, *C. azorica*, *C. fissa*, *C. integristipula*, *C. muelleriana*, *C. neesiana*, *C. sphagnicola*, and *C. suecica* [2].

*Calypogeia suecica* is regarded as a boreal-montane species, and its presence has been reported in North America, Europe, and Asia. In Poland, it is widespread in the south, specifically in the mountains, and is very rare in the northeastern region. *Calypogeia suecica* is an obligate xylicole; it grows almost exclusively on moist decorticated logs in a later stage of decay, mainly in humid stream valleys in coniferous forests. It is the only *Calypogeia* species that grows on rotting logs [1]. *Calypogeia suecica* is a small plant; its shoots are up to 2.0 cm long and 1.8 mm wide (Figure S1). Characteristic features that distinguish *C. suecica* from other *Calypogeia* species are its almost orbicular leaves (composed of a single layer of small cells) with a truncate apex, its deeply divided underleaves, which are 2–3× wider than the stem with lateral angulation or teeth, and the colorless oil bodies present in all leaf and underleaf cells [5] (Figure S2). This is a dioecious species that is characterized by low morphological variability [5]. In Europe, two cytoforms of *C. suecica* have been reported, nine from Germany and Poland [6,7], and eighteen from Britain [8], which may support the hypothesis that an unrecognized species is present within *C. suecica*. Recently, molecular studies of the chloroplast genome have shown the genetic differentiation of *C. suecica* into two groups [9].

The presence of various volatile organic compounds (VOCs) in plants, animals, or microorganisms has led to the development of modern identification techniques such as high-performance liquid chromatography (HPLC) and gas chromatography–mass spectrometry (GC–MS), which have become popular identification tools due to their advantages of objectivity and accuracy [10]. These methods are used to identify species, varieties, and strains in a wide range of organisms, as well as the quality of various products and foods [11,12,13,14,15]. Liverworts are also rich in a wide range of biologically active compounds, such as terpenoids and aromatic compounds, which are synthesized and accumulated in oil bodies, cell structures characteristic of this group of plants [16,17,18]. Many of these compounds are specific only to liverworts [16,19].

Liverworts are small plants with a very simple morphological structure, and there are few good diagnostic features on the basis of which species can be recognized. Moreover, in liverworts. some species are genetically heterogeneous and, in fact, consist of morphologically cryptic or nearly cryptic taxa [20]. Therefore, the correct identification of liverwort species based solely on morphological characteristics has proven to be insufficient in many cases [21,22]. As previous studies have shown, volatile organic compounds (VOCs) can be helpful in identifying difficult-to-recognize closely related liverwort species belonging to the same genus, e.g., *Pellia*, *Riccardia*, *Pallavicinia*, *Mylia*, and *Porella* [16,23,24,25,26]. So far, the content of chemical compounds has been determined for about 10% of liverwort species [24], but only a few studies have combined chemotaxonomic analysis with molecular identification of the studied plants. As work on *Conocephalum conicum* [27] and *Aneura pinguis* [28] has shown, chemotaxonomic studies based on genetically identified material enable correct differentiation of cryptic species based on the detected VOCs. Additionally, chemotaxonomic studies of species belonging to the genus *Calypogeia* have shown that some species differ in their chemical composition [24,29,30]. So far, the analysis of chemical composition has been performed for only four European species of the genus *Calypogeia*: *C. azurea*, *C. muelleriana*, *C. fissa*, and *C. suecica* [31,32,33,34], which were carried out in the 1990s. However, at the time of these previous studies, there was no knowledge about the genetic diversity and the presence of hidden species in the genus *Calypogeia*, which were revealed by later genetic studies, e.g., within *C. muelleriana*, *C. sphagnicola*, *C. azurea* [35], and *C. suecica* [9]. To our knowledge, further analyses of *Calypogeia* species were conducted by Guzowska [36] and Wawrzyniak [37], and these studies were devoted to the seasonal variability of *C. azurea* and *C. integristipula*. However, so far, there have been no studies on the chemotaxonomic differentiation within *Calypogeia* species, combining chemical and genetic analyses.

The purpose of our study was to investigate whether the groups distinguished within *C. suecica* on the basis of genome analysis also differ in terms of composition and content of volatile chemical compounds.

## 2. Results and Discussion

### 2.1. Volatiles Present in Calypogeia suecica

Fifty *Calypogeia suecica* samples were tested for their content of volatile organic compounds (VOCs). Twenty-five samples were collected in 2021 (Table S1a–e) and twenty-five in 2022 (Table S2a–e). Due to the observed differences in the composition of volatile organic compounds, the samples were divided into two groups. The first group included 32 samples (CSU1-1–CSU1-32), while the second group included 18 samples (CSU2-1–CSU2-18).

One hundred and seven volatile compounds were detected in the biological material tested. Thirty-eight compounds were identified. The proportion of compounds identified in the first group ranged from 57.21% to 72.56%. However, in the case of the second group, the proportion of identified compounds ranged from 20.03% to 32.06%. Compounds that could not be identified were described using three characteristic ions: a molecular ion and two ions with the highest intensity. Based on the GC–MS analysis, it was found that the dominant groups of compounds in the tested plant material were sesquiterpenes and sesquiterpenoids.

Sesquiterpenoids were represented by bisabola-2,10-dien [1,9]oxide (**80**) and 4,5-dehydroviridiflorole (**73**). In the first group, these compounds constituted from 26.25 to 48.24% of the composition of the identified compounds. In the second group, identified sesquiterpenoids constituted only from 2.26% to 5.61% of the composition. However, in the second group, there were compounds with retention indexes of 1532 (**66**) and 1594 (**79**) in amounts ranging from 9.69% to 18.21% and 8.81% to 19.84%, respectively. MS spectra suggested that these compounds belonged to the sesquiterpenoid classification. It should be noted that the presence of these compounds was not detected in the first group.

In the case of the first group, sesquiterpenes were present at levels ranging from 17.78% to 34.77%. However, in the case of the second group, these compounds occurred at a rate of 14.39% to 24.88%. γ-curcumene (**53**) was the dominant sesquiterpene in the first group. It occurred at levels from 1.27% to 7.73%. In the case of the second group, the dominant sesquiterpene was γ-bisabolene (**60**). It was marked in the second group at a level from 4.17% to 8.74%. Bicyclogermacrene (**58**) is a sesquiterpene that was present at similar levels in groups one and two. In the case of the first group, its prevalence ranged from 1.07% to 6.48%. In the case of the second group, it ranged from 1.03% to 6.11%. A similar situation occurred with another sesquiterpene, anastreptene (**32**). In the case of the first group, its occurrence ranged from 4.60% to 9.53%. In the case of the second group, it ranged from 3.06% to 6.45%. Conversely, α-zingiberene (**57**) is a sesquiterpene that occurred in the first group at levels from 0.09% to 1.32%, and its presence was not detected in the second group. Other compounds belonging to the sesquiterpene group that were identified in the tested plant material included δ-elemene (**30**), α-funebrene (**33**), β-elemene (**34**), 7-epi-sesquithujene (**35**), italicene (**36**), 9-aristolene (**37**), 1(10),8-aristoladiene (**38**), β-barbatene (**46**), α-curcumene (**55**) and β-sesquiphellandrene (**64**). Compounds belonging to the group of monoterpenes were also identified in the analyzed plant material, such as tricyclene (**6**), α-pinene (**7**), and β-pinene (**10**).

Monoterpenoids include compounds such as bornyl acetate (**26**) and isobornyl acetate (**27**). However, in the case of both monoterpenes and terpenoids, the detected amounts in the studied samples were small. Monoterpenes were present in amounts of 0.02% to 0.07% in the first group, and in the second group, 0.03% to 0.17%. Monoterpenoids occurred in the first group at levels from 0.00 to 0.08%, and in the second group from 0.00% to 0.30%.

**Figure 1:**
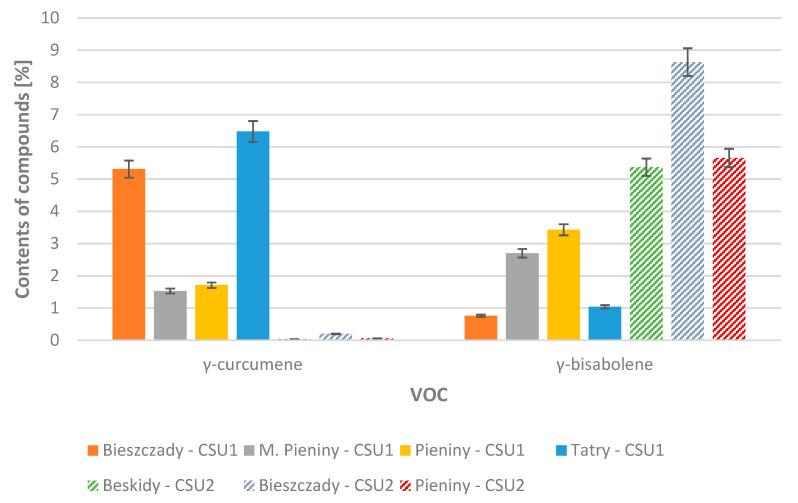
Average contents of selected dominant compounds in samples collected in years 2021–2022, taking into account the habitat (Beskidy, Bieszczady, M. Pieniny, Pieniny, Tatry) and group 1 (CSU1) or group 2 (CSU2).

**Figure 2:**
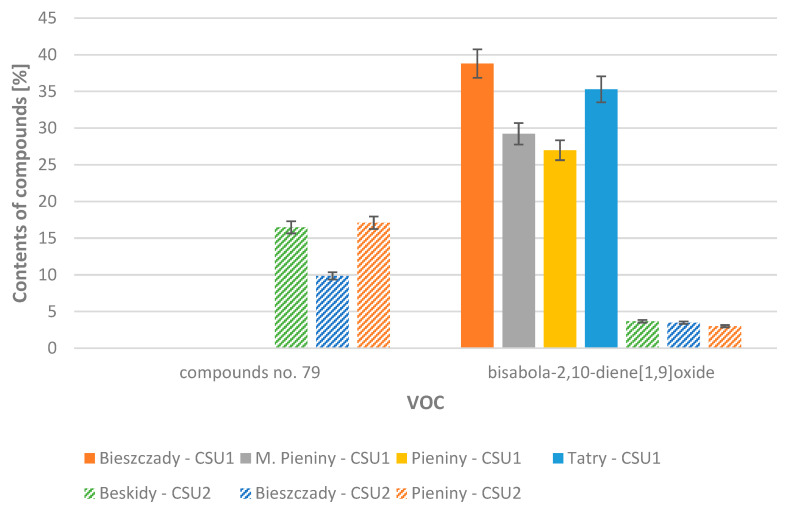
Average contents of selected dominant compounds in samples collected in year 2021–2022, taking into account the habitat (Beskidy, Bieszczady, M. Pieniny, Pieniny, Tatry) and group 1 (CSU1) or group 2 (CSU2).

Compounds belonging to the group of aliphatic compounds were also found in the cells of the liverwort samples tested: hexanal (**1**), 3-methylbutanoic acid (**2**), 2-methylbutanoic acid (**3**), 3-hexen-1-ol (**4**), 1-hexanol (**5**), hexanoic acid (**9**), 1-octen-3-ol (**11**), 3-octanone (**12**), 3-octanol (**13**), 2-ethylhexanoic acid (**16**); and aromatic compounds: benzenemethanol (**14**), benzeneacetaldehyde (**15**), benzeneethanol (**17**), phenoxyethanol (**23**), and 1-phenoxy-2-propanol (**24**). The content of aliphatic compounds in samples belonging to the first group ranged from 0.12% to 1.48%. In the second group, it ranged from 0.25% to 1.66%. Compounds classified as aromatic compounds constituted 0.88% to 5.46% of the composition in the first group, and in the second group 0.72% to 3.27% of the composition. Figure 1 and Figure 2 show a comparison of the average content of the dominant sesquiterpenes and sesquiterpenoids in the 2021 and 2022 samples, divided based on the location of the habitat from which the material was collected, and further into groups 1 and 2.

In the light of the information presented, it can be concluded that the analyzed biological material was clearly able to be divided into two groups in terms of the composition of volatile secondary metabolites. Some fluctuations in the composition of volatile compounds were also observed, resulting from the location of the sites from which the samples were collected.

Based on the collected results in Tables S1a–e and S2a–e, the mean % values of compound contents were calculated, along with the standard deviation for collection place within the group and for the group in total. Table 1 presents the results for group 1, and Table 2 presents the results for group 2. Table 2 also provides additional *t*-test values for groups.

### 2.2. Statistical Analysis of the Obtained Results

To investigate the variation in VOCs among two genetic groups of *C. suecica* (groups 1 and 2) revealed on the basis of chloroplast DNA [9], a set of 107 detected compounds were subjected to multivariate statistical analyses.

**Figure 3:**
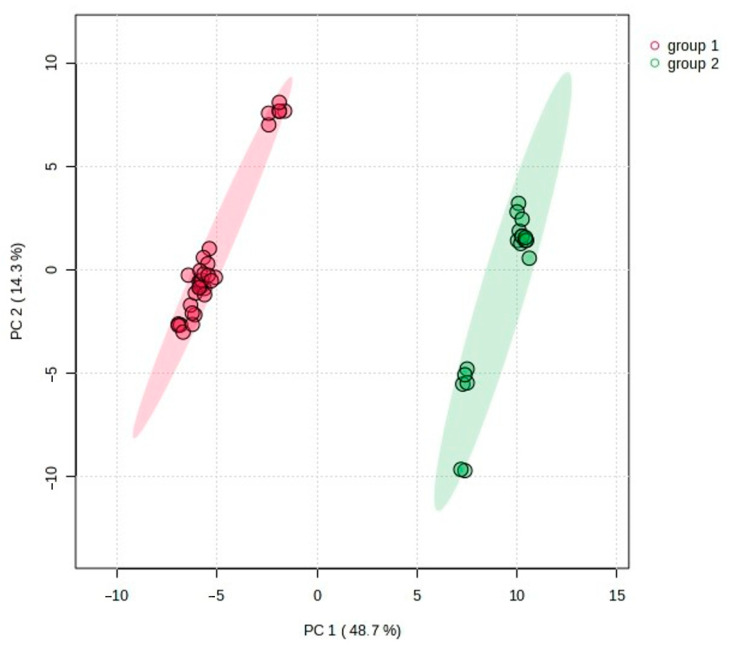
Two-dimensional PCA scatter plot based on all 107 detected compounds in the studied samples of *C. suecica*. The percentage of explained variance (R^2^X) is 48.7% for PC1 and 14.3% for PC2, and predictive ability (Q^2^) is 44.0% and 9.9%, respectively. Different colors indicate genetic group affiliation. Shaded ellipses indicate the 95% confidence regions.

**Figure 4:**
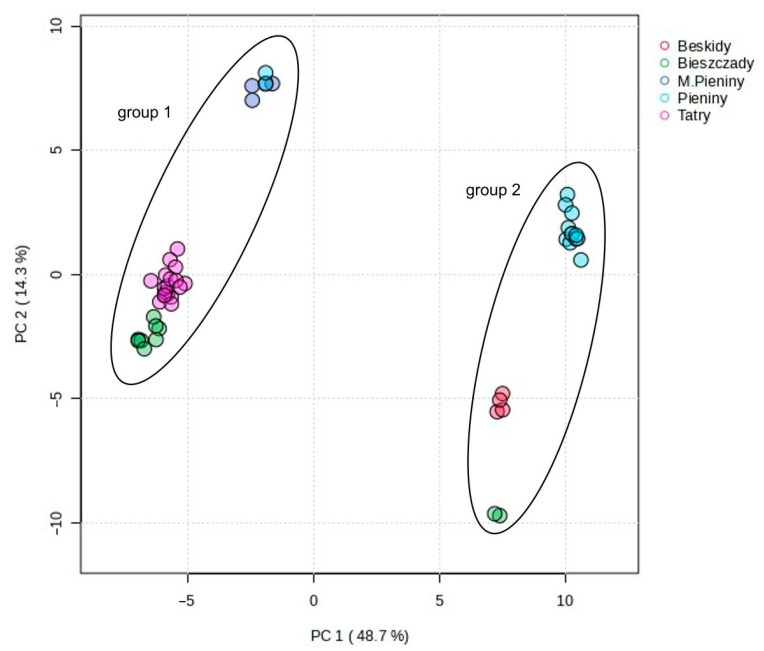
Two-dimensional PCA scatter plot based on all 107 detected compounds in the studied samples of *C. suecica* originating from different regions. The percentage of explained variance is 48.7% for PC1 and 14.3% for PC2. Different colors indicate the regions of origin.

**Figure 5:**
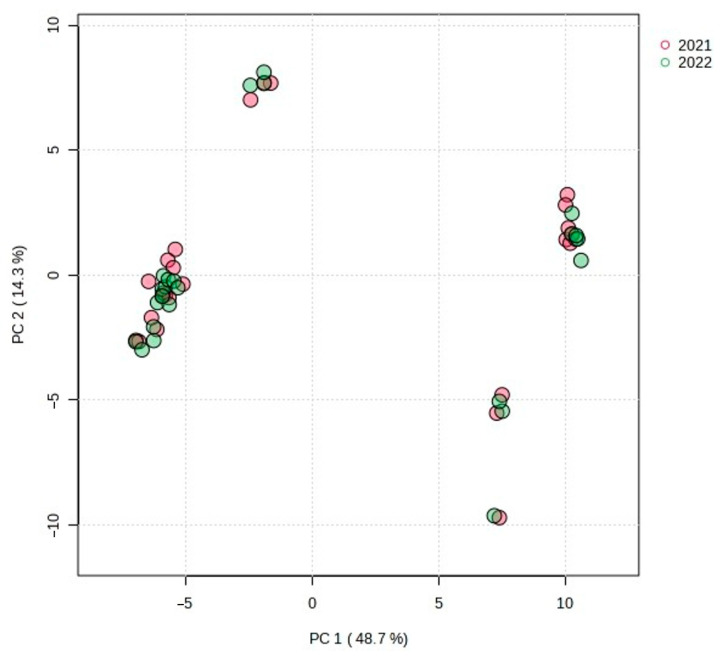
Two-dimensional PCA scatter plot based on all 107 detected compounds in samples of *C. suecica* collected in different years. The percentage of explained variance is 48.7% for PC1 and 14.3% for PC2. The year of sample collection is marked in different colors. Shaded ellipses indicate the 95% confidence regions.

First, a PCA was conducted, which is an unsupervised analysis used to reduce the dimensions of a large data set and to extract and visualize the hidden structure in the analyzed data. The explanatory and predictive abilities of the PCA model are evaluated on the basis of two parameters: R^2^X and Q^2^. The closer R^2^X and Q^2^ are to 1, the better the fitness of the model is. In the analysis of *C. suecica* samples, the model included five statistically significant components that explained 83.8% of the variation (R^2^X) and 75.7% of the predicted ability (Q^2^). However, the first two principal components, PC1 and PC2, explained as much as 63.0% of the total variance (R^2^X), with values of 48.7 and 14.3%, respectively. The PCA revealed a clear separation of the studied samples. The 2D scatterplot shows that the main differentiation between the studied samples, corresponding to their genetic group affiliation (group 1 and group 2), occurred along the PC1 axis, which explained 48.7% and predicted 44.0% of the total variation (Figure 3). The analysis of factor loadings indicates that the samples of group 1 and 2 differed primarily in the content of compounds **40**, **48**, **52**, **60**, **63**, **66**, **71**, **79**, **80**, **91**, and **106**, which made the largest contribution to the PC1 axis. Variables **40**, **48**, **60**, **63**, **66**, **71**, **79**, and **91** had high (>0.90) positive loading, whereas **52**, **80**, and **106** had high negative (>−0.90) loading (Figure S3). Therefore, samples belonging to group 1 located on the left (negative) side of the PCA diagram are characterized by a lower concentration (or lack) of the VOCs **40**, **48**, **60**, **63**, **66**, **71**, **79,** and **91**, as compared to group 2 located on the right (positive) side. In turn, samples from group 1 had a higher content of compounds **52**, **80**, and **106** than those from group 2. The separation of samples by genetic groups in PCA was confirmed by permutational multivariate analysis of variance (PERMANOVA, R^2^ = 0.75, *p* < 0.001). Smaller differentiation within both groups (1 and 2) was observed along the PC2 axis, explaining 14.3% and predicting 9.9% of the total variation. This variation is related to geographical diversity and reflects the fact that the samples tested came from different geographical regions of Poland (Figure 4). On the PC2 axis, the highest positive (>0.70) factor loadings had VOCs **11**, **15**, **16**, and **74**, while compound **45** had negative factor loadings (Figure S4). Analysis of samples from different regions of Poland revealed small differences in the geographical distributions of the groups studied. Group 2 occurred mainly in the Pieniny and rarely in the Beskid Sądecki and Bieszczady Mountains (only one location), while group 1 had a wider distribution; it occurred in the Tatry, Małe Pieniny, and Bieszczady Mountains, and less frequently in the Pieniny Mountains. It is worth emphasizing that at the site in the Łonny stream (in Pieniny), plants belonging to both groups grew together in one colony (samples CSU1-7 and CSU2-5) and had a composition of chemical compounds typical of the group (Tables S3 and S4). We did not observe visible differences between samples from a given region, nor between subsequent years of sample collection (Figure 5).

**Figure 6:**
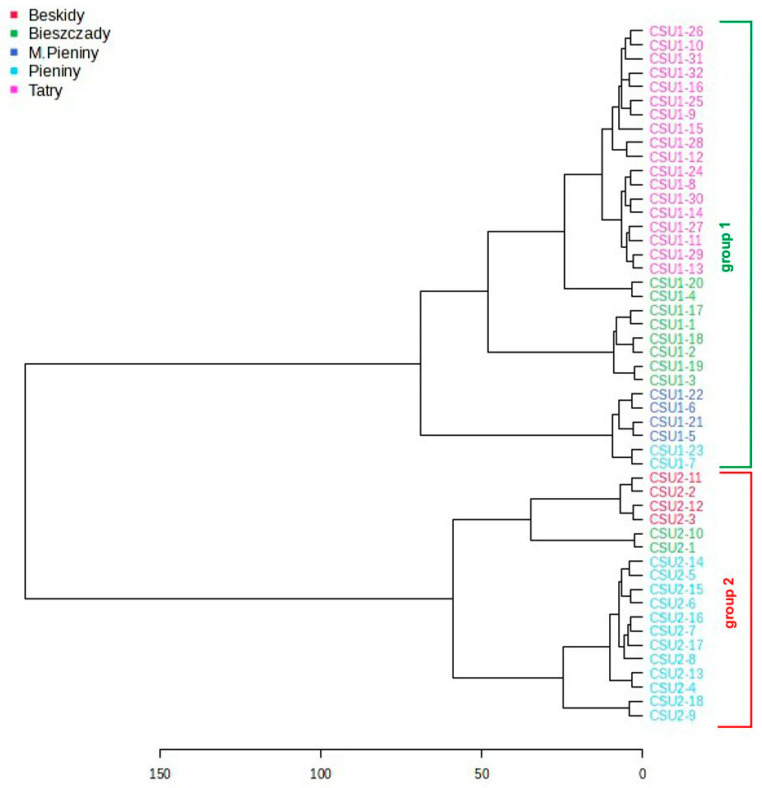
Dendrogram of examined samples of *C. suecica* samples belonging to two genetic groups constructed on the basis of the Euclidean distance according to Ward’s linkage method using 107 detected VOCs in the studied *C. suecica* samples.

The same result is shown by hierarchical cluster analysis (HCA). The dendrogram plotted on the basis of the Euclidean distance for all 107 compounds using Ward′s method divided the studied samples into two clades that were consistent with their affiliation to the genetic groups. A large Euclidean distance between the two groups (>150) supports the significant difference in chemical composition between them. On the contrary, the Euclidean distances among samples in one group were about three times lower, suggesting that there are small variations between samples from a given group (1 or 2) that grow in different geographic regions (Figure 6).

**Figure 7:**
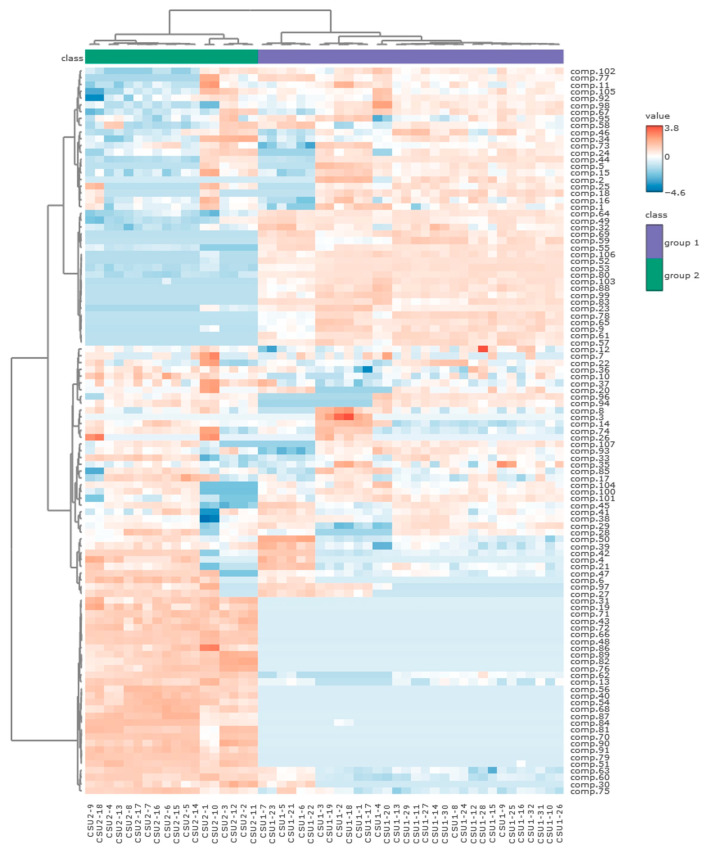
Clustering and heatmap analysis of the 107 chemical compounds detected in the studied *Calypogeia suecica* samples. The annotations bar shows clustering of the samples by group (class). Each cell was colored based on the level of the concentration of the chemical compound in the sample.

Similarly, the differentiation of the analyzed samples according to their genetic group was shown using a heatmap. The studied samples were separated into two main clusters correlated with their genetic classification, the first cluster including all 32 samples belonging to group 1, and the second containing 18 samples belonging to group 2. Heatmap analysis showed a high degree of correlation between the composition of VOCs in the analyzed samples of *C. suecica* and the genetic group. The compounds formed three groups, the content of which in the tested plants clearly changed depending on their genetic affiliation (Figure 7). Slight differences related to region can be seen in group 2, especially between the Bieszczady sample and the remaining samples, which had a lower content of compounds **38**, **41**, **45**, **100**, and **104** and a higher content of **1**, **11**, **15**, **16**, **25**, **46**, **77**, and **105** (Figure S5).

**Figure 8:**
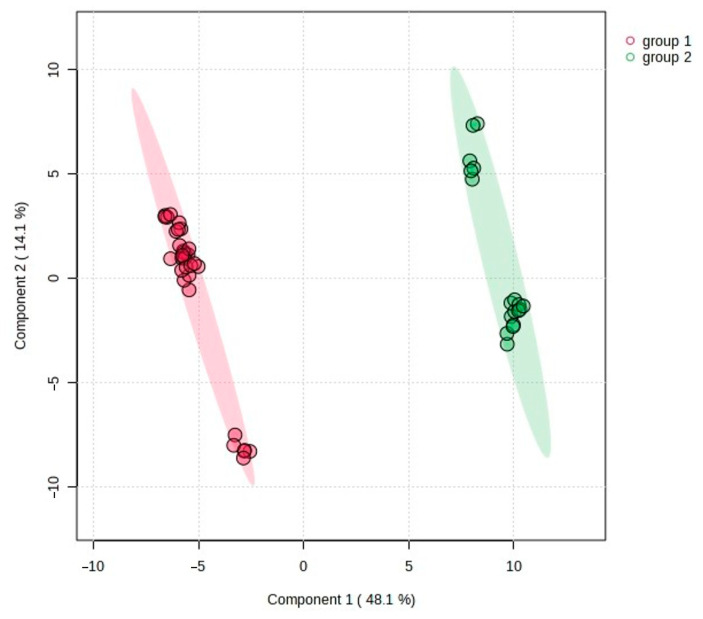
Two-dimensional PLS-DA separation of two genetic groups of *C. suecica* samples based on all 107 detected compounds. Shaded ellipses indicate the 95% confidence regions.

Results consistent with PCA, HCA, and heatmap analysis were also obtained in supervised PLS-DA analysis. The PLS-DA scatter plot showed a clear differentiation among the two genetic groups of *C. suecica*, indicating that the groups differed in their composition and content of the detected volatile compounds (Figure 8). The model identified significant separation between the two groups (R^2^X = 0.98, Q^2^ = 0.97, *p* < 0.001).

**Figure 9:**
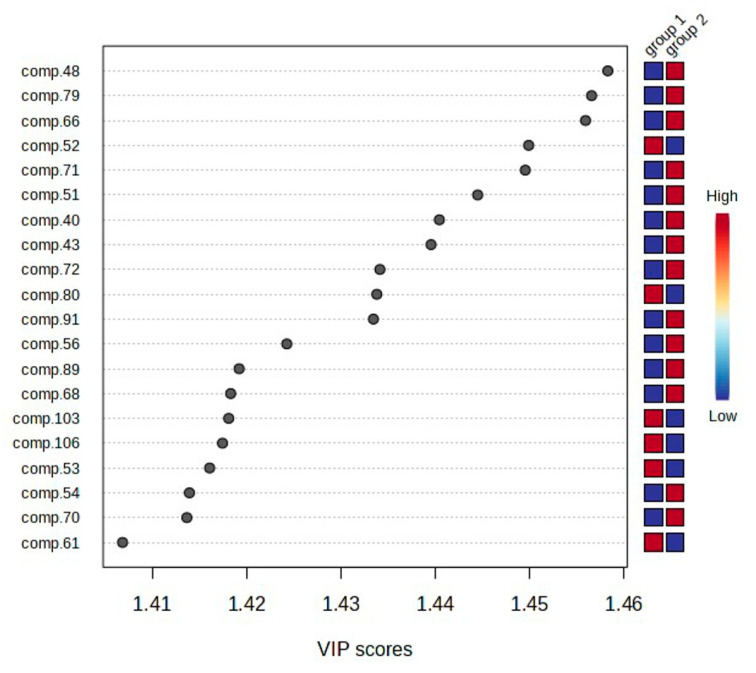
Variable importance in projection (VIP) identified by PLS-DA. The red and blue boxes on the right indicate whether the compound concentration is increased (red) or decreased (blue) in the samples of group 1 and 2 of *C. suecica*.

PLS-DA analysis makes it possible to determine the importance of variables in projection (VIP), which are key to the separation of the tested samples into groups. Figure 9 presents 20 key VOCs (VIP > 1.40) differentiating *C. suecica* samples belonging to different genetic groups. The higher the VIP result, the greater the contribution of the chemical compound to group separation. Among the chemical compounds indicated as the most important for distinguishing the studied groups, group 1, compared to group 2, was characterized by a reduced content of 14 compounds (**48**, **79**, **66**, **71**, **51**, **40**, **43**, **72**, **91**, **56**, **89**, **68**, **54**, and **70**) and an increased content of 6 compounds (**52**, **80**, **103**, **106**, **53**, and **61**).

The importance of the above VOCs for the identification of *C. suecica* groups was also confirmed by univariate analyses, such as fold change (FC), *t*-test, and volcano plot. According to the *t*-test (Table 2), the studied groups differed statistically significantly in the mean concentration of 78 chemical compounds, while the volcano plot, which combined the results of the fold change (FC) and *t*-test analyses into one single graph, showed significant differences for 64 VOCs: 38 sig. down, and 26 sig. up (Figures S6 and S7, Tables S7 and S8).

The observed differences in the composition of volatile organic compounds in the two genetic groups of *C. suecica* [9] are so distinct that they can serve as a marker enabling the reliable identification of plants that cannot be recognized based on morphological features. Both groups of *C. suecica* also differed clearly in their VOC composition from the other species of the genus *Calypogeia* occurring in Europe analyzed so far [32,33,34,36,37]. The results of our study confirmed the high content of bisabola-2,10-diene [1,9]oxide (**80**) in *C. suecica*, a compound detected for the first time in this species by Warmers et al. [34]. It should be emphasized that bisabola-2,10-diene [1,9]oxide (**80**) occurred in both genetic groups of *C. suecica*; however, the groups differed significantly in the average content of this compound. In group 1 it was 34.89%, while in group 2 it was several times lower, with an average of 3.20% (Table 1 and Table 2). Group 2 *C. suecica*, unlike group 1, contained two unidentified compounds (RI = 1532, RI = 1594) in amounts of 11.64 and 16.17%, respectively. Both *C. suecica* groups, similarly to *C. muelleriana* and *C. azurea*, were found to include compounds of the azulene type [32,36]. Azulenes are considered important chemical markers of *Calypogeia* species [29]. According to previous studies, *C. fissa* is dominated by acorane-type sesquiterpenes, which distinguishes this species from *C. suecica* [33]. In the case of *C. integristipula*, the dominant compounds are anastraptenes (15.61–25.26%) [37], which were also identified in *C. suecica*, but at a much lower level (4.69–7.13%). Bisabola-2,10-diene [1,9]oxide (**80**) is also present in *C. integristipula*, but in small amounts compared to *C. suecica*. The importance of the composition of volatile compounds as chemical markers has been proven in many studies of various liverwort species [16,23,24,25,26]. Our present studies have confirmed the great importance of using the HS-SPME/GC–MS method to profile volatile organic compounds (VOCs) in the case of closely related and morphologically indistinguishable liverwort species, as has been shown for the cryptic species *Conocephalum conicum* [27] and *Aneura pinguis* [28].

## 3. Materials and Methods

### 3.1. Plant Material

Fifty samples of the liverwort *C. suecica* collected in the years 2021–2022 from the natural environment in different regions of Poland were analyzed. The collected samples were approximately 5–7 cm in diameter. Detailed information on the samples, including the places of collection, geographic coordinates, and the dates of collection, are provided in Tables S3–S6.

The occurrence of *C. suecica* is limited only to moist rotting wood, mainly fir or spruce, which has an appropriate degree of decay (decorticated logs) [1,8]. For this reason, the species under study is not abundant in nature, and usually forms only small colonies. Due to the small number of sites and the specific nature of the habitat where this species occurs, we decided that all samples would be collected only in one growing season, in autumn. The autumn season was chosen because it ensures the optimal development phase and the best condition of the liverwort plants due to the prevailing weather conditions (higher humidity and lower temperatures). Unfortunately, this makes it impossible to carry out studies that illustrate changes in the composition of metabolites in different growing seasons, as was the case in [36]. The samples examined consisted of well-developed stems that were in a sterile state; that is, without reproductive structures. Research materials were collected from five geographical regions: the Bieszczady Mts, Beskidy Mts, Tatry Mts, Małe Pieniny Mts, and Pieniny Mts.

Five stems with a total weight of approximately 15 mg were taken from each sample. Only green plants that showed no signs of drying out and that were not affected by visible diseases were selected for further research. Before analysis, the samples were determined on the basis of morphological features, structure, and the distribution of the oil bodies in the leaves and under leaves [1,5,8]. The samples were classified into two groups detected by Ślipiko et al. [9] based on the chloroplast barcode marker *rbcL*. All samples classified to group 1 had the same sequence of *rbcL* as *C. suecica* acc. number MK294008, and those classified to group 2 had the same *rbcL* sequence as *C. suecica* acc. number MK294009, deposited in the GenBank by Ślipiko et al. [9].

### 3.2. HS-SPME Extraction

The VOCs from *Calypogeia suecica* were extracted using the headspace solid-phase microextraction technique (HS-SPME). Fused silica fibers coated with divinylbenzene/carboxen/polydimethylsiloxane (DVB/CAR/PDMS)(Merck KGaA, Darmstadt, Germany) were employed. The fibers, 2 cm in length and covered with a 50 µm DVB layer and a 30 µm CAR/PDMS layer, were conditioned for 1 h at 270 °C according to the supplier′s guidelines. A sample of 5 mg of clean and dried plant material was placed in a 1.7 mL vial, which was hermetically sealed with a Teflon/silicone septum and heated to 50 °C. The extraction of the compounds was conducted at 50 °C for 60 min. Desorption of analytes from the fibers was performed in the injection port of the gas chromatograph at 250 °C for 10 min. Sorption and desorption operations were performed using the TriPlus RSH autosampler (Thermo Scientific, Waltham, MA, USA).

### 3.3. GC-MS Analysis

The analysis of VOCs was performed using a previously described gas chromatography–mass spectrometry (GC–MS) method [36]. GC-MS analyses employing a Quadrex 007-5MS column (30 m, 0.25 mm, 0.25 μm)(Quadrex Corporation, Bethany, CT, USA) were conducted on a Trace 1310 (Thermo Scientific, Waltham, MA, USA). The ISQ QD mass detector (Thermo Scientific, Waltham, MA, USA) was operated at 70 eV in electron ionization (EI) mode within an *m*/*z* range of 30 to 550. Helium was used as the carrier gas at a flow rate of 1.0 mL/min. The oven temperature program was set to increase from 60 °C to 230 °C at a rate of 4 °C/min, followed by an isothermal hold at 230 °C for 40 min. The injector and transfer line temperatures were maintained at 250 °C. The samples were injected in splitless mode.

The identification of components was confirmed by comparing the mass spectral fragmentation patterns with those stored in the MS database (NIST 2011 [38], NIST Chemistry WebBook [39], Adams 4 Library [40], and Pherobase [41]) and with those reported in the literature. Furthermore, retention indices determined relative to a homologous series of n-alkanes (C7–C30)(Merck KGaA, Darmstadt, Germany) were compared with published data. Quantitative data for the components were obtained by integrating the total ion chromatogram (TIC) and calculating the relative percentage of peak areas. Each sample of *Calypogeia suecica* was analyzed in triplicate.

### 3.4. Statistical Analysis

Differences in the content of chemical compounds in individual samples of *C. suecica* were analyzed using multivariate statistical analyses. First, we performed uninspected principal component analysis (PCA) and hierarchical cluster analysis (HCA), which allowed the extraction and display of the hidden structures in the analyzed data set [42,43]. Then, using the classification of samples obtained in PCA and HCA, we performed PLS-DA analysis, which can be used for both classification and significant feature selection [44]. Using PLS-DA, we selected 20 of the most important variables in differentiating the detected groups based on the importance of variables in projection (VIP). To compare the concentration of detected compounds in individual samples belonging to both groups and collected in different geographic regions, the data were illustrated using a heat map, which allowed for the grouping of samples and variables simultaneously. Heatmapping is a common technique in biology that is useful for visualizing multivariate data [45]. To check the differences in the concentrations of the analyzed compounds between the two detected groups, single factor analyses such as fold change (FC), *t*-test, and volcano plot were used. Principal component analysis (PCA), partial least squares discriminant analysis (PLS-DA), and heatmap analysis were performed using the MetaboAnalyst 6.0 web portal (https://www.metaboanalyst.ca, accessed on 7 July 2024) [46]. STATISTICA 13.3 (StatSoft, Kraków, Poland) was used to perform the remaining analyses. Before statistical analyses, the obtained chromatographic data were subjected to log transformation (base 10) and auto-scaling (mean-centered and divided by the standard deviation of each variable).

## 4. Conclusions

GC–MS analysis of the volatile organic compounds present in the liverwort *Calypogeia suecica* cells revealed the presence of 107 compounds. Based on MS spectra, 38 compounds were identified. Among the identified metabolites, compounds from the sesquiterpene and sesquiterpenoid groups dominated. Aliphatic and aromatic compounds constituted only a maximum of 1.66 and 5.46% of the metabolite composition, respectively. The content of compounds belonging to the monoterpene and monoterpenoid groups in the studied liverwort species did not exceed 0.17% and 0.30% of the VOC composition, respectively. The observed differences in the composition of volatile organic compounds were significant enough to allow the separation of two groups within *Calypogeia suecica*. In group 1 of *Calypogeia suecica*, the dominant compound was bisabola-2,10-diene [1,9]oxide (up to 47.87%), which belongs to the sesquiterpenoid group. In group 2, two unidentified compounds dominated, with retention indices of 1532 (up to 18.21%) and 1594 (up to 19.84%). Based on MS spectra, it can be concluded that these compounds are sesquiterpenoids. Multivariate statistical analyses (PCA and PLS-DA) and heatmaps indicated that the differences detected in the composition and concentration of VOC in the examined *Calypogeia suecica* samples are consistent with genetic diversity. The analysis also showed that the composition of metabolites is slightly influenced by the habitat in which the liverwort *Calypogeia suecica* grows.

## Figures and Tables

**Table 1:** Mean % and standard deviation of volatile compounds detected in group 1 (CSU1) of *C. suecica* samples, divided based on group and collection place.

No.	Compounds	RI ^a^	Collection Place				Mean of Group 1
			Bieszczady Mts	Małe Pieniny Mts	Pieniny Mts	Tatry Mts	
1	hexanal	782	0.08 (0.03)	-	0.01 (0.01)	0.02 (0.01)	0.03 (0.03)
2	3-methylbutanoic acid	817	0.08 (0.05)	-	-	0.03 (0.01)	0.03 (0.04)
3	2-methylbutanoic acid	832	0.05 (0.05)	-	-	-	0.01 (0.03)
4	3-hexen-1-ol	858	-	0.02 (0.01)	0.04 (0.01)	-	0.01 (0.01)
5	1-hexanol	867	0.44 (0.27)	-	-	0.10 (0.05)	0.17 (0.21)
6	tricyclene	927	-	0.02 (0.01)	0.02 (0.01)	-	0.01 (0.01)
7	α-pinene	936	0.02 (0.01)	0.02 (0.01)	0.01 (0.00)	0.01 (0.01)	0.02 (0.01)
8	86[M+](50) 42(100) 86(38)	957	0.09 (0.05)	0.01 (0.00)	0.01 (0.00)	0.02 (0.01)	0.04 (0.04)
9	hexanoic acid	975	0.12 (0.05)	0.02 (0.01)	0.02 (0.01)	0.08 (0.03)	0.08 (0.05)
10	β-pinene	975	0.01 (0.01)	0.01 (0.01)	0.02 (0.01)	0.02 (0.01)	0.02 (0.01)
11	1-octen-3-ol	979	0.13 (0.07)	0.06 (0.01)	0.04 (0.01)	0.06 (0.02)	0.08 (0.05)
12	3-octanone	985	0.07 (0.02)	0.07 (0.02)	0.02 (0.01)	0.13 (0.17)	0.10 (0.13)
13	3-octanol	994	-	0.01 (0.01)	0.01 (0.00)	0.01 (0.01)	0.01 (0.01)
14	benzenemethanol	1033	1.13 (0.34)	0.25 (0.06)	0.16 (0.01)	0.08 (0.04)	0.37 (0.48)
15	benzeneacetaldehyde	1043	0.32 (0.09)	0.03 (0.01)	0.09 (0.01)	0.17 (0.05)	0.18 (0.11)
16	2-ethylhexanoic acid	1108	0.11 (0.07)	0.02 (0.01)	0.03 (0.01)	0.09 (0.04)	0.08 (0.05)
17	benzeneethanol	1116	0.83 (0.31)	0.42 (0.09)	0.95 (0.06)	0.66 (0.35)	0.69 (0.33)
18	126[M+](11) 55(100) 98(84)	1154	0.07 (0.02)	-	-	0.07 (0.02)	0.06 (0.03)
19	122[M+](20) 91(100) 44(58)	1164	-	-	-	-	-
20	140[M+](4) 43(100) 57(60)	1200	-	0.04 (0.01)	0.03 (0.01)	0.02 (0.01)	0.02 (0.02)
21	128[M+](5) 44(100) 57(63)	1203	0.02 (0.01)	0.35 (0.05)	0.40 (0.04)	0.05 (0.04)	0.10 (0.13)
22	152[M+](92) 67(100) 109(98)	1217	0.16 (0.06)	0.17 (0.07)	0.20 (0.06)	0.35 (0.19)	0.27 (0.17)
23	phenoxyethanol	1223	1.52 (0.81)	0.40 (0.08)	0.20 (0.01)	0.33 (0.10)	0.63 (0.66)
24	1-phenoxy-2-propanol	1247	0.04 (0.03)	-	-	0.05 (0.02)	0.04 (0.03)
25	144[M+](38) 44(100) 129(72)	1258	0.02 (0.01)	-	-	0.03 (0.01)	0.02 (0.02)
26	bornyl acetate	1285	0.02 (0.02)	-	-	-	-
27	isobornyl acetate	1290	0.02 (0.02)	0.05 (0.02)	0.07 (0.01)	-	0.02 (0.02)
28	189(8) 121(100) 93(82)	1320	-	0.02 (0.01)	0.02 (0.01)	0.04 (0.02)	0.03 (0.02)
29	bicycloelemene	1341	0.21 (0.09)	0.99 (0.06)	1.52 (0.04)	1.14 (0.29)	0.91 (0.48)
30	δ-elemene	1343	-	0.04 (0.02)	0.02 (0.01)	-	0.01 (0.01)
31	204[M+](17) 81(100) 93(83)	1355	-	-	-	-	-
32	anastreptene	1370	6.45 (1.22)	8.26 (1.44)	8.68 (0.13)	7.02 (1.16)	7.13 (1.31)
33	α-funebrene	1385	0.40 (0.11)	0.23 (0.03)	0.29 (0.04)	0.53 (0.17)	0.45 (0.18)
34	β-elemene	1394	0.08 (0.02)	0.04 (0.02)	0.04 (0.02)	0.14 (0.06)	0.11 (0.06)
35	7-epi-sesquithujene	1408	0.22 (0.13)	0.10 (0.02)	0.08 (0.01)	0.18 (0.12)	0.17 (0.12)
36	italicene	1409	0.08 (0.03)	0.13 (0.03)	0.14 (0.02)	0.12 (0.05)	0.11 (0.04)
37	9-aristolene	1423	0.05 (0.02)	0.06 (0.02)	0.21 (0.03)	0.07 (0.02)	0.07 (0.04)
38	1(10),8-aristoladiene	1429	3.64 (1.34)	5.83 (0.31)	5.39 (0.11)	4.44 (1.47)	4.48 (1.44)
39	204[M+](6) 107(100) 79(48)	1432	0.05 (0.03)	0.26 (0.07)	0.40 (0.02)	0.06 (0.02)	0.10 (0.11)
40	202[M+](4) 91(100) 185(89)	1434	-	-	-	-	-
41	204[M+](24) 91(100) 105(92)	1436	0.11 (0.01)	0.25 (0.04)	0.32 (0.04)	0.17 (0.06)	0.18 (0.07)
42	204[M+](18) 107(100) 161(88)	1438	0.08 (0.03)	8.07 (0.87)	8.29 (0.18)	0.17 (0.05)	1.64 (3.19)
43	204[M+](9) 119(100) 91(64)	1439	-	-	-	-	-
44	202[M+](30) 131(100) 159(62)	1440	6.51 (0.77)	0.63 (0.10)	0.70 (0.01)	7.04 (0.88)	5.71 (2.59)
45	202[M+](24) 69(100) 41(87)	1443	0.21 (0.08)	1.23 (0.32)	1.55 (0.09)	0.49 (0.24)	0.58 (0.45)
46	β-barbatene	1445	0.38 (0.15)	0.16 (0.03)	0.10 (0.01)	0.70 (0.37)	0.51 (0.37)
47	202[M+](19) 91(100) 41(85)	1450	0.19 (0.07)	0.44 (0.09)	0.60 (0.06)	0.24 (0.12)	0.28 (0.15)
48	202[M+](23) 91(100) 159(93)	1452	-	-	-	-	-
49	202[M+](23) 159(100) 131(74)	1455	2.12 (0.29)	2.97 (0.84)	3.16 (0.08)	2.13 (0.54)	2.30 (0.62)
50	204[M+](7) 159(100) 91(97)	1457	0.12 (0.03)	7.26 (1.39)	6.52 (0.08)	0.26 (0.15)	1.49 (2.74)
51	218[M+](26) 148(100) 133(75)	1466	-	-	-	-	-
52	204[M+](36) 119(100)93(61)	1469	5.21 (1.06)	2.87 (0.39)	6.40 (0.04)	6.41 (1.00)	5.66 (1.50)
53	γ-curcumene	1475	5.31 (0.58)	1.53 (0.27)	1.71 (0.04)	6.48 (1.09)	5.27 (2.05)
54	218[M+](12) 105(100) 91(92)	1476	-	-	-	-	-
55	α-curcumene	1477	1.93 (0.97)	4.75 (0.39)	1.50 (0.06)	2.69 (0.67)	2.68 (1.12)
56	218[M+](25) 105(100) 91(95)	1477	-	-	-	-	-
57	α-zingiberene	1479	0.58 (0.46)	0.34 (0.06)	0.33 (0.05)	0.68 (0.31)	0.59 (0.34)
58	bicyclogermacrene	1481	2.05 (0.74)	5.95 (0.52)	3.77 (0.08)	3.35 (0.67)	3.38 (1.31)
59	202[M+](29) 91(100) 133(92)	1500	0.28 (0.05)	2.20 (0.15)	1.31 (0.09)	2.82 (1.49)	2.01 (1.55)
60	γ-bisabolene	1505	0.76 (0.21)	2.70 (0.61)	3.43 (0.05)	1.04 (0.31)	1.33 (0.87)
61	202[M+](32) 133(100) 105(69)	1510	1.43 (0.36)	0.50 (0.04)	1.67 (0.11)	1.85 (0.46)	1.57 (0.59)
62	218[M+](29) 91(100) 93(97)	1513	-	0.01 (0.02)	-	0.08 (0.11)	0.05 (0.09)
63	218[M+](11) 132(100) 105(93)	1519	0.20 (0.04)	0.67 (0.11)	1.24 (0.08)	0.27 (0.13)	0.36 (0.29)
64	β-sesquiphellandrene	1524	1.26 (0.29)	1.67 (0.40)	1.60 (0.13)	1.46 (0.24)	1.45 (0.29)
65	218[M+](3) 159(100) 131(76)	1529	2.04 (1.21)	0.34 (0.07)	0.28 (0.05)	1.35 (0.37)	1.33 (0.86)
66	218[M+](24) 148(100) 133(63)	1532	-	-	-	-	-
67	220[M+](8) 85(100) 135(89)	1545	0.44 (0.18)	0.09 (0.04)	0.11 (0.03)	0.36 (0.10)	0.33 (0.16)
68	218[M+](4) 135(100) 107(42)	1548	-	-	-	-	-
69	202[M+](85) 131(100) 91(81)	1551	0.16 (0.09)	0.38 (0.06)	0.28 (0.08)	0.24 (0.10)	0.24 (0.11)
70	218[M+](6) 91(100) 157(90)	1554	-	-	-	-	-
71	218[M+](25) 145(100) 147(97)	1561	-	-	-	-	-
72	218[M+](4) 93(100) 43(75)	1568	-	-	-	-	-
73	4,5-dehydroviridiflorol	1572	1.57 (0.77)	0.09 (0.08)	0.03 (0.01)	0.57 (0.19)	0.73 (0.66)
74	222[M+](3) 43(100) 81(53)	1578	0.95 (0.60)	0.18 (0.08)	0.41 (0.05)	0.15 (0.07)	0.37 (0.45)
75	218[M+](6) 43(100) 93(57)	1579	0.25 (0.14)	0.78 (0.48)	0.14 (0.03)	0.16 (0.06)	0.26 (0.27)
76	218[M+](7) 43(100) 91(67)	1581	-	-	-	-	-
77	220[M+](14) 79(100) 93(93)	1584	0.30 (0.18)	0.81 (0.24)	0.79 (0.05)	0.19 (0.08)	0.33 (0.27)
78	220[M+](1) 94(100) 79(43)	1589	1.01 (0.56)	0.19 (0.03)	0.20 (0.03)	1.48 (0.44)	1.12 (0.65)
79	218[M+](25) 145(100) 147(87)	1594	-	-	-	-	-
80	bisabola-2,10-diene [1,9]oxide	1596	38.81 (5.47)	29.23 (3.15)	26.99 (0.47)	35.29 (4.35)	34.89 (5.51)
81	218[M+](1) 94(100) 79(52)	1605	-	-	-	-	-
82	218[M+](26) 145(100) 43(92)	1613	-	-	-	-	-
83	220[M+](2) 94(100) 79(39)	1625	2.97 (1.01)	2.43 (0.17)	2.33 (0.01)	1.84 (0.83)	2.23 (0.92)
84	218[M+](5) 145(100) 160(48)	1641	-	-	-	-	-
85	218[M+](18) 105(100) 120(83)	1646	0.32 (0.13)	0.03 (0.01)	0.03 (0.01)	0.09 (0.04)	0.14 (0.13)
86	218[M+](5) 135(100) 107(52)	1651	-	-	-	-	-
87	220[M+](2) 91(100) 43(91)	1658	-	-	-	-	-
88	220[M+](8) 159(100) 91(81)	1668	0.35 (0.17)	0.11 (0.05)	0.10 (0.04)	0.22 (0.07)	0.23 (0.13)
89	220[M+](5) 161(100) 91(69)	1670	-	-	-	-	-
90	218[M+](38) 145(100) 91(51)	1677	-	-	-	-	-
91	218[M+](2) 179(100) 161(92)	1686	-	-	-	-	-
92	218[M+](9) 105(100) 119(59)	1689	0.23 (0.18)	0.14 (0.05)	0.10 (0.03)	0.19 (0.02)	0.19 (0.10)
93	218[M+](22) 83(100) 94(92)	1699	0.21 (0.08)	0.03 (0.01)	0.04 (0.01)	0.18 (0.04)	0.16 (0.08)
94	218[M+](28) 135(100) 91(77)	1701	0.03 (0.05)	-	-	0.05 (0.01)	0.04 (0.03)
95	218[M+](20) 91(100) 133(98)	1706	0.12 (0.06)	0.10 (0.03)	0.10 (0.01)	0.07 (0.03)	0.09 (0.04)
96	220[M+](4) 110(100) 95(62)	1708	0.08 (0.14)	-	-	0.12 (0.02)	0.08 (0.08)
97	218[M+](11) 123(100) 95(62)	1712	0.06 (0.03)	0.11 (0.03)	0.13 (0.01)	-	0.04 (0.05)
98	220[M+](18) 83(100) 125(79)	1722	0.48 (0.63)	0.05 (0.01)	0.04 (0.01)	0.13 (0.04)	0.20 (0.34)
99	218[M+](2) 120(100) 83(34)	1729	0.14 (0.03)	0.03 (0.02)	0.03 (0.01)	0.06 (0.02)	0.07 (0.05)
100	218[M+](1) 121(100) 165(59)	1739	0.10 (0.02)	0.03 (0.01)	0.06 (0.02)	0.06 (0.02)	0.06 (0.03)
101	218[M+](2) 82(100) 41(50)	1744	0.09 (0.04)	0.05 (0.04)	0.06 (0.01)	0.07 (0.02)	0.07 (0.03)
102	218[M+](3) 82(100) 41(49)	1754	0.12 (0.07)	0.08 (0.02)	0.10 (0.01)	0.09 (0.03)	0.10 (0.04)
103	220[M+](9) 137(100) 135(78)	1759	0.17 (0.08)	0.05 (0.02)	0.05 (0.02)	0.12 (0.03)	0.12 (0.06)
104	218[M+](29) 136(100) 121(81)	1762	0.17 (0.11)	0.09 (0.05)	0.09 (0.02)	0.08 (0.03)	0.11 (0.07)
105	218[M+](1) 183(100) 198(61)	1795	0.16 (0.15)	0.09 (0.03)	0.08 (0.01)	0.09 (0.05)	0.11 (0.08)
106	218[M+](17) 82(100) 109(83)	1800	0.15 (0.04)	0.06 (0.03)	0.07 (0.03)	0.12 (0.03)	0.11 (0.04)
107	221[M+](1) 82(100) 67(39)	1808	0.10 (0.03)	-	-	0.09 (0.03)	0.07 (0.05)
	Total		96.84 (23.84)	97.70 (13.98)	95.96 (3.07)	97.91 (20.59)	97.47 (37.97)
	% Identified		68.77 (14.85)	63.50 (7.83)	57.52 (1.49)	67.74 (12.46)	66.81 (18.49)
	Including:						
	Aliphatics		1.08 (0.61)	0.20 (0.08)	0.17 (0.06)	0.52 (0.34)	0.60 (0.62)
	Aromatics		3.84 (1.58)	1.10 (0.24)	1.40 (0.09)	1.29 (0.56)	1.91 (1.60)
	Monoterpene hydrocarbons		0.03 (0.02)	0.05 (0.03)	0.05 (0.02)	0.03 (0.02)	0.04 (0.03)
	Monoterpenoide hydrocarbons		0.04 (0.04)	0.05 (0.02)	0.07 (0.01)	-	0.02 (0.03)
	Sesquiterpene hydrocarbons		23.40 (6.36)	32.78 (4.23)	28.81 (0.83)	30.04 (7.00)	28.63 (10.04)
	Sesquiterpenoide hydrocarbons		40.38 (6.24)	29.32 (3.23)	27.02 (0.48)	35.86 (4.54)	35.62 (6.17)

- less than 0.01%. ^a^ Retention index on Quadrex 007-5MS column. ( ) standard deviation.

**Table 2:** Mean % and standard deviation of volatile compounds detected in group 2 (CSU2) of *C. suecica* samples divided by group and collection place, and *t*-test values for groups 1 and 2.

No.	Compounds	RI ^a^	Collection Place			Mean of Group 2	*t*-Test for Groups *p* Value
			Bieszczady Mts	Beskid Sądecki Mts	Pieniny Mts		
1	hexanal	782	0.09 (0.01)	0.02 (0.01)	0.02 (0.01)	0.02 (0.02)	0.308
2	3-methylbutanoic acid	817	-	-	-	-	0.000
3	2-methylbutanoic acid	832	-	-	-	-	0.115
4	3-hexen-1-ol	858	-	-	0.02 (0.02)	0.02 (0.02)	0.015
5	1-hexanol	867	0.29 (0.08)	0.08 (0.03)	-	0.05 (0.09)	0.030
6	tricyclene	927	0.03 (0.01)	-	0.06 (0.02)	0.04 (0.03)	0.000
7	α-pinene	936	0.06 (0.01)	0.01 (0.01)	0.02 (0.01)	0.02 (0.02)	0.302
8	86[M+](50) 42(100) 86(38)	957	0.06 (0.01)	0.02 (0.01)	0.03 (0.01)	0.03 (0.02)	0.451
9	hexanoic acid	975	0.02 (0.01)	-	-	-	0.000
10	β-pinene	975	0.05 (0.01)	0.02 (0.01)	0.05 (0.02)	0.04 (0.02)	0.000
11	1-octen-3-ol	979	0.37 (0.04)	0.05 (0.02)	0.02 (0.01)	0.07 (0.11)	0.677
12	3-octanone	985	0.14 (0.03)	0.08 (0.01)	0.08 (0.02)	0.08 (0.03)	0.566
13	3-octanol	994	0.49 (0.05)	0.12 (0.02)	0.14 (0.04)	0.17 (0.12)	0.000
14	benzenemethanol	1033	0.23 (0.03)	0.18 (0.03)	0.44 (0.26)	0.36 (0.24)	0.928
15	benzeneacetaldehyde	1043	0.84 (0.07)	0.21 (0.10)	0.06 (0.03)	0.18 (0.25)	0.925
16	2-ethylhexanoic acid	1108	0.24 (0.04)	0.06 (0.01)	0.05 (0.03)	0.07 (0.06)	0.721
17	benzeneethanol	1116	1.67 (0.18)	0.45 (0.11)	1.38 (0.66)	1.20 (0.68)	0.001
18	126[M+](11) 55(100) 98(84)	1154	0.06 (0.01)	-	0.01 (0.02)	0.01 (0.02)	0.000
19	122[M+](20) 91(100) 44(58)	1164	0.04 (0.01)	0.02 (0.01)	0.03 (0.02)	0.03 (0.02)	0.000
20	140[M+](4) 43(100) 57(60)	1200	0.16 (0.03)	-	0.01 (0.01)	0.03 (0.05)	0.241
21	128[M+](5) 44(100) 57(63)	1203	-	-	0.14 (0.09)	0.10 (0.10)	0.924
22	152[M+](92) 67(100) 109(98)	1217	1.21 (0.12)	0.07 (0.01)	0.23 (0.14)	0.30 (0.35)	0.675
23	phenoxyethanol	1223	0.19 (0.01)	0.03 (0.01)	-	0.03 (0.06)	0.000
24	1-phenoxy-2-propanol	1247	0.08 (0.01)	0.11 (0.01)	0.01 (0.01)	0.04 (0.04)	0.636
25	144[M+](38) 44(100) 129(72)	1258	0.14 (0.04)	0.02 (0.01)	0.03 (0.07)	0.04 (0.06)	0.205
26	bornyl acetate	1285	0.12 (0.02)	-	0.03 (0.07)	0.03 (0.07)	0.022
27	isobornyl acetate	1290	0.06 (0.01)	-	0.05 (0.01)	0.04 (0.03)	0.001
28	189(8) 121(100) 93(82)	1320	-	0.04 (0.02)	0.10 (0.07)	0.08 (0.07)	0.000
29	bicycloelemene	1341	0.17 (0.03)	0.85 (0.16)	0.87 (0.17)	0.78 (0.27)	0.313
30	δ-elemene	1343	0.03 (0.01)	0.14 (0.05)	0.04 (0.02)	0.06 (0.05)	0.000
31	204[M+](17) 81(100) 93(83)	1355	0.31 (0.05)	0.29 (0.12)	0.18 (0.16)	0.21 (0.15)	0.000
32	anastreptene	1370	6.33 (0.17)	5.29 (0.62)	4.21 (0.52)	4.69 (0.90)	0.000
33	α-funebrene	1385	0.10 (0.03)	0.22 (0.07)	0.81 (0.13)	0.60 (0.32)	0.036
34	β-elemene	1394	0.49 (0.02)	0.65 (0.06)	0.04 (0.02)	0.23 (0.27)	0.021
35	7-epi-sesquithujene	1408	0.11 (0.01)	0.07 (0.02)	0.12 (0.03)	0.11 (0.03)	0.040
36	italicene	1409	0.16 (0.05)	0.14 (0.03)	0.14 (0.04)	0.14 (0.03)	0.004
37	9-aristolene	1423	0.31 (0.02)	0.14 (0.02)	0.10 (0.04)	0.13 (0.07)	0.001
38	1(10),8-aristoladiene	1429	0.10 (0.02)	3.15 (0.73)	3.57 (0.63)	3.09 (1.25)	0.001
39	204[M+](6) 107(100) 79(48)	1432	0.11 (0.03)	0.14 (0.06)	0.14 (0.04)	0.14 (0.04)	0.049
40	202[M+](4) 91(100) 185(89)	1434	0.12 (0.01)	0.11 (0.03)	0.15 (0.05)	0.14 (0.04)	0.000
41	204[M+](24) 91(100) 105(92)	1436	-	0.22 (0.08)	0.19 (0.12)	0.17 (0.12)	0.951
42	204[M+](18) 107(100) 161(88)	1438	-	0.26 (0.07)	5.04 (0.37)	3.41 (2.38)	0.045
43	204[M+](9) 119(100) 91(64)	1439	0.10 (0.03)	0.17 (0.06)	0.12 (0.04)	0.12 (0.05)	0.000
44	202[M+](30) 131(100) 159(62)	1440	9.02 (0.31)	5.84 (0.19)	0.53 (0.14)	2.65 (3.22)	0.001
45	202[M+](24) 69(100) 41(87)	1443	0.03 (0.01)	0.02 (0.01)	0.61 (0.22)	0.41 (0.33)	0.174
46	β-barbatene	1445	0.47 (0.06)	0.81 (0.11)	0.22 (0.16)	0.38 (0.28)	0.182
47	202[M+](19) 91(100) 41(85)	1450	2.31 (0.10)	-	1.35 (0.19)	1.16 (0.72)	0.000
48	202[M+](23) 91(100) 159(93)	1452	1.99 (0.06)	1.53 (0.11)	1.37 (0.09)	1.47 (0.22)	0.000
49	202[M+](23) 159(100) 131(74)	1455	0.30 (0.04)	1.73 (0.11)	0.26 (0.09)	0.59 (0.63)	0.000
50	204[M+](7) 159(100) 91(97)	1457	0.19 (0.02)	0.36 (0.15)	0.81 (0.43)	0.64 (0.43)	0.201
51	218[M+](26) 148(100) 133(75)	1466	0.20 (0.04)	0.87 (0.32)	0.70 (0.37)	0.68 (0.38)	0.000
52	204[M+](36) 119(100)93(61)	1469	0.05 (0.02)	-	-	0.01 (0.02)	0.000
53	γ-curcumene	1475	0.20 (0.01)	0.04 (0.01)	0.06 (0.02)	0.07 (0.05)	0.000
54	218[M+](12) 105(100) 91(92)	1476	1.42 (0.11)	0.53 (0.08)	1.60 (0.59)	1.34 (0.66)	0.000
55	α-curcumene	1477	-	-	0.14 (0.06)	0.09 (0.08)	0.000
56	218[M+](25) 105(100) 91(95)	1477	2.57 (0.17)	2.43 (0.63)	4.51 (1.59)	3.83 (1.64)	0.000
57	α-zingiberene	1479	-	-	-	-	0.000
58	bicyclogermacrene	1481	2.09 (0.18)	5.77 (0.23)	2.09 (1.84)	2.91 (2.16)	0.344
59	202[M+](29) 91(100) 133(92)	1500	-	-	-	-	0.000
60	γ-bisabolene	1505	8.63 (0.16)	5.37 (0.37)	5.66 (1.10)	5.93 (1.34)	0.000
61	202[M+](32) 133(100) 105(69)	1510	-	-	-	-	0.000
62	218[M+](29) 91(100) 93(97)	1513	1.54 (0.09)	1.99 (0.74)	1.31 (0.51)	1.49 (0.59)	0.000
63	218[M+](11) 132(100) 105(93)	1519	1.51 (0.04)	1.41 (0.14)	1.97 (0.30)	1.80 (0.36)	0.000
64	β-sesquiphellandrene	1524	0.04 (0.01)	0.33 (0.08)	0.21 (0.15)	0.22 (0.15)	0.000
65	218[M+](3) 159(100) 131(76)	1529	-	-	-	-	0.000
66	218[M+](24) 148(100) 133(63)	1532	17.33 (1.24)	11.53 (0.43)	10.73 (0.50)	11.64 (2.16)	0.000
67	220[M+](8) 85(100) 135(89)	1545	0.09 (0.03)	0.67 (0.08)	0.10 (0.05)	0.22 (0.25)	0.045
68	218[M+](4) 135(100) 107(42)	1548	0.10 (0.02)	0.16 (0.05)	0.26 (0.10)	0.22 (0.11)	0.000
69	202[M+](85) 131(100) 91(81)	1551	-	-	-	-	0.000
70	218[M+](6) 91(100) 157(90)	1554	0.12 (0.01)	2.35 (0.25)	2.27 (0.28)	2.05 (0.75)	0.000
71	218[M+](25) 145(100) 147(97)	1561	0.22 (0.05)	0.26 (0.06)	0.20 (0.05)	0.22 (0.05)	0.000
72	218[M+](4) 93(100) 43(75)	1568	0.60 (0.07)	0.54 (0.28)	0.63 (0.24)	0.61 (0.23)	0.000
73	4,5-dehydroviridiflorol	1572	0.96 (0.08)	1.17 (0.85)	0.52 (0.50)	0.72 (0.61)	0.940
74	222[M+](3) 43(100) 81(53)	1578	3.21 (0.09)	0.08 (0.03)	0.52 (0.14)	0.72 (0.93)	0.048
75	218[M+](6) 43(100) 93(57)	1579	0.48 (0.07)	0.67 (0.16)	1.25 (0.23)	1.04 (0.38)	0.000
76	218[M+](7) 43(100) 91(67)	1581	0.61 (0.02)	1.49 (0.11)	0.48 (0.25)	0.72 (0.47)	0.000
77	220[M+](14) 79(100) 93(93)	1584	6.41 (0.17)	0.14 (0.09)	-	0.74 (2.06)	0.263
78	220[M+](1) 94(100) 79(43)	1589	-	-	-	-	0.000
79	218[M+](25) 145(100) 147(87)	1594	9.87 (1.50)	16.49 (0.46)	17.11 (1.13)	16.17 (2.51)	0.000
80	bisabola-2,10-diene [1,9]oxide	1596	3.48 (0.22)	3.69 (1.57)	3.00 (1.06)	3.20 (1.12)	0.000
81	218[M+](1) 94(100) 79(52)	1605	0.11 (0.02)	5.26 (0.55)	3.67 (2.11)	3.63 (2.24)	0.000
82	218[M+](26) 145(100) 43(92)	1613	3.20 (0.09)	7.93 (1.04)	1.82 (0.68)	3.33 (2.66)	0.000
83	220[M+](2) 94(100) 79(39)	1625	-	-	-	-	0.000
84	218[M+](5) 145(100) 160(48)	1641	0.16 (0.02)	0.24 (0.04)	10.82 (1.13)	7.29 (5.23)	0.000
85	218[M+](18) 105(100) 120(83)	1646	0.04 (0.01)	0.12 (0.03)	0.17 (0.09)	0.14 (0.09)	0.838
86	218[M+](5) 135(100) 107(52)	1651	1.01 (0.15)	0.05 (0.02)	0.07 (0.03)	0.17 (0.31)	0.000
87	220[M+](2) 91(100) 43(91)	1658	0.14 (0.03)	0.14 (0.03)	0.91 (0.34)	0.65 (0.46)	0.000
88	220[M+](8) 159(100) 91(81)	1668	-	-	-	-	0.000
89	220[M+](5) 161(100) 91(69)	1670	0.44 (0.03)	1.00 (0.13)	0.36 (0.11)	0.51 (0.29)	0.000
90	218[M+](38) 145(100) 91(51)	1677	0.02 (0.01)	0.42 (0.19)	0.13 (0.06)	0.18 (0.16)	0.000
91	218[M+](2) 179(100) 161(92)	1686	0.03 (0.01)	0.06 (0.02)	0.11 (0.02)	0.09 (0.04)	0.000
92	218[M+](9) 105(100) 119(59)	1689	0.26 (0.04)	0.20 (0.12)	0.09 (0.06)	0.13 (0.10)	0.037
93	218[M+](22) 83(100) 94(92)	1699	0.07 (0.01)	0.20 (0.10)	0.15 (0.04)	0.15 (0.06)	0.709
94	218[M+](28) 135(100) 91(77)	1701	0.08 (0.01)	0.04 (0.01)	0.06 (0.02)	0.06 (0.02)	0.028
95	218[M+](20) 91(100) 133(98)	1706	0.07 (0.01)	0.11 (0.08)	0.05 (0.02)	0.07 (0.04)	0.048
96	220[M+](4) 110(100) 95(62)	1708	0.08 (0.02)	0.07 (0.02)	0.05 (0.03)	0.06 (0.03)	0.205
97	218[M+](11) 123(100) 95(62)	1712	0.68 (0.06)	-	0.07 (0.03)	0.12 (0.21)	0.037
98	220[M+](18) 83(100) 125(79)	1722	-	0.10 (0.05)	0.02 (0.01)	0.03 (0.04)	0.045
99	218[M+](2) 120(100) 83(34)	1729	-	-	-	-	0.000
100	218[M+](1) 121(100) 165(59)	1739	-	-	0.07 (0.04)	0.05 (0.05)	0.173
101	218[M+](2) 82(100) 41(50)	1744	0.01 (0.01)	-	0.03 (0.02)	0.02 (0.02)	0.000
102	218[M+](3) 82(100) 41(49)	1754	0.09 (0.01)	0.13 (0.02)	0.02 (0.01)	0.05 (0.05)	0.001
103	220[M+](9) 137(100) 135(78)	1759	-	-	-	-	0.000
104	218[M+](29) 136(100) 121(81)	1762	-	-	0.12 (0.02)	0.08 (0.06)	0.221
105	218[M+](1) 183(100) 198(61)	1795	0.47 (0.03)	0.22 (0.10)	0.02 (0.01)	0.11 (0.16)	0.820
106	218[M+](17) 82(100) 109(83)	1800	0.01 (0.01)	-	-	-	0.000
107	221[M+](1) 82(100) 67(39)	1808	0.02 (0.01)	-	0.09 (0.05)	0.06 (0.06)	0.414
	Total		98.11 (6.89)	98.01 (12.87)	98.10 (21.33)	98.03 (45.88)	
	% Identified		28.64 (1.70)	29.25 (5.36)	24.23 (7.71)	25.81 (10.91)	
	Including:						
	Aliphatics		1.64 (0.26)	0.41 (0.10)	0.33 (0.13)	0.48 (0.47)	
	Aromatics		3.01 (0.30)	0.98 (0.26)	1.89 (0.96)	1.81 (1.28)	
	Monoterpene hydrocarbons		0.14 (0.03)	0.03 (0.02)	0.13 (0.05)	0.10 (0.06)	
	Monoterpenoide hydrocarbons		0.18 (0.03)	-	0.08 (0.08)	0.08 (0.09)	
	Sesquiterpene hydrocarbons		19.23 (0.78)	22.97 (2.56)	18.28 (4.93)	19.42 (7.28)	
	Sesquiterpenoide hydrocarbons		4.44 (0.30)	4.86 (2.42)	3.52 (1.56)	3.92 (1.73)	

- less than 0.01%. ^a^ Retention index on Quadrex 007-5MS column. ( ) standard deviation.

## Data Availability

The data presented in this study are available on request from the corresponding author.

## Supplementary Materials

The following supporting information can be downloaded at: https://www.mdpi.com/article/10.3390/molecules29174258/s1, Figure S1. *Calypogeia suecica*, a colony showing several stems of the plant in its natural environment; Figure S2. Microscopic images of the (a) leaves, (b) under leaves, and (c) cells with oil bodies of *Calypogeia suecica*: 1-group 1, 2-group 2; Table S1a. Volatile compounds detected in the samples CSU1-1–CSU1-5; Table S1b. Volatile compounds detected in the samples CSU1-6–CSU1-10; Table S1c. Volatile compounds detected in the samples CSU1-11–CSU1-15; Table S1d. Volatile compounds detected in the samples CSU1-16–CSU2-4; Table S1e. Volatile compounds detected in the samples CSU2-5–CSU2-9; Table S2a. Volatile compounds detected in the samples CSU1-17–CSU1-21; Table S2b. Volatile compounds detected in the samples CSU1-22–CSU1-26; Table S2c. Volatile compounds detected in the samples CSU1-27–CSU1-31; Table S2d. Volatile compounds detected in the samples CSU1-32–CSU2-13; Table S2e. Volatile compounds detected in the samples CSU2-14–CSU2-18; Table S3. The *Calypogeia suecica* group 1 sampling data from the year 2021 used for studies; Table S4. The *Calypogeia suecica* group 2 sampling data from the year 2021 used for studies; Table S5. The *Calypogeia suecica* group 1 sampling data from the year 2022 used for studies; Table S6. The *Calypogeia suecica* group 2 sampling data from the year 2022 used for studies; Figure S3. Linear plot of the lodgings for the first principal component PC1; Figure S4. Linear plot of the lodgings for the second principal component PC2; Figure S5. Clustering and heatmap analysis of the 107 chemical compounds detected in the studied *Calypogeia suecica* samples. Annotations bar shows clustering of the samples by regions (class). Each cell was colored based on the level of the concentration of the chemical compound in the sample; Figure S6. Important features selected by *t*-tests with threshold 0.1. The red circles represent features above the threshold. Note the *p* values are transformed by −log10 so that the more significant features (with smaller *p* values) will be plotted higher on the graph [MetaboAnalyst 6.0]; Table S7. Top 50 features identified by *t*-test showing statistically significant differences between genetic groups (group 1 and 2) of *C. suecica*; Figure S7. Important features selected by volcano plot with fold change threshold (x) 2 and *t*-tests threshold (y) 0.1. The red circles represent features above the threshold. Note both fold changes and *p* values are log transformed. The further its position away from the (0,0), the more significant the feature is [MetaboAnalyst 6.0]; Table S8. Top 50 features identified by volcano plot distinguishing genetic groups (group 1 and 2) of *C. suecica*.
