# Supplementary material for: The Content of Volatile Organic Compounds in Calypogeia suecica (Calypogeiaceae, Marchantiophyta) Confirms Genetic Differentiation of This Liverwort Species into Two Groups
Source: Molecules. 2024 Sep 8;29(17):4258. doi: 10.3390/molecules29174258 (PMC11397266; doi:10.3390/molecules29174258)
Supplement: Supplementary file 1 [file molecules-29-04258-s001.zip › molecules-3155593-supplementary.pdf]

molecules

Supplementary Material

The content of volatile organic compounds in *Calypogeia suecica* (Calypogeiaceae, Marchantiophyta) confirms genetic differentiation of this liverwort species into two groups

Rafał Wawrzyniak [1,*], Wiesław Wasiak [1], Małgorzata Guzowska [1], Alina Bączkiewicz [2] and Katarzyna Buczkowska [2,*]

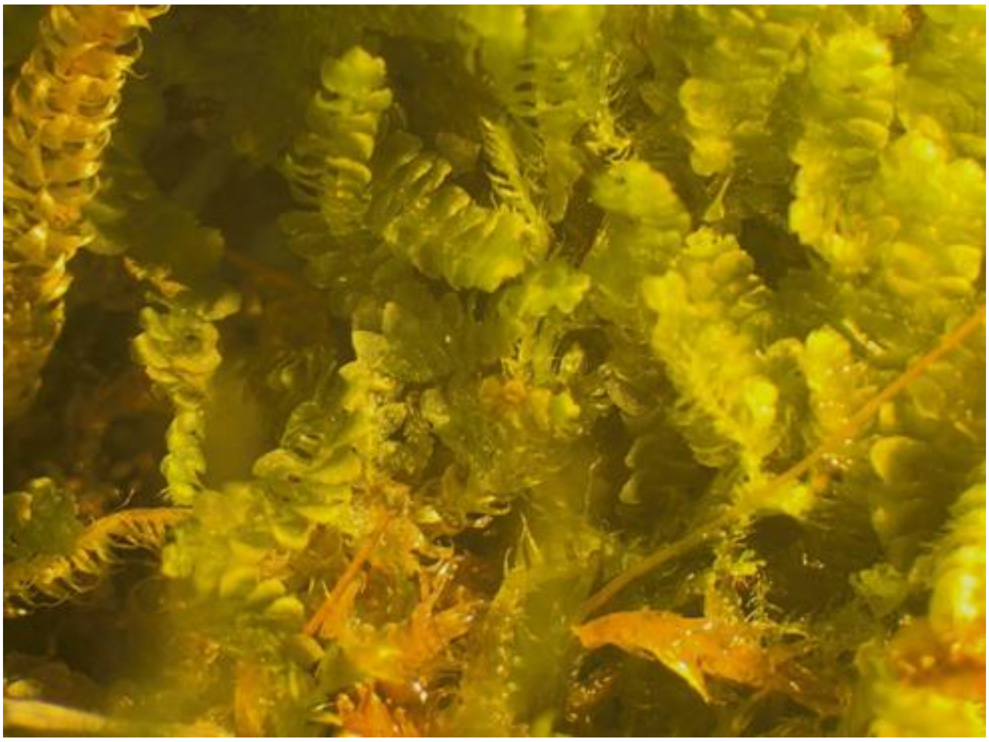

Figure S1. *Calypogeia suecica*, a colony showing several stems of the plant in its natural environment.

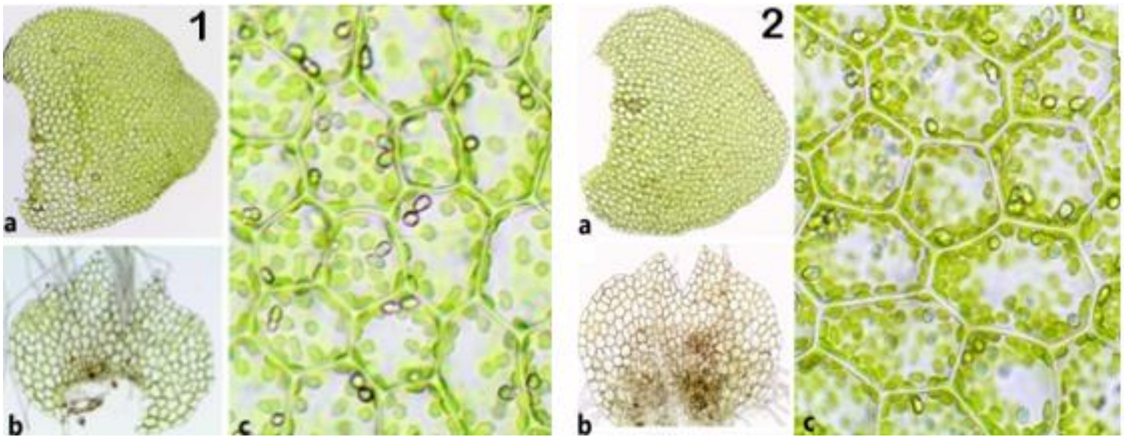

Figure S2. Microscopic images of the a) leaves, b) under leaves and c) cells with oil bodies of *Calypogeia suecica*: **1** – group 1, **2** – group 2.

Table S1a. Volatile compounds detected in the samples CSU1-1 – CSU1-5.

No.	Compounds	RI [a]	Code [b]				
			CSU1-1	CSU1-2	CSU1-3	CSU1-4	CSU1-5
1	hexanal	782	0.09 (0.01)	0.08 (0.00)	0.02 (0.01)	0.08 (0.02)	0.01 (0.00)
2	3-methylbutanoic acid	817	0.11 (0.01)	0.09 (0.01)	0.10 (0.02)	-	-
3	2-methylbutanoic acid	832	0.04 (0.01)	0.03 (0.01)	0.11 (0.01)	-	-
4	3-hexen-1-ol	858	-	-	-	-	0.02 (0.01)
5	1-hexanol	867	0.34 (0.03)	0.83 (0.04)	0.55 (0.03)	0.13 (0.03)	-
6	tricyclene	927	-	-	-	-	0.01 (0.00)
7	α-pinene	936	0.01 (0.00)	0.01 (0.00)	0.01 (0.00)	0.02 (0.00)	0.02 (0.01)
8	86[M+](50) 42(100) 86(38)	957	0.06 (0.01)	0.09 (0.02)	0.15 (0.01)	0.04 (0.01)	0.01 (0.00)
9	hexanoic acid	975	0.12 (0.02)	0.18 (0.01)	0.16 (0.02)	0.03 (0.01)	0.02 (0.01)
10	β-pinene	975	0.02 (0.00)	0.01 (0.00)	0.01 (0.00)	0.01 (0.00)	0.01 (0.00)
11	1-octen-3-ol	979	0.11 (0.01)	0.07 (0.01)	0.23 (0.01)	0.08 (0.01)	0.06 (0.02)
12	3-octanone	985	0.05 (0.01)	0.04 (0.01)	0.08 (0.01)	0.07 (0.01)	0.04 (0.01)
13	3-octanol	994	-	-	-	-	0.01 (0.00)
14	benzenemethanol	1033	1.58 (0.04)	1.01 (0.04)	1.31 (0.03)	0.70 (0.04)	0.32 (0.04)
15	benzeneacetaldehyde	1043	0.36 (0.02)	0.30 (0.02)	0.35 (0.02)	0.18 (0.02)	0.02 (0.00)
16	2-ethylhexanoic acid	1108	0.09 (0.01)	0.08 (0.02)	0.20 (0.01)	0.02 (0.00)	0.02 (0.00)
17	benzeneethanol	1116	0.98 (0.02)	0.81 (0.03)	1.10 (0.02)	0.35 (0.02)	0.47 (0.03)
18	126[M+](11) 55(100) 98(84)	1154	0.07 (0.01)	0.05 (0.01)	0.08 (0.01)	0.05 (0.01)	-
19	122[M+](20) 91(100) 44(58)	1164	-	-	-	-	-
20	140[M+](4) 43(100) 57(60)	1200	-	-	-	-	0.03 (0.01)
21	128[M+](5) 44(100) 57(63)	1203	0.02 (0.00)	0.01 (0.00)	0.01 (0.00)	0.03 (0.01)	0.29 (0.02)
22	152[M+](92) 67(100) 109(98)	1217	0.16 (0.01)	0.19 (0.01)	0.20 (0.01)	0.07 (0.01)	0.22 (0.02)
23	phenoxyethanol	1223	2.49 (0.04)	1.71 (0.04)	1.50 (0.04)	0.36 (0.04)	0.44 (0.06)
24	1-phenoxy-2-propanol	1247	0.05 (0.01)	0.06 (0.01)	0.02 (0.01)	-	-
25	144[M+](38) 44(100) 129(72)	1258	0.03 (0.01)	0.02 (0.00)	0.02 (0.01)	0.01 (0.00)	-
26	bornyl acetate	1285	0.01 (0.00)	0.03 (0.00)	0.01 (0.00)	-	-
27	isobornyl acetate	1290	0.01 (0.00)	0.04 (0.00)	0.02 (0.01)	-	0.03 (0.01)
28	189(8) 121(100) 93(82)	1320	-	-	-	-	0.01 (0.00)
29	bicycloelemene	1341	0.25 (0.02)	0.29 (0.02)	0.10 (0.02)	0.13 (0.02)	0.92 (0.04)
30	δ-elemene	1343	-	-	-	-	0.02 (0.00)
31	204[M+](17) 81(100) 93(83)	1355	-	-	-	-	-
32	anastreptene	1370	7.17 (0.03)	7.66 (0.04)	6.38 (0.04)	4.60 (0.03)	9.47 (0.03)
33	α-funebrene	1385	0.46 (0.02)	0.37 (0.03)	0.52 (0.02)	0.26 (0.02)	0.23 (0.02)
34	β-elemene	1394	0.07 (0.01)	0.08 (0.01)	0.04 (0.01)	0.07 (0.01)	0.06 (0.01)
35	7-epi-sesquithujene	1408	0.26 (0.02)	0.12 (0.02)	0.38 (0.02)	0.10 (0.02)	0.09 (0.01)
36	italicene	1409	0.04 (0.01)	0.08 (0.01)	0.07 (0.01)	0.08 (0.01)	0.14 (0.01)
37	9-aristolene	1423	0.02 (0.00)	0.03 (0.00)	0.04 (0.00)	0.05 (0.01)	0.05 (0.02)
38	1(10),8-aristoladiene	1429	2.91 (0.05)	4.93 (0.05)	4.65 (0.05)	1.90 (0.03)	6.07 (0.05)
39	204[M+](6) 107(100) 79(48)	1432	0.05 (0.01)	0.05 (0.01)	0.06 (0.01)	0.01 (0.00)	0.27 (0.02)
40	202[M+](4) 91(100) 185(89)	1434	-	-	-	-	-
41	204[M+](24) 91(100) 105(92)	1436	0.12 (0.02)	0.09 (0.01)	0.10 (0.01)	0.12 (0.02)	0.25 (0.01)
42	204[M+](18) 107(100) 161(88)	1438	0.04 (0.01)	0.07 (0.01)	0.05 (0.01)	0.11 (0.01)	8.71 (0.03)
43	204[M+](9) 119(100) 91(64)	1439	-	-	-	-	-
44	202[M+](30) 131(100) 159(62)	1440	6.71 (0.04)	7.57 (0.03)	6.16 (0.05)	5.53 (0.06)	0.76 (0.03)
45	202[M+](24) 69(100) 41(87)	1443	0.09 (0.01)	0.24 (0.01)	0.26 (0.01)	0.17 (0.02)	1.59 (0.04)
46	β-barbatene	1445	0.19 (0.01)	0.56 (0.01)	0.38 (0.01)	0.32 (0.03)	0.20 (0.01)
47	202[M+](19) 91(100) 41(85)	1450	0.10 (0.01)	0.26 (0.01)	0.17 (0.01)	0.17 (0.02)	0.49 (0.02)
48	202[M+](23) 91(100) 159(93)	1452	-	-	-	-	-
49	202[M+](23) 159(100) 131(74)	1455	2.16 (0.03)	2.48 (0.05)	2.09 (0.03)	1.71 (0.02)	3.67 (0.03)
50	204[M+](7) 159(100) 91(97)	1457	0.08 (0.02)	0.14 (0.02)	0.09 (0.02)	0.11 (0.01)	6.16 (0.05)
51	218[M+](26) 148(100) 133(75)	1466	-	-	-	-	-
52	204[M+](36) 119(100)93(61)	1469	6.03 (0.05)	3.49 (0.04)	5.75 (0.06)	5.58 (0.05)	3.16 (0.03)
53	γ-curcumene	1475	5.75 (0.04)	4.51 (0.05)	5.76 (0.04)	5.18 (0.06)	1.27 (0.02)
54	218[M+](12) 105(100) 91(92)	1476	-	-	-	-	-

55	α-curcumene	1477	2.49 (0.03)	0.47 (0.04)	2.83 (0.03)	1.87 (0.02)	4.38 (0.03)
56	218[M+](25) 105(100) 91(95)	1477	-	-	-	-	-
57	α-zingiberene	1479	0.32 (0.02)	1.23 (0.02)	0.62 (0.02)	0.09 (0.01)	0.28 (0.01)
58	bicyclogermacrene	1481	2.61 (0.03)	2.81 (0.05)	1.07 (0.04)	1.67 (0.03)	5.54 (0.04)
59	202[M+](29) 91(100) 133(92)	1500	0.25 (0.02)	0.31 (0.02)	0.24 (0.02)	0.34 (0.01)	2.14 (0.03)
60	γ-bisabolene	1505	0.59 (0.04)	1.01 (0.04)	0.88 (0.03)	0.55 (0.03)	2.24 (0.04)
61	202[M+](32) 133(100) 105(69)	1510	1.80 (0.04)	1.47 (0.04)	1.53 (0.04)	0.87 (0.03)	0.47 (0.03)
62	218[M+](29) 91(100) 93(97)	1513	-	-	-	-	0.02 (0.01)
63	218[M+](11) 132(100) 105(93)	1519	0.14 (0.01)	0.23 (0.01)	0.22 (0.01)	0.22 (0.02)	0.56 (0.03)
64	β-sesquiphellandrene	1524	1.48 (0.03)	1.11 (0.04)	1.57 (0.03)	0.91 (0.02)	1.99 (0.04)
65	218[M+](3) 159(100) 131(76)	1529	1.00 (0.04)	3.41 (0.05)	2.88 (0.05)	0.86 (0.03)	0.35 (0.02)
66	218[M+](24) 148(100) 133(63)	1532	-	-	-	-	-
67	220[M+](8) 85(100) 135(89)	1545	0.44 (0.03)	0.18 (0.02)	0.45 (0.03)	0.70 (0.02)	0.11 (0.01)
68	218[M+](4) 135(100) 107(42)	1548	-	-	-	-	-
69	202[M+](85) 131(100) 91(81)	1551	0.12 (0.02)	0.06 (0.02)	0.11 (0.02)	0.28 (0.03)	0.31 (0.02)
70	218[M+](6) 91(100) 157(90)	1554	-	-	-	-	-
71	218[M+](25) 145(100) 147(97)	1561	-	-	-	-	-
72	218[M+](4) 93(100) 43(75)	1568	-	-	-	-	-
73	4,5-dehydroviridiflorol	1572	2.15 (0.05)	2.14 (0.05)	1.66 (0.05)	0.40 (0.03)	0.13 (0.01)
74	222[M+](3) 43(100) 81(53)	1578	1.04 (0.03)	1.59 (0.03)	1.01 (0.04)	0.11 (0.01)	0.23 (0.01)
75	218[M+](6) 43(100) 93(57)	1579	0.09 (0.01)	0.50 (0.01)	0.24 (0.01)	0.17 (0.02)	1.16 (0.03)
76	218[M+](7) 43(100) 91(67)	1581	-	-	-	-	-
77	220[M+](14) 79(100) 93(93)	1584	0.15 (0.01)	0.12 (0.01)	0.39 (0.01)	0.49 (0.04)	0.58 (0.03)
78	220[M+](1) 94(100) 79(43)	1589	0.87 (0.03)	1.40 (0.03)	1.50 (0.03)	0.16 (0.02)	0.17 (0.02)
79	218[M+](25) 145(100) 147(87)	1594	-	-	-	-	-
80	bisabola-2,10-diene[1,9]oxide	1596	36.21 (0.06)	35.10 (0.06)	36.72 (0.07)	47.36 (0.06)	26.12 (0.07)
81	218[M+](1) 94(100) 79(52)	1605	-	-	-	-	-
82	218[M+](26) 145(100) 43(92)	1613	-	-	-	-	-
83	220[M+](2) 94(100) 79(39)	1625	2.17 (0.03)	2.36 (0.04)	2.72 (0.04)	4.88 (0.03)	2.63 (0.03)
84	218[M+](5) 145(100) 160(48)	1641	-	-	0.02 (0.00)	-	-
85	218[M+](18) 105(100) 120(83)	1646	0.21 (0.01)	0.45 (0.01)	0.20 (0.02)	0.39 (0.04)	0.05 (0.01)
86	218[M+](5) 135(100) 107(52)	1651	-	-	-	-	-
87	220[M+](2) 91(100) 43(91)	1658	-	-	-	-	-
88	220[M+](8) 159(100) 91(81)	1668	0.29 (0.02)	0.29 (0.02)	0.20 (0.02)	0.68 (0.03)	0.06 (0.01)
89	220[M+](5) 161(100) 91(69)	1670	-	-	-	-	-
90	218[M+](38) 145(100) 91(51)	1677	-	-	-	-	-
91	218[M+](2) 179(100) 161(92)	1686	-	-	-	-	-
92	218[M+](9) 105(100) 119(59)	1689	0.13 (0.01)	0.11 (0.01)	0.18 (0.01)	0.56 (0.04)	0.20 (0.02)
93	218[M+](22) 83(100) 94(92)	1699	0.17 (0.02)	0.16 (0.02)	0.15 (0.01)	0.39 (0.03)	0.02 (0.00)
94	218[M+](28) 135(100) 91(77)	1701	-	-	-	0.09 (0.01)	-
95	218[M+](20) 91(100) 133(98)	1706	0.17 (0.01)	0.14 (0.01)	0.16 (0.01)	0.01 (0.00)	0.11 (0.02)
96	220[M+](4) 110(100) 95(62)	1708	-	-	-	0.27 (0.02)	-
97	218[M+](11) 123(100) 95(62)	1712	0.07 (0.01)	0.06 (0.01)	0.07 (0.01)	0.01 (0.00)	0.12 (0.02)
98	220[M+](18) 83(100) 125(79)	1722	0.16 (0.01)	0.07 (0.01)	0.18 (0.01)	1.46 (0.03)	0.04 (0.01)
99	218[M+](2) 120(100) 83(34)	1729	0.15 (0.01)	0.13 (0.00)	0.09 (0.01)	0.17 (0.02)	0.04 (0.01)
100	218[M+](1) 121(100) 165(59)	1739	0.08 (0.01)	0.10 (0.01)	0.09 (0.01)	0.07 (0.01)	0.03 (0.01)
101	218[M+](2) 82(100) 41(50)	1744	0.07 (0.01)	0.04 (0.01)	0.07 (0.01)	0.14 (0.02)	0.07 (0.02)
102	218[M+](3) 82(100) 41(49)	1754	0.11 (0.01)	0.05 (0.01)	0.10 (0.01)	0.24 (0.02)	0.08 (0.02)
103	220[M+](9) 137(100) 135(78)	1759	0.12 (0.01)	0.10 (0.01)	0.16 (0.01)	0.32 (0.03)	0.07 (0.01)
104	218[M+](29) 136(100) 121(81)	1762	0.12 (0.01)	0.08 (0.01)	0.17 (0.01)	0.31 (0.03)	0.11 (0.02)
105	218[M+](1) 183(100) 198(61)	1795	0.08 (0.01)	0.07 (0.01)	0.08 (0.01)	0.42 (0.03)	0.11 (0.02)
106	218[M+](17) 82(100) 109(83)	1800	0.12 (0.01)	0.10 (0.01)	0.12 (0.01)	0.24 (0.02)	0.07 (0.01)
107	221[M+](1) 82(100) 67(39)	1808	0.08 (0.01)	0.06 (0.01)	0.07 (0.01)	0.13 (0.01)	-
	Total		95.37 (1.40)	96.30 (1.47)	98.07 (1.47)	96.27 (1.54)	96.24 (1.45)
	% Identified		69.44 (0.71)	67.90 (0.74)	69.47 (0.79)	67.57 (0.64)	60.70 (0.66)
	Including:						
	Aliphatics		0.95 (0.11)	1.40 (0.12)	1.45 (0.11)	0.41 (0.08)	0.18 (0.05)

Aromatics	5.46 (0.13)	3.90 (0.12)	4.29 (0.14)	1.60 (0.12)	1.25 (0.13)
Monoterpene hydrocarbons	0.03 (0.00)	0.02 (0.00)	0.02 (0.00)	0.03 (0.00)	0.04 (0.01)
Monoterpenoide hydrocarbons	0.02 (0.00)	0.07 (0.01)	0.03 (0.00)	-	0.03 (0.01)
Sesquiterpene hydrocarbons	24.62 (0.36)	25.27 (0.37)	25.30 (0.43)	17.78 (0.35)	32.95 (0.38)
Sesquiterpenoide hydrocarbons	38.36 (0.11)	37.24 (0.12)	38.38 (0.11)	47.76 (0.09)	26.25 (0.08)

- less than 0.01%. [a] Retention index on Quadrex 007-5MS column. [b] For abbreviations of samples see Table 1. ( ) standard deviation.

Table S1b. Volatile compounds detected in the samples CSU1-6 – CSU1-10.

No.	Compounds	RI [a]	Code [b]				
			**CSU1-6**	**CSU1-7**	**CSU1-8**	**CSU1-9**	**CSU1-10**
1	hexanal	782	-	0.01 (0.00)	0.04 (0.01)	0.02 (0.01)	0.02 (0.01)
2	3-methylbutanoic acid	817	-	-	0.02 (0.00)	0.01 (0.00)	0.01 (0.01)
3	2-methylbutanoic acid	832	-	-	-	-	-
4	3-hexen-1-ol	858	0.01 (0.01)	0.05 (0.01)	-	-	-
5	1-hexanol	867	-	-	0.17 (0.03)	0.09 (0.02)	0.09 (0.02)
6	tricyclene	927	0.02 (0.01)	0.01 (0.00)	-	-	-
7	α-pinene	936	0.03 (0.01)	0.01 (0.00)	0.01 (0.00)	0.01 (0.00)	0.01 (0.01)
8	86[M+](50) 42(100) 86(38)	957	0.01 (0.00)	0.01 (0.00)	0.02 (0.00)	0.01 (0.00)	0.02 (0.01)
9	hexanoic acid	975	0.02 (0.01)	0.01 (0.00)	0.07 (0.01)	0.04 (0.01)	0.04 (0.01)
10	β-pinene	975	0.02 (0.00)	0.02 (0.00)	0.04 (0.01)	0.02 (0.01)	0.03 (0.01)
11	1-octen-3-ol	979	0.05 (0.02)	0.04 (0.01)	0.11 (0.02)	0.03 (0.01)	0.08 (0.02)
12	3-octanone	985	0.08 (0.02)	0.02 (0.01)	0.04 (0.01)	0.07 (0.02)	0.04 (0.01)
13	3-octanol	994	0.01 (0.00)	0.01 (0.00)	0.01 (0.00)	-	-
14	benzenemethanol	1033	0.22 (0.04)	0.17 (0.03)	0.06 (0.01)	0.04 (0.01)	0.23 (0.01)
15	benzeneacetaldehyde	1043	0.03 (0.00)	0.08 (0.01)	0.25 (0.03)	0.10 (0.02)	0.09 (0.02)
16	2-ethylhexanoic acid	1108	0.01 (0.00)	0.03 (0.00)	0.04 (0.01)	0.03 (0.01)	0.06 (0.01)
17	benzeneethanol	1116	0.33 (0.04)	0.91 (0.02)	0.30 (0.04)	0.44 (0.03)	0.58 (0.03)
18	126[M+](11) 55(100) 98(84)	1154	-	-	0.09 (0.01)	0.06 (0.01)	0.06 (0.01)
19	122[M+](20) 91(100) 44(58)	1164	-	-	-	-	-
20	140[M+](4) 43(100) 57(60)	1200	0.03 (0.01)	0.03 (0.01)	0.02 (0.00)	0.01 (0.00)	0.01 (0.01)
21	128[M+](5) 44(100) 57(63)	1203	0.41 (0.02)	0.43 (0.02)	0.01 (0.00)	0.03 (0.01)	0.01 (0.01)
22	152[M+](92) 67(100) 109(98)	1217	0.10 (0.02)	0.15 (0.02)	0.67 (0.06)	0.14 (0.05)	0.26 (0.04)
23	phenoxyethanol	1223	0.32 (0.05)	0.19 (0.06)	0.22 (0.03)	0.45 (0.03)	0.50 (0.03)
24	1-phenoxy-2-propanol	1247	-	-	0.05 (0.01)	0.07 (0.02)	0.04 (0.01)
25	144[M+](38) 44(100) 129(72)	1258	-	-	0.02 (0.00)	0.03 (0.01)	0.02 (0.01)
26	bornyl acetate	1285	-	-	-	-	-
27	isobornyl acetate	1290	0.06 (0.02)	0.08 (0.01)	-	-	-
28	189(8) 121(100) 93(82)	1320	0.02 (0.00)	0.02 (0.00)	0.05 (0.01)	0.04 (0.01)	0.02 (0.01)
29	bicycloelemene	1341	1.05 (0.04)	1.55 (0.04)	0.92 (0.04)	0.70 (0.03)	1.35 (0.04)
30	δ-elemene	1343	0.03 (0.00)	0.03 (0.00)	-	-	-
31	204[M+](17) 81(100) 93(83)	1355	-	-	-	-	-
32	anastreptene	1370	6.90 (0.05)	8.77 (0.04)	7.35 (0.07)	7.73 (0.05)	6.47 (0.07)
33	α-funebrene	1385	0.21 (0.02)	0.26 (0.02)	0.49 (0.03)	0.47 (0.03)	0.32 (0.03)
34	β-elemene	1394	0.02 (0.01)	0.05 (0.01)	0.12 (0.01)	0.09 (0.01)	0.09 (0.01)
35	7-epi-sesquithujene	1408	0.08 (0.01)	0.07 (0.01)	0.14 (0.02)	0.54 (0.03)	0.13 (0.01)
36	italicene	1409	0.12 (0.01)	0.15 (0.01)	0.07 (0.01)	0.10 (0.02)	0.15 (0.02)
37	9-aristolene	1423	0.07 (0.02)	0.23 (0.02)	0.07 (0.01)	0.06 (0.01)	0.06 (0.03)
38	1(10),8-aristoladiene	1429	5.50 (0.06)	5.31 (0.05)	3.98 (0.04)	3.49 (0.05)	4.19 (0.04)
39	204[M+](6) 107(100) 79(48)	1432	0.18 (0.02)	0.41 (0.02)	0.07 (0.02)	0.03 (0.02)	0.04 (0.02)
40	202[M+](4) 91(100) 185(89)	1434	-	-	-	-	-
41	204[M+](24) 91(100) 105(92)	1436	0.19 (0.01)	0.35 (0.01)	0.21 (0.02)	0.23 (0.03)	0.12 (0.02)
42	204[M+](18) 107(100) 161(88)	1438	7.24 (0.05)	8.16 (0.06)	0.20 (0.02)	0.24 (0.02)	0.08 (0.01)
43	204[M+](9) 119(100) 91(64)	1439	-	-	-	-	-
44	202[M+](30) 131(100) 159(62)	1440	0.60 (0.03)	0.71 (0.02)	6.85 (0.04)	8.06 (0.05)	8.10 (0.03)
45	202[M+](24) 69(100) 41(87)	1443	0.95 (0.04)	1.61 (0.03)	0.58 (0.03)	0.51 (0.03)	0.17 (0.02)
46	β-barbatene	1445	0.12 (0.01)	0.11 (0.01)	0.93 (0.03)	1.01 (0.04)	0.35 (0.03)
47	202[M+](19) 91(100) 41(85)	1450	0.35 (0.02)	0.56 (0.02)	0.14 (0.02)	0.30 (0.02)	0.11 (0.02)
48	202[M+](23) 91(100) 159(93)	1452	-	-	-	-	-
49	202[M+](23) 159(100) 131(74)	1455	2.21 (0.05)	3.10 (0.03)	1.58 (0.04)	2.88 (0.03)	2.28 (0.04)
50	204[M+](7) 159(100) 91(97)	1457	8.40 (0.06)	6.46 (0.05)	0.26 (0.02)	0.11 (0.02)	0.03 (0.02)
51	218[M+](26) 148(100) 133(75)	1466	-	-	-	-	-
52	204[M+](36) 119(100)93(61)	1469	2.49 (0.04)	6.43 (0.04)	6.31 (0.08)	8.11 (0.09)	6.32 (0.10)
53	γ-curcumene	1475	1.83 (0.02)	1.73 (0.03)	5.03 (0.05)	7.42 (0.06)	5.46 (0.06)
54	218[M+](12) 105(100) 91(92)	1476	-	-	-	-	-

55	α-curcumene	1477	5.18 (0.03)	1.54 (0.02)	3.45 (0.03)	3.90 (0.04)	2.45 (0.03)
56	218[M+](25) 105(100) 91(95)	1477	-	-	-	-	-
57	α-zingiberene	1479	0.31 (0.01)	0.36 (0.01)	1.04 (0.02)	1.32 (0.03)	0.38 (0.02)
58	bicyclogermacrene	1481	6.48 (0.05)	3.82 (0.04)	3.46 (0.04)	4.10 (0.04)	2.30 (0.04)
59	202[M+](29) 91(100) 133(92)	1500	2.25 (0.02)	1.37 (0.02)	4.18 (0.05)	1.05 (0.04)	3.34 (0.06)
60	γ-bisabolene	1505	3.10 (0.04)	3.39 (0.04)	1.56 (0.03)	1.35 (0.03)	0.97 (0.03)
61	202[M+](32) 133(100) 105(69)	1510	0.45 (0.03)	1.75 (0.04)	2.24 (0.02)	2.45 (0.05)	1.23 (0.03)
62	218[M+](29) 91(100) 93(97)	1513	-	-	0.09 (0.01)	0.08 (0.01)	0.04 (0.01)
63	218[M+](11) 132(100) 105(93)	1519	0.81 (0.03)	1.29 (0.04)	0.40 (0.03)	0.27 (0.02)	0.16 (0.02)
64	β-sesquiphellandrene	1524	1.28 (0.03)	1.51 (0.03)	1.49 (0.04)	1.69 (0.03)	1.15 (0.05)
65	218[M+](3) 159(100) 131(76)	1529	0.25 (0.02)	0.31 (0.02)	0.97 (0.02)	1.38 (0.03)	1.33 (0.02)
66	218[M+](24) 148(100) 133(63)	1532	-	-	-	-	-
67	220[M+](8) 85(100) 135(89)	1545	0.05 (0.01)	0.09 (0.01)	0.54 (0.03)	0.51 (0.02)	0.48 (0.02)
68	218[M+](4) 135(100) 107(42)	1548	-	-	-	-	-
69	202[M+](85) 131(100) 91(81)	1551	0.40 (0.02)	0.34 (0.02)	0.28 (0.02)	0.32 (0.03)	0.13 (0.03)
70	218[M+](6) 91(100) 157(90)	1554	-	-	-	-	-
71	218[M+](25) 145(100) 147(97)	1561	-	-	-	-	-
72	218[M+](4) 93(100) 43(75)	1568	-	-	-	-	-
73	4,5-dehydroviridiflorol	1572	0.04 (0.01)	0.02 (0.01)	0.48 (0.03)	0.77 (0.03)	0.87 (0.03)
74	222[M+](3) 43(100) 81(53)	1578	0.12 (0.01)	0.37 (0.02)	0.13 (0.02)	0.15 (0.02)	0.13 (0.02)
75	218[M+](6) 43(100) 93(57)	1579	0.33 (0.03)	0.12 (0.03)	0.18 (0.02)	0.08 (0.02)	0.13 (0.02)
76	218[M+](7) 43(100) 91(67)	1581	-	-	-	-	-
77	220[M+](14) 79(100) 93(93)	1584	0.92 (0.03)	0.75 (0.03)	0.11 (0.01)	0.41 (0.02)	0.15 (0.01)
78	220[M+](1) 94(100) 79(43)	1589	0.15 (0.02)	0.22 (0.02)	1.74 (0.03)	1.11 (0.04)	0.66 (0.04)
79	218[M+](25) 145(100) 147(87)	1594	-	-	-	-	-
80	bisabola-2,10-diene[1,9]oxide	1596	32.66 (0.09)	26.66 (0.08)	34.23 (0.09)	28.47 (0.10)	38.56 (0.11)
81	218[M+](1) 94(100) 79(52)	1605	-	-	-	-	-
82	218[M+](26) 145(100) 43(92)	1613	-	-	-	-	-
83	220[M+](2) 94(100) 79(39)	1625	2.35 (0.04)	2.32 (0.04)	1.23 (0.03)	2.19 (0.05)	2.66 (0.05)
84	218[M+](5) 145(100) 160(48)	1641	-	-	-	-	-
85	218[M+](18) 105(100) 120(83)	1646	0.03 (0.01)	0.04 (0.01)	0.19 (0.02)	0.05 (0.02)	0.03 (0.01)
86	218[M+](5) 135(100) 107(52)	1651	-	-	-	-	-
87	220[M+](2) 91(100) 43(91)	1658	-	-	-	-	-
88	220[M+](8) 159(100) 91(81)	1668	0.13 (0.02)	0.12 (0.01)	0.26 (0.03)	0.43 (0.02)	0.25 (0.04)
89	220[M+](5) 161(100) 91(69)	1670	-	-	-	-	-
90	218[M+](38) 145(100) 91(51)	1677	-	-	-	-	-
91	218[M+](2) 179(100) 161(92)	1686	-	-	-	-	-
92	218[M+](9) 105(100) 119(59)	1689	0.08 (0.01)	0.12 (0.02)	0.18 (0.02)	0.20 (0.02)	0.16 (0.01)
93	218[M+](22) 83(100) 94(92)	1699	0.02 (0.01)	0.03 (0.00)	0.23 (0.02)	0.21 (0.02)	0.12 (0.02)
94	218[M+](28) 135(100) 91(77)	1701	-	-	0.07 (0.01)	0.06 (0.01)	0.03 (0.01)
95	218[M+](20) 91(100) 133(98)	1706	0.07 (0.02)	0.09 (0.02)	0.05 (0.01)	0.13 (0.01)	0.07 (0.01)
96	220[M+](4) 110(100) 95(62)	1708	-	-	0.14 (0.02)	0.10 (0.02)	0.15 (0.01)
97	218[M+](11) 123(100) 95(62)	1712	0.08 (0.02)	0.12 (0.02)	-	-	-
98	220[M+](18) 83(100) 125(79)	1722	0.03 (0.01)	0.04 (0.01)	0.15 (0.03)	0.17 (0.02)	0.16 (0.01)
99	218[M+](2) 120(100) 83(34)	1729	0.01 (0.01)	0.03 (0.01)	0.07 (0.02)	0.05 (0.02)	0.08 (0.01)
100	218[M+](1) 121(100) 165(59)	1739	0.02 (0.01)	0.04 (0.01)	0.06 (0.02)	0.06 (0.02)	0.08 (0.02)
101	218[M+](2) 82(100) 41(50)	1744	0.03 (0.02)	0.07 (0.02)	0.08 (0.02)	0.08 (0.02)	0.06 (0.02)
102	218[M+](3) 82(100) 41(49)	1754	0.08 (0.02)	0.10 (0.02)	0.11 (0.02)	0.15 (0.02)	0.08 (0.02)
103	220[M+](9) 137(100) 135(78)	1759	0.03 (0.01)	0.06 (0.02)	0.14 (0.03)	0.13 (0.03)	0.14 (0.03)
104	218[M+](29) 136(100) 121(81)	1762	0.05 (0.02)	0.07 (0.02)	0.07 (0.01)	0.11 (0.01)	0.12 (0.01)
105	218[M+](1) 183(100) 198(61)	1795	0.06 (0.02)	0.09 (0.02)	0.07 (0.01)	0.24 (0.02)	0.11 (0.01)
106	218[M+](17) 82(100) 109(83)	1800	0.03 (0.01)	0.05 (0.01)	0.15 (0.02)	0.15 (0.02)	0.12 (0.02)
107	221[M+](1) 82(100) 67(39)	1808	-	-	0.09 (0.01)	0.09 (0.01)	0.06 (0.01)
	Total		98.21 (1.59)	95.66 (1.47)	97.55 (1.79)	97.87 (1.89)	97.01 (1.84)
	% Identified		66.19 (0.74)	57.21 (0.63)	66.26 (0.81)	64.66 (0.83)	67.08 (0.86)
	Including:						
	Aliphatics		0.18 (0.06)	0.17 (0.03)	0.50 (0.09)	0.29 (0.08)	0.34 (0.09)

Aromatics	0.90 (0.13)	1.35 (0.12)	0.88 (0.12)	1.10 (0.11)	1.44 (0.10)
Monoterpene hydrocarbons	0.07 (0.02)	0.04 (0.00)	0.05 (0.01)	0.03 (0.01)	0.04 (0.02)
Monoterpenoide hydrocarbons	0.06 (0.02)	0.08 (0.01)	-	-	-
Sesquiterpene hydrocarbons	32.28 (0.41)	28.89 (0.38)	30.11 (0.47)	34.00 (0.50)	25.83 (0.51)
Sesquiterpenoide hydrocarbons	32.70 (0.10)	26.68 (0.09)	34.71 (0.12)	29.24 (0.13)	39.43 (0.14)

- less than 0.01%. [a] Retention index on Quadrex 007-5MS column. [b] For abbreviations of samples see Table 1. ( ) standard deviation.

Table S1c. Volatile compounds detected in the samples CSU1-11 – CSU1-15.

No.	Compounds	RI [a]	Code [b]				
			CSU1-11	CSU1-12	CSU1-13	CSU1-14	CSU1-15
1	hexanal	782	-	0.02 (0.01)	0.02 (0.01)	0.02 (0.01)	0.03 (0.01)
2	3-methylbutanoic acid	817	0.03 (0.01)	0.05 (0.00)	0.02 (0.00)	0.03 (0.00)	0.06 (0.02)
3	2-methylbutanoic acid	832	-	-	-	-	-
4	3-hexen-1-ol	858	-	-	-	-	-
5	1-hexanol	867	0.05 (0.03)	0.21 (0.03)	0.03 (0.03)	0.05 (0.03)	0.02 (0.01)
6	tricyclene	927	-	-	-	-	-
7	α-pinene	936	0.01 (0.00)	0.01 (0.00)	0.01 (0.00)	0.01 (0.00)	0.03 (0.00)
8	86[M+](50) 42(100) 86(38)	957	0.01 (0.00)	0.04 (0.00)	0.02 (0.00)	0.02 (0.00)	0.02 (0.00)
9	hexanoic acid	975	0.06 (0.01)	0.13 (0.01)	0.05 (0.01)	0.04 (0.01)	0.10 (0.01)
10	β-pinene	975	0.01 (0.01)	0.03 (0.01)	0.02 (0.01)	0.01 (0.01)	0.01 (0.01)
11	1-octen-3-ol	979	0.05 (0.02)	0.05 (0.02)	0.04 (0.02)	0.04 (0.02)	0.03 (0.02)
12	3-octanone	985	0.05 (0.01)	0.12 (0.01)	0.07 (0.01)	0.06 (0.01)	0.13 (0.01)
13	3-octanol	994	-	0.01 (0.00)	0.01 (0.00)	0.01 (0.00)	0.01 (0.00)
14	benzenemethanol	1033	0.09 (0.01)	0.06 (0.01)	0.04 (0.01)	0.05 (0.01)	0.05 (0.01)
15	benzeneacetaldehyde	1043	0.15 (0.03)	0.21 (0.03)	0.12 (0.03)	0.18 (0.03)	0.10 (0.03)
16	2-ethylhexanoic acid	1108	0.08 (0.01)	0.17 (0.01)	0.09 (0.01)	0.10 (0.01)	0.04 (0.01)
17	benzeneethanol	1116	0.44 (0.04)	1.14 (0.04)	0.59 (0.04)	0.56 (0.04)	1.39 (0.04)
18	126[M+](11) 55(100) 98(84)	1154	0.03 (0.01)	0.09 (0.01)	0.04 (0.01)	0.04 (0.01)	0.03 (0.01)
19	122[M+](20) 91(100) 44(58)	1164	-	-	-	-	-
20	140[M+](4) 43(100) 57(60)	1200	0.02 (0.00)	0.01 (0.00)	0.03 (0.00)	0.01 (0.00)	0.06 (0.00)
21	128[M+](5) 44(100) 57(63)	1203	0.05 (0.00)	0.04 (0.00)	0.15 (0.00)	0.03 (0.00)	0.04 (0.00)
22	152[M+](92) 67(100) 109(98)	1217	0.36 (0.06)	0.21 (0.06)	0.30 (0.06)	0.59 (0.06)	0.14 (0.06)
23	phenoxyethanol	1223	0.22 (0.03)	0.29 (0.03)	0.14 (0.03)	0.26 (0.03)	0.36 (0.03)
24	1-phenoxy-2-propanol	1247	0.06 (0.01)	0.03 (0.01)	0.02 (0.01)	0.03 (0.01)	0.02 (0.01)
25	144[M+](38) 44(100) 129(72)	1258	0.05 (0.00)	0.01 (0.00)	0.06 (0.00)	0.05 (0.00)	0.03 (0.00)
26	bornyl acetate	1285	-	-	-	-	-
27	isobornyl acetate	1290	-	-	-	-	-
28	189(8) 121(100) 93(82)	1320	0.06 (0.01)	0.01 (0.01)	0.05 (0.01)	0.07 (0.01)	0.05 (0.01)
29	bicycloelemene	1341	1.40 (0.04)	1.26 (0.04)	1.02 (0.04)	1.41 (0.04)	1.20 (0.04)
30	δ-elemene	1343	-	-	-	-	-
31	204[M+](17) 81(100) 93(83)	1355	-	-	-	-	-
32	anastreptene	1370	7.98 (0.07)	5.10 (0.07)	7.25 (0.07)	9.71 (0.07)	5.12 (0.07)
33	α-funebrene	1385	0.82 (0.03)	0.37 (0.03)	0.66 (0.03)	0.59 (0.03)	0.24 (0.03)
34	β-elemene	1394	0.22 (0.01)	0.06 (0.01)	0.13 (0.01)	0.20 (0.01)	0.05 (0.01)
35	7-epi-sesquithujene	1408	0.08 (0.02)	0.05 (0.02)	0.12 (0.02)	0.06 (0.02)	0.16 (0.02)
36	italicene	1409	0.14 (0.01)	0.24 (0.01)	0.10 (0.01)	0.08 (0.01)	0.13 (0.01)
37	9-aristolene	1423	0.07 (0.01)	0.04 (0.01)	0.04 (0.01)	0.09 (0.01)	0.05 (0.01)
38	1(10),8-aristoladiene	1429	6.50 (0.04)	2.59 (0.04)	5.31 (0.04)	7.14 (0.04)	2.12 (0.04)
39	204[M+](6) 107(100) 79(48)	1432	0.12 (0.02)	0.06 (0.02)	0.07 (0.02)	0.08 (0.02)	0.05 (0.02)
40	202[M+](4) 91(100) 185(89)	1434	-	-	-	-	-
41	204[M+](24) 91(100) 105(92)	1436	0.23 (0.02)	0.05 (0.02)	0.17 (0.02)	0.26 (0.02)	0.13 (0.02)
42	204[M+](18) 107(100) 161(88)	1438	0.22 (0.02)	0.06 (0.02)	0.12 (0.02)	0.15 (0.02)	0.12 (0.02)
43	204[M+](9) 119(100) 91(64)	1439	-	-	-	-	-
44	202[M+](30) 131(100) 159(62)	1440	7.01 (0.04)	5.98 (0.04)	6.32 (0.04)	6.20 (0.04)	8.26 (0.04)
45	202[M+](24) 69(100) 41(87)	1443	0.87 (0.03)	0.25 (0.03)	0.75 (0.03)	0.76 (0.03)	0.25 (0.03)
46	β-barbatene	1445	1.30 (0.03)	0.19 (0.03)	1.17 (0.03)	0.55 (0.03)	0.67 (0.03)
47	202[M+](19) 91(100) 41(85)	1450	0.46 (0.02)	0.09 (0.02)	0.36 (0.02)	0.38 (0.02)	0.30 (0.02)
48	202[M+](23) 91(100) 159(93)	1452	-	-	-	-	-
49	202[M+](23) 159(100) 131(74)	1455	2.76 (0.04)	1.27 (0.04)	2.51 (0.04)	1.83 (0.04)	2.59 (0.04)
50	204[M+](7) 159(100) 91(97)	1457	0.50 (0.02)	0.15 (0.02)	0.41 (0.02)	0.53 (0.02)	0.19 (0.02)
51	218[M+](26) 148(100) 133(75)	1466	-	-	-	-	-
52	204[M+](36) 119(100)93(61)	1469	5.13 (0.08)	5.59 (0.08)	7.38 (0.08)	6.78 (0.08)	5.22 (0.08)
53	γ-curcumene	1475	4.83 (0.05)	7.73 (0.05)	7.14 (0.05)	6.50 (0.05)	7.60 (0.05)
54	218[M+](12) 105(100) 91(92)	1476	-	-	-	-	-

55	α-curcumene	1477	2.36 (0.03)	2.73 (0.03)	2.60 (0.03)	1.86 (0.03)	1.86 (0.03)
56	218[M+](25) 105(100) 91(95)	1477	-	-	-	-	-
57	α-zingiberene	1479	0.82 (0.03)	0.43 (0.03)	0.78 (0.03)	0.85 (0.03)	0.32 (0.03)
58	bicyclogermacrene	1481	4.50 (0.05)	2.74 (0.05)	3.56 (0.05)	3.48 (0.05)	2.93 (0.05)
59	202[M+](29) 91(100) 133(92)	1500	5.66 (0.04)	0.80 (0.03)	3.13 (0.04)	3.92 (0.04)	0.50 (0.03)
60	γ-bisabolene	1505	1.43 (0.02)	0.62 (0.02)	0.70 (0.05)	0.98 (0.03)	0.91 (0.02)
61	202[M+](32) 133(100) 105(69)	1510	2.32 (0.03)	1.40 (0.04)	2.37 (0.06)	1.64 (0.02)	1.12 (0.03)
62	218[M+](29) 91(100) 93(97)	1513	0.06 (0.02)	0.07 (0.01)	0.05 (0.01)	0.06 (0.01)	0.07 (0.01)
63	218[M+](11) 132(100) 105(93)	1519	0.42 (0.03)	0.08 (0.01)	0.42 (0.02)	0.51 (0.02)	0.02 (0.01)
64	β-sesquiphellandrene	1524	1.66 (0.05)	1.33 (0.03)	1.61 (0.05)	1.25 (0.05)	1.87 (0.05)
65	218[M+](3) 159(100) 131(76)	1529	1.21 (0.03)	1.49 (0.05)	1.00 (0.04)	1.35 (0.03)	2.38 (0.05)
66	218[M+](24) 148(100) 133(63)	1532	-	-	-	-	-
67	220[M+](8) 85(100) 135(89)	1545	0.34 (0.04)	0.31 (0.03)	0.37 (0.03)	0.33 (0.02)	0.29 (0.03)
68	218[M+](4) 135(100) 107(42)	1548	-	-	-	-	-
69	202[M+](85) 131(100) 91(81)	1551	0.26 (0.02)	0.15 (0.01)	0.49 (0.04)	0.38 (0.03)	0.10 (0.01)
70	218[M+](6) 91(100) 157(90)	1554	-	-	-	-	-
71	218[M+](25) 145(100) 147(97)	1561	-	-	-	-	-
72	218[M+](4) 93(100) 43(75)	1568	-	-	-	-	-
73	4,5-dehydroviridiflorol	1572	0.35 (0.02)	0.40 (0.02)	0.40 (0.02)	0.64 (0.04)	0.95 (0.04)
74	222[M+](3) 43(100) 81(53)	1578	0.16 (0.02)	0.12 (0.01)	0.15 (0.03)	0.09 (0.01)	0.28 (0.03)
75	218[M+](6) 43(100) 93(57)	1579	0.15 (0.02)	0.20 (0.03)	0.05 (0.01)	0.24 (0.02)	0.25 (0.02)
76	218[M+](7) 43(100) 91(67)	1581	-	-	-	-	-
77	220[M+](14) 79(100) 93(93)	1584	0.22 (0.02)	0.30 (0.03)	0.12 (0.01)	0.10 (0.01)	0.17 (0.02)
78	220[M+](1) 94(100) 79(43)	1589	1.33 (0.04)	1.87 (0.05)	1.70 (0.05)	1.59 (0.03)	2.55 (0.07)
79	218[M+](25) 145(100) 147(87)	1594	-	-	-	-	-
80	bisabola-2,10-diene[1,9]oxide	1596	30.39 (0.11)	44.14 (0.10)	33.35 (0.12)	31.10 (0.13)	40.69 (0.12)
81	218[M+](1) 94(100) 79(52)	1605	-	-	-	-	-
82	218[M+](26) 145(100) 43(92)	1613	-	-	-	-	-
83	220[M+](2) 94(100) 79(39)	1625	0.75 (0.04)	3.23 (0.04)	0.87 (0.04)	0.86 (0.02)	2.90 (0.04)
84	218[M+](5) 145(100) 160(48)	1641	-	-	-	-	-
85	218[M+](18) 105(100) 120(83)	1646	0.06 (0.01)	0.12 (0.04)	0.05 (0.03)	0.09 (0.02)	0.17 (0.02)
86	218[M+](5) 135(100) 107(52)	1651	-	-	-	-	-
87	220[M+](2) 91(100) 43(91)	1658	-	-	-	-	-
88	220[M+](8) 159(100) 91(81)	1668	0.19 (0.03)	0.16 (0.05)	0.14 (0.02)	0.20 (0.04)	0.22 (0.04)
89	220[M+](5) 161(100) 91(69)	1670	-	-	-	-	-
90	218[M+](38) 145(100) 91(51)	1677	-	-	-	-	-
91	218[M+](2) 179(100) 161(92)	1686	-	-	-	-	-
92	218[M+](9) 105(100) 119(59)	1689	0.21 (0.03)	0.18 (0.03)	0.20 (0.03)	0.20 (0.03)	0.17 (0.03)
93	218[M+](22) 83(100) 94(92)	1699	0.23 (0.02)	0.14 (0.03)	0.23 (0.05)	0.23 (0.02)	0.14 (0.02)
94	218[M+](28) 135(100) 91(77)	1701	0.05 (0.01)	0.05 (0.02)	0.05 (0.01)	0.06 (0.01)	0.06 (0.01)
95	218[M+](20) 91(100) 133(98)	1706	0.06 (0.01)	0.14 (0.02)	0.05 (0.01)	0.04 (0.01)	0.08 (0.01)
96	220[M+](4) 110(100) 95(62)	1708	0.10 (0.01)	0.09 (0.01)	0.12 (0.01)	0.10 (0.02)	0.08 (0.02)
97	218[M+](11) 123(100) 95(62)	1712	-	-	-	-	-
98	220[M+](18) 83(100) 125(79)	1722	0.13 (0.02)	0.10 (0.01)	0.15 (0.02)	0.13 (0.02)	0.08 (0.02)
99	218[M+](2) 120(100) 83(34)	1729	0.06 (0.02)	0.06 (0.01)	0.06 (0.02)	0.06 (0.01)	0.02 (0.01)
100	218[M+](1) 121(100) 165(59)	1739	0.03 (0.01)	0.10 (0.02)	0.06 (0.01)	0.04 (0.01)	0.05 (0.01)
101	218[M+](2) 82(100) 41(50)	1744	0.04 (0.01)	0.08 (0.01)	0.07 (0.01)	0.05 (0.01)	0.03 (0.01)
102	218[M+](3) 82(100) 41(49)	1754	0.07 (0.01)	0.09 (0.01)	0.08 (0.01)	0.08 (0.00)	0.03 (0.01)
103	220[M+](9) 137(100) 135(78)	1759	0.11 (0.03)	0.12 (0.03)	0.14 (0.02)	0.14 (0.03)	0.06 (0.02)
104	218[M+](29) 136(100) 121(81)	1762	0.05 (0.02)	0.14 (0.02)	0.07 (0.01)	0.06 (0.01)	0.09 (0.01)
105	218[M+](1) 183(100) 198(61)	1795	0.05 (0.02)	0.06 (0.01)	0.06 (0.01)	0.08 (0.01)	0.13 (0.01)
106	218[M+](17) 82(100) 109(83)	1800	0.11 (0.02)	0.16 (0.01)	0.12 (0.01)	0.13 (0.01)	0.04 (0.01)
107	221[M+](1) 82(100) 67(39)	1808	0.06 (0.02)	0.11 (0.01)	0.06 (0.01)	0.06 (0.01)	0.02 (0.01)
	Total		98.55 (1.86)	98.41 (1.86)	98.42 (1.92)	98.59 (1.79)	98.86 (1.86)
	% Identified		66.17 (0.84)	72.56 (0.81)	67.23 (0.88)	67.97 (0.89)	69.26 (0.87)
	Including:						
	Aliphatics		0.32 (0.09)	0.76 (0.09)	0.33 (0.09)	0.35 (0.09)	0.42 (0.09)

Aromatics	0.96 (0.12)	1.73 (0.12)	0.91 (0.12)	1.08 (0.12)	1.92 (0.12)
Monoterpene hydrocarbons	0.02 (0.01)	0.04 (0.01)	0.03 (0.01)	0.02 (0.01)	0.04 (0.01)
Monoterpenoide hydrocarbons	-	-	-	-	-
Sesquiterpene hydrocarbons	34.12 (0.49)	25.48 (0.47)	32.21 (0.52)	34.77 (0.50)	25.23 (0.49)
Sesquiterpenoide hydrocarbons	30.74 (0.13)	44.54 (0.12)	33.75 (0.14)	31.74 (0.17)	41.64 (0.16)

- less than 0.01%. [a] Retention index on Quadrex 007-5MS column. [b] For abbreviations of samples see Table 1. ( ) standard deviation.

Table S1d. Volatile compounds detected in the samples CSU1-16 – CSU2-4.

No.	Compounds	RI [a]	Code [b]				
			CSU1-16	CSU2-1	CSU2-2	CSU2-3	CSU2-4
1	hexanal	782	0.03 (0.01)	0.09 (0.01)	0.02 (0.01)	0.01(0.01)	-
2	3-methylbutanoic acid	817	0.02 (0.00)	-	-	-	-
3	2-methylbutanoic acid	832	-	-	-	-	-
4	3-hexen-1-ol	858	-	-	-	-	0.02 (0.01)
5	1-hexanol	867	0.13 (0.03)	0.34 (0.03)	0.08 (0.01)	0.03(0.01)	-
6	tricyclene	927	-	0.02 (0.01)	-	-	0.06 (0.01)
7	α-pinene	936	0.01 (0.00)	0.05 (0.01)	0.01 (0.00)	0.01(0.01)	0.02 (0.01)
8	86[M+](50) 42(100) 86(38)	957	0.01 (0.00)	0.07 (0.01)	0.02 (0.01)	0.02(0.01)	0.02 (0.01)
9	hexanoic acid	975	0.07 (0.01)	0.01 (0.00)	-	-	-
10	β-pinene	975	0.01 (0.01)	0.05 (0.02)	0.02 (0.01)	0.02(0.01)	0.03 (0.01)
11	1-octen-3-ol	979	0.04 (0.02)	0.34 (0.04)	0.04 (0.01)	0.05(0.01)	0.02 (0.01)
12	3-octanone	985	0.17 (0.01)	0.16 (0.02)	0.08 (0.01)	0.07(0.01)	0.07 (0.01)
13	3-octanol	994	-	0.45 (0.03)	0.09 (0.01)	0.12(0.02)	0.15 (0.02)
14	benzenemethanol	1033	0.09 (0.01)	0.21 (0.02)	0.18 (0.03)	0.15(0.02)	0.24 (0.03)
15	benzeneacetaldehyde	1043	0.16 (0.03)	0.79 (0.04)	0.23 (0.03)	0.14(0.02)	0.03 (0.01)
16	2-ethylhexanoic acid	1108	0.10 (0.01)	0.26 (0.02)	0.06 (0.02)	0.04(0.01)	0.02 (0.01)
17	benzeneethanol	1116	0.25 (0.04)	1.79 (0.05)	0.60 (0.03)	0.36(0.03)	1.37 (0.05)
18	126[M+](11) 55(100) 98(84)	1154	0.07 (0.01)	0.05 (0.01)	-	-	-
19	122[M+](20) 91(100) 44(58)	1164	-	0.03 (0.01)	0.02 (0.01)	0.01 (0.00)	0.02 (0.01)
20	140[M+](4) 43(100) 57(60)	1200	0.01 (0.00)	0.14 (0.02)	0.01(0.01)	0.01 (0.01)	0.02 (0.01)
21	128[M+](5) 44(100) 57(63)	1203	0.03 (0.00)	-	0.01(0.00)	0.01 (0.01)	0.07 (0.01)
22	152[M+](92) 67(100) 109(98)	1217	0.14 (0.06)	1.12 (0.03)	0.06(0.01)	0.07 (0.01)	0.14 (0.02)
23	phenoxyethanol	1223	0.40 (0.03)	0.20 (0.01)	0.03(0.01)	0.02 (0.01)	-
24	1-phenoxy-2-propanol	1247	0.04 (0.01)	0.07 (0.01)	0.10(0.02)	0.11 (0.02)	0.01 (0.01)
25	144[M+](38) 44(100) 129(72)	1258	0.02 (0.00)	0.11 (0.01)	0.02(0.01)	0.03 (0.01)	-
26	bornyl acetate	1285	-	0.10 (0.01)	-	-	-
27	isobornyl acetate	1290	-	0.05 (0.01)	-	-	0.07 (0.01)
28	189(8) 121(100) 93(82)	1320	0.02 (0.01)	-	0.04(0.01)	0.03 (0.01)	0.02 (0.01)
29	bicycloelemene	1341	0.54 (0.04)	0.15 (0.02)	0.89(0.03)	0.64 (0.04)	0.95 (0.04)
30	δ-elemene	1343	-	0.03 (0.01)	0.10(0.02)	0.09 (0.01)	0.05 (0.02)
31	204[M+](17) 81(100) 93(83)	1355	-	0.27 (0.03)	0.33(0.04)	0.12 (0.02)	0.03 (0.01)
32	anastreptene	1370	5.93 (0.07)	6.21 (0.07)	5.36(0.07)	4.65 (0.12)	4.01 (0.08)
33	α-funebrene	1385	0.44 (0.03)	0.08 (0.01)	0.15(0.02)	0.26 (0.02)	0.80 (0.06)
34	β-elemene	1394	0.08 (0.01)	0.47 (0.03)	0.71(0.06)	0.58 (0.02)	0.01 (0.01)
35	7-epi-sesquithujene	1408	0.13 (0.02)	0.10 (0.02)	0.05(0.01)	0.07 (0.01)	0.08 (0.01)
36	italicene	1409	0.08 (0.01)	0.12 (0.02)	0.11(0.02)	0.12 (0.01)	0.12 (0.02)
37	9-aristolene	1423	0.04 (0.01)	0.29 (0.03)	0.13(0.02)	0.16 (0.01)	0.07 (0.01)
38	1(10),8-aristoladiene	1429	3.52 (0.04)	0.08 (0.01)	3.69(0.07)	2.24 (0.04)	4.15 (0.06)
39	204[M+](6) 107(100) 79(48)	1432	0.03 (0.02)	0.13 (0.02)	0.17(0.02)	0.06 (0.02)	0.13 (0.02)
40	202[M+](4) 91(100) 185(89)	1434	-	0.13 (0.02)	0.09(0.01)	0.09 (0.01)	0.08 (0.01)
41	204[M+](24) 91(100) 105(92)	1436	0.11 (0.02)	-	0.16(0.02)	0.25 (0.01)	0.09 (0.01)
42	204[M+](18) 107(100) 161(88)	1438	0.10 (0.02)	-	0.23(0.02)	0.27 (0.01)	5.09 (0.04)
43	204[M+](9) 119(100) 91(64)	1439	-	0.12 (0.02)	0.23(0.02)	0.10 (0.01)	0.10 (0.02)
44	202[M+](30) 131(100) 159(62)	1440	6.13 (0.04)	8.80 (0.08)	5.96(0.08)	5.70 (0.08)	0.37 (0.03)
45	202[M+](24) 69(100) 41(87)	1443	0.34 (0.03)	0.02 (0.01)	0.02(0.01)	0.01 (0.01)	0.58 (0.04)
46	β-barbatene	1445	0.37 (0.03)	0.51 (0.02)	0.74(0.03)	0.93 (0.02)	0.11 (0.02)
47	202[M+](19) 91(100) 41(85)	1450	0.15 (0.02)	2.24 (0.04)	-	-	1.25 (0.05)
48	202[M+](23) 91(100) 159(93)	1452	-	1.94 (0.04)	1.44(0.03)	1.54 (0.06)	1.31 (0.04)
49	202[M+](23) 159(100) 131(74)	1455	1.81 (0.04)	0.27 (0.01)	1.63(0.03)	1.84 (0.05)	0.25 (0.02)
50	204[M+](7) 159(100) 91(97)	1457	0.12 (0.02)	0.20 (0.02)	0.27(0.02)	0.42 (0.03)	0.37 (0.03)
51	218[M+](26) 148(100) 133(75)	1466	0.02 (0.00)	0.17 (0.02)	0.67(0.03)	1.23 (0.04)	1.51 (0.06)
52	204[M+](36) 119(100)93(61)	1469	7.23 (0.08)	0.03 (0.01)	-	-	-
53	γ-curcumene	1475	6.94 (0.05)	0.20 (0.02)	0.05 (0.01)	0.04 (0.01)	0.02 (0.01)
54	218[M+](12) 105(100) 91(92)	1476	-	1.49 (0.06)	0.44 (0.02)	0.51 (0.02)	0.64 (0.02)

55	α-curcumene	1477	3.43 (0.03)	-	-	-	0.07 (0.01)
56	218[M+](25) 105(100) 91(95)	1477	-	2.45 (0.08)	1.84 (0.05)	3.00 (0.08)	1.44 (0.06)
57	α-zingiberene	1479	0.56 (0.02)	-	-	-	-
58	bicyclogermacrene	1481	3.63 (0.04)	1.96 (0.04)	6.05 (0.06)	5.65 (0.07)	5.90 (0.07)
59	202[M+](29) 91(100) 133(92)	1500	2.16 (0.05)	-	-	-	-
60	γ-bisabolene	1505	1.21 (0.03)	8.52 (0.09)	5.77 (0.05)	5.12 (0.06)	6.87 (0.06)
61	202[M+](32) 133(100) 105(69)	1510	1.94 (0.02)	-	-	-	-
62	218[M+](29) 91(100) 93(97)	1513	0.06 (0.01)	1.60 (0.06)	1.29 (0.03)	2.71 (0.04)	0.74 (0.04)
63	218[M+](11) 132(100) 105(93)	1519	0.26 (0.03)	1.48 (0.05)	1.23 (0.03)	1.55 (0.04)	1.98 (0.05)
64	β-sesquiphellandrene	1524	1.60 (0.04)	0.05 (0.01)	0.23 (0.02)	0.30 (0.02)	0.09 (0.01)
65	218[M+](3) 159(100) 131(76)	1529	1.59 (0.02)	-	-	-	-
66	218[M+](24) 148(100) 133(63)	1532	-	16.45 (0.13)	12.01 (0.12)	10.98 (0.06)	10.64 (0.06)
67	220[M+](8) 85(100) 135(89)	1545	0.30 (0.03)	0.07 (0.01)	0.59 (0.03)	0.66 (0.03)	0.08 (0.02)
68	218[M+](4) 135(100) 107(42)	1548	-	0.08 (0.02)	0.11 (0.01)	0.19 (0.02)	0.17 (0.01)
69	202[M+](85) 131(100) 91(81)	1551	0.26 (0.02)	-	-	-	-
70	218[M+](6) 91(100) 157(90)	1554	-	0.11 (0.02)	2.09 (0.05)	2.58 (0.05)	2.05 (0.05)
71	218[M+](25) 145(100) 147(97)	1561	-	0.25 (0.03)	0.24 (0.03)	0.29 (0.02)	0.19 (0.01)
72	218[M+](4) 93(100) 43(75)	1568	-	0.65 (0.04)	0.75 (0.03)	0.25 (0.02)	0.77 (0.03)
73	4,5-dehydroviridiflorol	1572	0.50 (0.03)	0.90 (0.04)	0.45 (0.02)	2.20 (0.06)	0.13 (0.01)
74	222[M+](3) 43(100) 81(53)	1578	0.34 (0.02)	3.27 (0.06)	0.05 (0.01)	0.07 (0.01)	0.30 (0.04)
75	218[M+](6) 43(100) 93(57)	1579	0.25 (0.02)	0.53 (0.04)	0.60 (0.03)	0.75 (0.03)	0.98 (0.04)
76	218[M+](7) 43(100) 91(67)	1581	-	0.62 (0.03)	1.37 (0.06)	1.53 (0.04)	0.37 (0.03)
77	220[M+](14) 79(100) 93(93)	1584	0.26 (0.01)	6.29 (0.07)	0.03 (0.01)	0.24 (0.02)	-
78	220[M+](1) 94(100) 79(43)	1589	0.96 (0.03)	-	-	-	-
79	218[M+](25) 145(100) 147(87)	1594	-	10.93 (0.09)	17.06 (0.10)	16.43 (0.12)	17.42 (0.14)
80	bisabola-2,10-diene[1,9]oxide	1596	38.31 (0.09)	3.63 (0.06)	4.97 (0.07)	2.39 (0.06)	2.17 (0.09)
81	218[M+](1) 94(100) 79(52)	1605	-	0.12 (0.01)	4.82 (0.06)	5.84 (0.07)	5.93 (0.10)
82	218[M+](26) 145(100) 43(92)	1613	-	3.26 (0.04)	8.88 (0.09)	6.98 (0.09)	0.97 (0.06)
83	220[M+](2) 94(100) 79(39)	1625	2.58 (0.03)	-	-	-	-
84	218[M+](5) 145(100) 160(48)	1641	-	0.14 (0.03)	0.22 (0.02)	0.28 (0.03)	10.64 (0.05)
85	218[M+](18) 105(100) 120(83)	1646	0.12 (0.02)	0.05 (0.01)	0.11 (0.02)	0.12 (0.02)	0.17 (0.03)
86	218[M+](5) 135(100) 107(52)	1651	-	0.90 (0.03)	0.03 (0.01)	0.07 (0.01)	0.07 (0.02)
87	220[M+](2) 91(100) 43(91)	1658	-	0.12 (0.02)	0.14 (0.02)	0.19 (0.02)	0.82 (0.03)
88	220[M+](8) 159(100) 91(81)	1668	0.23 (0.03)	-	-	-	-
89	220[M+](5) 161(100) 91(69)	1670	-	0.46 (0.04)	0.92 (0.03)	1.17 (0.05)	0.26 (0.01)
90	218[M+](38) 145(100) 91(51)	1677	-	0.02 (0.01)	0.29 (0.02)	0.65 (0.04)	0.08 (0.01)
91	218[M+](2) 179(100) 161(92)	1686	-	0.04 (0.01)	0.08 (0.01)	0.07 (0.01)	0.09 (0.01)
92	218[M+](9) 105(100) 119(59)	1689	0.19 (0.02)	0.23 (0.02)	0.09 (0.01)	0.27 (0.02)	0.06 (0.01)
93	218[M+](22) 83(100) 94(92)	1699	0.17 (0.02)	0.06 (0.01)	0.10 (0.01)	0.24 (0.02)	0.14 (0.01)
94	218[M+](28) 135(100) 91(77)	1701	0.06 (0.01)	0.07 (0.01)	0.02 (0.00)	0.03 (0.01)	0.03 (0.01)
95	218[M+](20) 91(100) 133(98)	1706	0.05 (0.01)	0.06 (0.01)	0.03 (0.01)	0.16 (0.02)	0.07 (0.01)
96	220[M+](4) 110(100) 95(62)	1708	0.14 (0.02)	0.06 (0.01)	0.05 (0.02)	0.08 (0.01)	0.06 (0.01)
97	218[M+](11) 123(100) 95(62)	1712	-	0.64 (0.03)	-	-	0.08 (0.01)
98	220[M+](18) 83(100) 125(79)	1722	0.08 (0.03)	-	0.05 (0.01)	0.12 (0.01)	0.01 (0.00)
99	218[M+](2) 120(100) 83(34)	1729	0.05 (0.02)	-	-	-	-
100	218[M+](1) 121(100) 165(59)	1739	0.05 (0.02)	-	-	-	0.05 (0.01)
101	218[M+](2) 82(100) 41(50)	1744	0.06 (0.02)	0.01 (0.00)	-	-	0.04 (0.01)
102	218[M+](3) 82(100) 41(49)	1754	0.08 (0.02)	0.10 (0.02)	0.12 (0.02)	0.16 (0.02)	0.01 (0.01)
103	220[M+](9) 137(100) 135(78)	1759	0.10 (0.03)	-	-	-	-
104	218[M+](29) 136(100) 121(81)	1762	0.09 (0.01)	-	-	-	0.11 (0.02)
105	218[M+](1) 183(100) 198(61)	1795	0.06 (0.01)	0.45 (0.04)	0.12 (0.02)	0.32 (0.04)	0.01 (0.01)
106	218[M+](17) 82(100) 109(83)	1800	0.13 (0.02)	0.01 (0.01)	-	-	-
107	221[M+](1) 82(100) 67(39)	1808	0.09 (0.01)	0.01 (0.00)	-	-	0.04 (0.01)
	Total		97.90 (1.79)	97.27 (2.40)	98.18 (2.16)	96.91 (2.26)	96.70 (2.29)
	% Identified		68.84 (0.81)	28.32 (0.84)	31.00 (0.78)	26.58 (0.76)	27.71 (0.79)
	Including:						
	Aliphatics		0.56 (0.09)	1.66 (0.15)	0.37 (0.07)	0.32 (0.07)	0.28 (0.06)

Aromatics	0.94 (0.12)	3.07 (0.13)	1.14 (0.12)	0.78 (0.10)	1.65 (0.10)
Monoterpene hydrocarbons	0.02 (0.01)	0.12 (0.04)	0.03 (0.01)	0.03 (0.02)	0.11 (0.03)
Monoterpenoide hydrocarbons	-	0.15 (0.02)	-	-	0.07 (0.01)
Sesquiterpene hydrocarbons	28.51 (0.47)	18.79 (0.40)	24.03 (0.49)	20.86 (0.45)	23.31 (0.49)
Sesquiterpenoide hydrocarbons	38.81 (0.12)	4.53 (0.10)	5.42 (0.09)	4.59 (0.12)	2.30 (0.10)

- less than 0.01%. [a] Retention index on Quadrex 007-5MS column. [b] For abbreviations of samples see Table 1, 2. ( ) standard deviation.

Table S1e. Volatile compounds detected in the samples CSU2-5 – CSU2-9.

No.	Compounds	RI [a]	Code [b]				
			CSU2-5	CSU2-6	CSU2-7	CSU2-8	CSU2-9
1	hexanal	782	0.01 (0.01)	0.01 (0.01)	0.01 (0.01)	0.01 (0.01)	0.04 (0.01)
2	3-methylbutanoic acid	817	-	-	-	-	-
3	2-methylbutanoic acid	832	-	-	-	-	-
4	3-hexen-1-ol	858	0.03 (0.01)	0.01 (0.01)	0.01 (0.01)	0.01 (0.01)	0.06 (0.01)
5	1-hexanol	867	-	-	-	-	-
6	tricyclene	927	0.03 (0.01)	0.04 (0.01)	0.06 (0.01)	0.03 (0.01)	0.04 (0.01)
7	α-pinene	936	0.01 (0.01)	0.02 (0.01)	0.01 (0.01)	0.01 (0.01)	0.02 (0.01)
8	86[M+](50) 42(100) 86(38)	957	0.03 (0.01)	0.02 (0.01)	0.02 (0.01)	0.01 (0.01)	0.04 (0.01)
9	hexanoic acid	975	-	-	-	-	-
10	β-pinene	975	0.03 (0.01)	0.09 (0.01)	0.04 (0.01)	0.03 (0.01)	0.05 (0.01)
11	1-octen-3-ol	979	0.02 (0.01)	0.03 (0.01)	0.02 (0.01)	0.02 (0.01)	0.01 (0.01)
12	3-octanone	985	0.05 (0.01)	0.06 (0.01)	0.07 (0.01)	0.05 (0.01)	0.08 (0.01)
13	3-octanol	994	0.08 (0.01)	0.09 (0.01)	0.10 (0.02)	0.17 (0.02)	0.17 (0.02)
14	benzenemethanol	1033	0.34 (0.03)	0.81 (0.02)	0.68 (0.04)	0.16 (0.02)	0.40 (0.03)
15	benzeneacetaldehyde	1043	0.11 (0.02)	0.02 (0.01)	0.05 (0.01)	0.02 (0.01)	0.04 (0.01)
16	2-ethylhexanoic acid	1108	0.05 (0.01)	0.06 (0.01)	0.04 (0.01)	0.03 (0.01)	0.08 (0.01)
17	benzeneethanol	1116	2.81 (0.06)	1.22 (0.03)	1.35 (0.06)	1.44 (0.04)	0.28 (0.02)
18	126[M+](11) 55(100) 98(84)	1154	-	-	-	-	0.04 (0.01)
19	122[M+](20) 91(100) 44(58)	1164	0.01 (0.01)	0.03 (0.01)	0.01 (0.01)	0.02 (0.01)	0.07 (0.01)
20	140[M+](4) 43(100) 57(60)	1200	0.01 (0.01)	0.01 (0.01)	0.02 (0.01)	0.01 (0.01)	0.01 (0.01)
21	128[M+](5) 44(100) 57(63)	1203	0.17 (0.02)	0.12 (0.02)	0.06 (0.01)	0.07 (0.01)	0.29 (0.03)
22	152[M+](92) 67(100) 109(98)	1217	0.15 (0.02)	0.13 (0.02)	0.25 (0.02)	0.36 (0.02)	0.39 (0.03)
23	phenoxyethanol	1223	-	-	-	-	-
24	1-phenoxy-2-propanol	1247	0.01 (0.01)	0.02 (0.01)	0.02 (0.01)	0.01 (0.01)	-
25	144[M+](38) 44(100) 129(72)	1258	-	-	-	-	0.14 (0.02)
26	bornyl acetate	1285	-	-	-	-	0.13 (0.02)
27	isobornyl acetate	1290	0.04 (0.01)	0.04 (0.01)	0.06 (0.01)	0.06 (0.01)	0.04 (0.01)
28	189(8) 121(100) 93(82)	1320	0.15 (0.02)	0.06 (0.01)	0.18 (0.02)	0.22 (0.01)	0.03 (0.01)
29	bicycloelemene	1341	0.75 (0.03)	0.70 (0.02)	1.03 (0.04)	1.04 (0.04)	0.56 (0.04)
30	δ-elemene	1343	0.01 (0.01)	0.02 (0.01)	0.03 (0.01)	0.04 (0.01)	0.03 (0.01)
31	204[M+](17) 81(100) 93(83)	1355	0.22 (0.02)	0.12 (0.02)	0.08 (0.01)	0.02 (0.01)	0.40 (0.04)
32	anastreptene	1370	4.10 (0.08)	3.87 (0.05)	4.61 (0.07)	3.06 (0.06)	5.06 (0.07)
33	α-funebrene	1385	0.68 (0.04)	0.65 (0.03)	0.82 (0.06)	0.89 (0.04)	0.83 (0.04)
34	β-elemene	1394	0.04 (0.02)	0.05 (0.01)	0.05 (0.01)	0.02 (0.01)	0.05 (0.01)
35	7-epi-sesquithujene	1408	0.16 (0.02)	0.13 (0.01)	0.13 (0.02)	0.09 (0.01)	0.09 (0.01)
36	italicene	1409	0.11 (0.02)	0.09 (0.01)	0.13 (0.02)	0.15 (0.01)	0.13 (0.01)
37	9-aristolene	1423	0.04 (0.01)	0.07 (0.01)	0.07 (0.01)	0.09 (0.01)	0.07 (0.01)
38	1(10),8-aristoladiene	1429	3.11 (0.06)	2.64 (0.11)	3.51 (0.06)	4.48 (0.05)	3.26 (0.07)
39	204[M+](6) 107(100) 79(48)	1432	0.09 (0.01)	0.11 (0.03)	0.14 (0.02)	0.13 (0.02)	0.10 (0.01)
40	202[M+](4) 91(100) 185(89)	1434	0.13 (0.01)	0.15 (0.02)	0.19 (0.01)	0.15 (0.02)	0.09 (0.01)
41	204[M+](24) 91(100) 105(92)	1436	0.22 (0.01)	0.41 (0.03)	0.08 (0.01)	0.06 (0.01)	0.07 (0.01)
42	204[M+](18) 107(100) 161(88)	1438	4.83 (0.08)	4.34 (0.09)	5.52 (0.06)	5.22 (0.04)	4.79 (0.06)
43	204[M+](9) 119(100) 91(64)	1439	0.12 (0.02)	0.11 (0.03)	0.08 (0.01)	0.06 (0.01)	0.09 (0.01)
44	202[M+](30) 131(100) 159(62)	1440	0.45 (0.02)	0.64 (0.04)	0.55 (0.03)	0.45 (0.07)	0.61 (0.02)
45	202[M+](24) 69(100) 41(87)	1443	0.43 (0.02)	0.71 (0.04)	0.85 (0.03)	0.73 (0.05)	0.33 (0.02)
46	β-barbatene	1445	0.59 (0.03)	0.14 (0.02)	0.16 (0.02)	0.12 (0.01)	0.13 (0.01)
47	202[M+](19) 91(100) 41(85)	1450	1.23 (0.09)	1.16 (0.05)	1.40 (0.04)	1.56 (0.06)	1.59 (0.04)
48	202[M+](23) 91(100) 159(93)	1452	1.20 (0.07)	1.31 (0.04)	1.48 (0.04)	1.39 (0.04)	1.28 (0.05)
49	202[M+](23) 159(100) 131(74)	1455	0.26 (0.03)	0.29 (0.02)	0.29 (0.03)	0.28 (0.02)	0.11 (0.01)
50	204[M+](7) 159(100) 91(97)	1457	0.80 (0.03)	1.55 (0.05)	1.21 (0.04)	0.64 (0.03)	0.44 (0.02)
51	218[M+](26) 148(100) 133(75)	1466	0.56 (0.04)	0.48 (0.04)	0.44 (0.03)	0.62 (0.03)	0.56 (0.04)
52	204[M+](36) 119(100)93(61)	1469	-	-	-	-	-
53	γ-curcumene	1475	0.08 (0.01)	0.04 (0.01)	0.06 (0.01)	0.05 (0.01)	0.04 (0.01)
54	218[M+](12) 105(100) 91(92)	1476	1.75 (0.05)	2.39 (0.05)	2.18 (0.08)	1.55 (0.03)	1.09 (0.07)

55	α-curcumene	1477	0.09 (0.01)	0.16 (0.02)	0.14 (0.02)	0.08 (0.01)	0.22 (0.02)
56	218[M+](25) 105(100) 91(95)	1477	5.20 (0.06)	4.38 (0.06)	5.91 (0.07)	6.08 (0.08)	4.08 (0.05)
57	α-zingiberene	1479	-	-	-	-	-
58	bicyclogermacrene	1481	1.03 (0.03)	1.23 (0.04)	1.36 (0.04)	1.25 (0.04)	1.16 (0.04)
59	202[M+](29) 91(100) 133(92)	1500	-	-	-	-	-
60	γ-bisabolene	1505	5.77 (0.07)	4.17 (0.05)	4.23 (0.04)	6.56 (0.05)	6.60 (0.06)
61	202[M+](32) 133(100) 105(69)	1510	-	-	-	-	-
62	218[M+](29) 91(100) 93(97)	1513	1.45 (0.03)	2.03 (0.04)	1.57 (0.04)	1.02 (0.05)	0.61 (0.04)
63	218[M+](11) 132(100) 105(93)	1519	1.92 (0.04)	1.72 (0.04)	2.18 (0.05)	2.22 (0.06)	1.50 (0.05)
64	β-sesquiphellandrene	1524	0.17 (0.02)	0.43 (0.03)	0.26 (0.02)	0.14 (0.01)	0.06 (0.01)
65	218[M+](3) 159(100) 131(76)	1529	-	-	-	-	-
66	218[M+](24) 148(100) 133(63)	1532	10.61 (0.09)	11.42 (0.14)	11.32 (0.15)	9.69 (0.09)	10.78 (0.05)
67	220[M+](8) 85(100) 135(89)	1545	0.14 (0.02)	0.08(0.01)	0.06 (0.02)	0.08 (0.01)	0.02 (0.01)
68	218[M+](4) 135(100) 107(42)	1548	0.34 (0.03)	0.45 (0.03)	0.28 (0.02)	0.25 (0.02)	0.09 (0.02)
69	202[M+](85) 131(100) 91(81)	1551	-	-	-	-	-
70	218[M+](6) 91(100) 157(90)	1554	2.38 (0.03)	2.77 (0.03)	2.57 (0.04)	1.96 (0.09)	1.84 (0.05)
71	218[M+](25) 145(100) 147(97)	1561	0.26 (0.04)	0.23 (0.02)	0.13 (0.01)	0.21 (0.02)	0.14 (0.02)
72	218[M+](4) 93(100) 43(75)	1568	0.90 (0.05)	0.55 (0.02)	0.67 (0.02)	0.15 (0.02)	0.57 (0.03)
73	4,5-dehydroviridiflorol	1572	0.16 (0.03)	1.39 (0.04)	0.69 (0.02)	0.25 (0.02)	0.26 (0.01)
74	222[M+](3) 43(100) 81(53)	1578	0.50 (0.03)	0.52 (0.03)	0.52 (0.02)	0.44 (0.02)	0.70 (0.02)
75	218[M+](6) 43(100) 93(57)	1579	1.34 (0.04)	1.68 (0.03)	1.35 (0.03)	1.11 (0.04)	0.99 (0.03)
76	218[M+](7) 43(100) 91(67)	1581	0.80 (0.03)	0.72 (0.03)	0.45 (0.02)	0.28 (0.02)	0.18 (0.02)
77	220[M+](14) 79(100) 93(93)	1584	-	-	-	-	-
78	220[M+](1) 94(100) 79(43)	1589	-	-	-	-	-
79	218[M+](25) 145(100) 147(87)	1594	16.95 (0.14)	17.01 (0.14)	16.56 (0.10)	16.40 (0.13)	19.84 (0.11)
80	bisabola-2,10-diene[1,9]oxide	1596	4.13 (0.09)	1.73 (0.04)	2.12 (0.06)	3.54 (0.08)	4.32 (0.08)
81	218[M+](1) 94(100) 79(52)	1605	3.11 (0.08)	1.54 (0.03)	2.35 (0.05)	2.12 (0.05)	7.03 (0.07)
82	218[M+](26) 145(100) 43(92)	1613	2.26 (0.07)	2.86 (0.04)	1.87 (0.04)	1.80 (0.06)	0.98 (0.03)
83	220[M+](2) 94(100) 79(39)	1625	-	-	-	-	-
84	218[M+](5) 145(100) 160(48)	1641	9.34 (0.06)	11.28 (0.08)	11.36 (0.11)	13.51 (0.13)	10.23 (0.09)
85	218[M+](18) 105(100) 120(83)	1646	0.21 (0.02)	0.32(0.03)	0.22 (0.02)	0.21 (0.04)	-
86	218[M+](5) 135(100) 107(52)	1651	0.14 (0.02)	0.07 (0.01)	0.03 (0.01)	0.05 (0.01)	0.04 (0.01)
87	220[M+](2) 91(100) 43(91)	1658	1.29 (0.05)	1.42 (0.04)	0.70 (0.02)	0.58 (0.02)	0.53 (0.03)
88	220[M+](8) 159(100) 91(81)	1668	-	-	-	-	-
89	220[M+](5) 161(100) 91(69)	1670	0.36 (0.04)	0.59 (0.03)	0.45 (0.02)	0.27 (0.03)	0.19 (0.02)
90	218[M+](38) 145(100) 91(51)	1677	0.21 (0.02)	0.26 (0.03)	0.05 (0.01)	0.07 (0.01)	0.06 (0.01)
91	218[M+](2) 179(100) 161(92)	1686	0.11 (0.01)	0.15 (0.02)	0.09 (0.01)	0.08 (0.01)	0.11 (0.01)
92	218[M+](9) 105(100) 119(59)	1689	0.14 (0.02)	0.08 (0.01)	0.05 (0.01)	0.02 (0.01)	-
93	218[M+](22) 83(100) 94(92)	1699	0.12 (0.02)	0.18 (0.01)	0.13 (0.02)	0.15 (0.03)	0.08 (0.01)
94	218[M+](28) 135(100) 91(77)	1701	0.04 (0.01)	0.08 (0.01)	0.05 (0.01)	0.04 (0.01)	0.03 (0.01)
95	218[M+](20) 91(100) 133(98)	1706	0.03 (0.01)	0.04 (0.01)	0.03 (0.01)	0.06 (0.01)	0.03 (0.01)
96	220[M+](4) 110(100) 95(62)	1708	0.05 (0.01)	0.08 (0.01)	0.02 (0.01)	0.05 (0.01)	-
97	218[M+](11) 123(100) 95(62)	1712	0.05 (0.01)	0.02 (0.01)	0.08 (0.01)	0.06 (0.01)	0.03 (0.01)
98	220[M+](18) 83(100) 125(79)	1722	0.01 (0.00)	0.02 (0.00)	0.02 (0.01)	0.02 (0.01)	0.03 (0.01)
99	218[M+](2) 120(100) 83(34)	1729	-	-	-	-	-
100	218[M+](1) 121(100) 165(59)	1739	0.06 (0.01)	0.11 (0.01)	0.04 (0.01)	0.11 (0.02)	0.02 (0.01)
101	218[M+](2) 82(100) 41(50)	1744	0.05 (0.01)	0.05 (0.01)	0.01 (0.01)	0.03 (0.01)	-
102	218[M+](3) 82(100) 41(49)	1754	0.01 (0.01)	0.02 (0.01)	0.01 (0.00)	0.01 (0.01)	0.03 (0.01)
103	220[M+](9) 137(100) 135(78)	1759	-	0.01 (0.01)	-	-	-
104	218[M+](29) 136(100) 121(81)	1762	0.13 (0.02)	0.16 (0.01)	0.14 (0.01)	0.11 (0.02)	0.12 (0.02)
105	218[M+](1) 183(100) 198(61)	1795	0.04 (0.01)	0.04 (0.01)	0.03 (0.01)	0.04 (0.01)	-
106	218[M+](17) 82(100) 109(83)	1800	-	-	-	-	-
107	221[M+](1) 82(100) 67(39)	1808	0.20 (0.02)	0.10 (0.01)	0.09 (0.01)	0.08 (0.01)	0.01 (0.01)
	Total		98.23 (2.55)	97.25 (2.37)	98.37 (2.29)	96.84 (2.35)	97.80 (2.11)
	% Identified		24.64 (0.80)	20.03 (0.68)	21.93 (0.76))	23.91 (0.66)	24.32 (0.70)
	Including:						
	Aliphatics		0.24 (0.06)	0.26 (0.06)	0.25 (0.07)	0.29 (0.07)	0.44 (0.07)

Aromatics	3.27 (0.12)	2.07 (0.07)	2.10 (0.12)	1.63 (0.08)	0.72 (0.06)
Monoterpene hydrocarbons	0.07 (0.03)	0.15 (0.03)	0.11 (0.03)	0.07 (0.03)	0.11 (0.03)
Monoterpenoide hydrocarbons	0.04 (0.01)	0.04 (0.01)	0.06 (0.01)	0.06 (0.01)	0.17 (0.03)
Sesquiterpene hydrocarbons	16.73 (0.46)	14.39 (0.43)	16.59 (0.45)	18.06 (0.37)	18.30 (0.42)
Sesquiterpenoide hydrocarbons	4.29 (0.12)	3.12 (0.08)	2.81 (0.08)	3.79 (0.10)	4.58 (0.09)

- less than 0.01%. [a] Retention index on Quadrex 007-5MS column. [b] For abbreviations of samples see Table 2. ( ) standard deviation.

Table S2a. Volatile compounds detected in the samples CSU1-17 – CSU1-21.

No.	Compounds	RI [a]	Code [b]				
			CSU1-17	CSU1-18	CSU1-19	CSU1-20	CSU1-21
1	hexanal	782	0.08 (0.01)	0.05 (0.01)	0.10 (0.01)	0.10 (0.01)	-
2	3-methylbutanoic acid	817	0.10 (0.01)	0.09 (0.01)	0.12 (0.02)	-	-
3	2-methylbutanoic acid	832	0.03 (0.01)	0.15 (0.02)	0.05 (0.01)	-	-
4	3-hexen-1-ol	858	-	-	-	-	0.03 (0.00)
5	1-hexanol	867	0.28 (0.02)	0.49 (0.03)	0.78 (0.04)	0.15 (0.02)	-
6	tricyclene	927	-	-	-	-	0.02 (0.00)
7	α-pinene	936	0.03 (0.00)	0.02 (0.00)	0.02 (0.00)	0.05 (0.01)	0.01 (0.00)
8	86[M+](50) 42(100) 86(38)	957	0.04 (0.01)	0.18 (0.01)	0.10 (0.01)	0.06 (0.01)	0.01 (0.00)
9	hexanoic acid	975	0.15 (0.03)	0.13 (0.02)	0.15 (0.01)	0.05 (0.01)	0.03 (0.00)
10	β-pinene	975	0.01 (0.00)	0.02 (0.00)	0.01 (0.00)	0.02 (0.00)	0.01 (0.00)
11	1-octen-3-ol	979	0.10 (0.02)	0.25 (0.01)	0.09 (0.01)	0.10 (0.02)	0.05 (0.02)
12	3-octanone	985	0.06 (0.01)	0.10 (0.01)	0.05 (0.01)	0.09 (0.01)	0.06 (0.01)
13	3-octanol	994	-	-	-	-	0.02 (0.00)
14	benzenemethanol	1033	1.38 (0.03)	1.36 (0.03)	1.05 (0.04)	0.65 (0.04)	0.28 (0.04)
15	benzeneacetaldehyde	1043	0.42 (0.02)	0.41 (0.02)	0.35 (0.02)	0.22 (0.02)	0.03 (0.00)
16	2-ethylhexanoic acid	1108	0.11 (0.01)	0.22 (0.01)	0.11 (0.01)	0.05 (0.01)	0.02 (0.00)
17	benzeneethanol	1116	1.02 (0.05)	1.13 (0.05)	0.87 (0.02)	0.38 (0.02)	0.52 (0.04)
18	126[M+](11) 55(100) 98(84)	1154	0.09 (0.01)	0.11 (0.01)	0.06 (0.01)	0.07 (0.01)	-
19	122[M+](20) 91(100) 44(58)	1164	-	-	-	-	-
20	140[M+](4) 43(100) 57(60)	1200	-	-	-	-	0.04 (0.01)
21	128[M+](5) 44(100) 57(63)	1203	0.01 (0.00)	0.03 (0.01)	0.02 (0.00)	0.05 (0.01)	0.32 (0.03)
22	152[M+](92) 67(100) 109(98)	1217	0.14 (0.02)	0.23 (0.01)	0.22 (0.01)	0.09 (0.01)	0.24 (0.02)
23	phenoxyethanol	1223	2.53 (0.06)	1.47 (0.04)	1.69 (0.03)	0.42 (0.04)	0.48 (0.05)
24	1-phenoxy-2-propanol	1247	0.07 (0.01)	0.05 (0.01)	0.08 (0.01)	-	-
25	144[M+](38) 44(100) 129(72)	1258	0.02 (0.01)	0.03 (0.01)	0.03 (0.01)	0.01 (0.00)	-
26	bornyl acetate	1285	0.01 (0.00)	0.02 (0.00)	0.05 (0.00)	-	-
27	isobornyl acetate	1290	0.03 (0.00)	0.03 (0.00)	0.03 (0.00)	-	0.04 (0.01)
28	189(8) 121(100) 93(82)	1320	-	-	-	-	0.02 (0.00)
29	bicycloelemene	1341	0.32 (0.02)	0.11 (0.02)	0.31 (0.02)	0.15 (0.02)	1.02 (0.03)
30	δ-elemene	1343	-	-	-	-	0.04 (0.00)
31	204[M+](17) 81(100) 93(83)	1355	-	-	-	-	-
32	anastreptene	1370	6.98 (0.05)	6.42 (0.03)	7.71 (0.05)	4.68 (0.05)	9.53 (0.05)
33	α-funebrene	1385	0.39 (0.02)	0.55 (0.02)	0.42 (0.02)	0.23 (0.01)	0.21 (0.02)
34	β-elemene	1394	0.09 (0.01)	0.06 (0.01)	0.11 (0.01)	0.09 (0.01)	0.04 (0.01)
35	7-epi-sesquithujene	1408	0.29 (0.03)	0.42 (0.02)	0.09 (0.02)	0.11 (0.01)	0.11 (0.01)
36	italicene	1409	0.02 (0.01)	0.09 (0.01)	0.10 (0.01)	0.12 (0.01)	0.16 (0.02)
37	9-aristolene	1423	0.04 (0.00)	0.06 (0.00)	0.05 (0.00)	0.07 (0.01)	0.04 (0.01)
38	1(10),8-aristoladiene	1429	3.02 (0.04)	4.72 (0.05)	5.02 (0.04)	1.96 (0.04)	6.11 (0.06)
39	204[M+](6) 107(100) 79(48)	1432	0.07 (0.01)	0.08 (0.01)	0.07 (0.01)	0.01 (0.00)	0.34 (0.03)
40	202[M+](4) 91(100) 185(89)	1434	-	-	-	-	-
41	204[M+](24) 91(100) 105(92)	1436	0.09 (0.01)	0.12 (0.01)	0.11 (0.01)	0.09 (0.01)	0.29 (0.01)
42	204[M+](18) 107(100) 161(88)	1438	0.06 (0.01)	0.08 (0.01)	0.06 (0.01)	0.14 (0.01)	8.92 (0.05)
43	204[M+](9) 119(100) 91(64)	1439	-	-	-	-	-
44	202[M+](30) 131(100) 159(62)	1440	6.78 (0.05)	6.23 (0.04)	7.49 (0.06)	5.62 (0.05)	0.64 (0.03)
45	202[M+](24) 69(100) 41(87)	1443	0.12 (0.01)	0.32 (0.01)	0.29 (0.01)	0.21 (0.01)	1.42 (0.05)
46	β-barbatene	1445	0.22 (0.02)	0.42 (0.01)	0.62 (0.02)	0.29 (0.02)	0.15 (0.01)
47	202[M+](19) 91(100) 41(85)	1450	0.12 (0.01)	0.22 (0.01)	0.31 (0.01)	0.15 (0.02)	0.53 (0.03)
48	202[M+](23) 91(100) 159(93)	1452	-	-	-	-	-
49	202[M+](23) 159(100) 131(74)	1455	2.12 (0.04)	2.15 (0.03)	2.53 (0.04)	1.75 (0.02)	3.72 (0.04)
50	204[M+](7) 159(100) 91(97)	1457	0.11 (0.02)	0.12 (0.02)	0.16 (0.02)	0.13 (0.01)	5.96 (0.05)
51	218[M+](26) 148(100) 133(75)	1466	-	-	-	-	-
52	204[M+](36) 119(100)93(61)	1469	5.96 (0.04)	5.68 (0.04)	3.53 (0.04)	5.64 (0.06)	3.24 (0.04)
53	γ-curcumene	1475	5.83 (0.05)	5.78 (0.03)	4.42 (0.05)	5.22 (0.04)	1.34 (0.02)
54	218[M+](12) 105(100) 91(92)	1476	-	-	-	-	-

55	α-curcumene	1477	2.54 (0.04)	2.94 (0.03)	0.53 (0.03)	1.75 (0.03)	4.46 (0.02)
56	218[M+](25) 105(100) 91(95)	1477	-	-	-	-	-
57	α-zingiberene	1479	0.35 (0.02)	0.65 (0.02)	1.25 (0.02)	0.12 (0.01)	0.33 (0.01)
58	bicyclogermacrene	1481	2.65 (0.03)	1.14 (0.04)	2.76 (0.05)	1.72 (0.02)	5.47 (0.04)
59	202[M+](29) 91(100) 133(92)	1500	0.19 (0.02)	0.31 (0.01)	0.34 (0.02)	0.28 (0.01)	2.03 (0.02)
60	γ-bisabolene	1505	0.63 (0.03)	0.95 (0.02)	0.96 (0.03)	0.48 (0.02)	2.13 (0.04)
61	202[M+](32) 133(100) 105(69)	1510	1.86 (0.05)	1.48 (0.03)	1.54 (0.04)	0.91 (0.02)	0.53 (0.02)
62	218[M+](29) 91(100) 93(97)	1513	-	-	-	-	0.03 (0.01)
63	218[M+](11) 132(100) 105(93)	1519	0.16 (0.01)	0.18 (0.01)	0.26 (0.01)	0.17 (0.01)	0.63 (0.02)
64	β-sesquiphellandrene	1524	1.52 (0.04)	1.49 (0.04)	1.13 (0.03)	0.86 (0.02)	2.05 (0.04)
65	218[M+](3) 159(100) 131(76)	1529	0.95 (0.02)	2.83 (0.03)	3.46 (0.06)	0.89 (0.02)	0.43 (0.02)
66	218[M+](24) 148(100) 133(63)	1532	-	-	-	-	-
67	220[M+](8) 85(100) 135(89)	1545	0.47 (0.03)	0.38 (0.02)	0.22 (0.03)	0.64 (0.03)	0.14 (0.01)
68	218[M+](4) 135(100) 107(42)	1548	-	-	-	-	-
69	202[M+](85) 131(100) 91(81)	1551	0.15 (0.02)	0.12 (0.01)	0.10 (0.01)	0.32 (0.02)	0.45 (0.02)
70	218[M+](6) 91(100) 157(90)	1554	-	-	-	-	-
71	218[M+](25) 145(100) 147(97)	1561	-	-	-	-	-
72	218[M+](4) 93(100) 43(75)	1568	-	-	-	-	-
73	4,5-dehydroviridiflorol	1572	2.23 (0.04)	1.58 (0.03)	2.06 (0.04)	0.37 (0.02)	0.19 (0.01)
74	222[M+](3) 43(100) 81(53)	1578	1.16 (0.04)	0.98 (0.03)	1.68 (0.03)	0.07 (0.01)	0.27 (0.02)
75	218[M+](6) 43(100) 93(57)	1579	0.12 (0.01)	0.25 (0.01)	0.43 (0.02)	0.23 (0.02)	1.23 (0.03)
76	218[M+](7) 43(100) 91(67)	1581	-	-	-	-	-
77	220[M+](14) 79(100) 93(93)	1584	0.12 (0.01)	0.43 (0.02)	0.15 (0.01)	0.53 (0.04)	0.64 (0.04)
78	220[M+](1) 94(100) 79(43)	1589	0.96 (0.02)	1.47 (0.03)	1.49 (0.04)	0.21 (0.02)	0.23 (0.02)
79	218[M+](25) 145(100) 147(87)	1594	-	-	-	-	-
80	bisabola-2,10-diene[1,9]oxide	1596	35.99 (0.07)	36.23 (0.07)	34.99 (0.05)	47.87 (0.07)	27.03 (0.06)
81	218[M+](1) 94(100) 79(52)	1605	-	-	-	-	-
82	218[M+](26) 145(100) 43(92)	1613	-	-	-	-	-
83	220[M+](2) 94(100) 79(39)	1625	2.23 (0.04)	2.75 (0.03)	2.43 (0.04)	4.22 (0.03)	2.48 (0.04)
84	218[M+](5) 145(100) 160(48)	1641	-	0.01 (0.00)	-	-	-
85	218[M+](18) 105(100) 120(83)	1646	0.27 (0.01)	0.17 (0.01)	0.52 (0.02)	0.34 (0.04)	0.03 (0.01)
86	218[M+](5) 135(100) 107(52)	1651	-	-	-	-	-
87	220[M+](2) 91(100) 43(91)	1658	-	-	-	-	-
88	220[M+](8) 159(100) 91(81)	1668	0.31 (0.02)	0.18 (0.03)	0.33 (0.02)	0.54 (0.03)	0.08 (0.01)
89	220[M+](5) 161(100) 91(69)	1670	-	-	-	-	-
90	218[M+](38) 145(100) 91(51)	1677	-	-	-	-	-
91	218[M+](2) 179(100) 161(92)	1686	-	-	-	-	-
92	218[M+](9) 105(100) 119(59)	1689	0.09 (0.01)	0.15 (0.01)	0.14 (0.01)	0.47 (0.04)	0.15 (0.02)
93	218[M+](22) 83(100) 94(92)	1699	0.14 (0.01)	0.17 (0.02)	0.21 (0.02)	0.26 (0.03)	0.03 (0.00)
94	218[M+](28) 135(100) 91(77)	1701	-	-	-	0.11 (0.01)	-
95	218[M+](20) 91(100) 133(98)	1706	0.16 (0.01)	0.14 (0.01)	0.12 (0.01)	0.02 (0.00)	0.14 (0.02)
96	220[M+](4) 110(100) 95(62)	1708	-	-	-	0.33 (0.01)	-
97	218[M+](11) 123(100) 95(62)	1712	0.09 (0.01)	0.09 (0.01)	0.08 (0.01)	0.02 (0.00)	0.09 (0.02)
98	220[M+](18) 83(100) 125(79)	1722	0.21 (0.01)	0.16 (0.01)	0.09 (0.01)	1.53 (0.03)	0.06 (0.01)
99	218[M+](2) 120(100) 83(34)	1729	0.17 (0.01)	0.11 (0.01)	0.15 (0.02)	0.15 (0.02)	0.06 (0.01)
100	218[M+](1) 121(100) 165(59)	1739	0.11 (0.01)	0.10 (0.01)	0.12 (0.01)	0.09 (0.01)	0.05 (0.01)
101	218[M+](2) 82(100) 41(50)	1744	0.09 (0.01)	0.06 (0.01)	0.06 (0.01)	0.17 (0.01)	0.09 (0.02)
102	218[M+](3) 82(100) 41(49)	1754	0.14 (0.01)	0.13 (0.01)	0.03 (0.01)	0.18 (0.02)	0.11 (0.02)
103	220[M+](9) 137(100) 135(78)	1759	0.13 (0.01)	0.14 (0.02)	0.12 (0.01)	0.29 (0.03)	0.04 (0.01)
104	218[M+](29) 136(100) 121(81)	1762	0.09 (0.01)	0.19 (0.01)	0.04 (0.00)	0.35 (0.03)	0.15 (0.02)
105	218[M+](1) 183(100) 198(61)	1795	0.07 (0.01)	0.10 (0.01)	0.09 (0.01)	0.37 (0.03)	0.12 (0.02)
106	218[M+](17) 82(100) 109(83)	1800	0.15 (0.01)	0.14 (0.01)	0.14 (0.01)	0.18 (0.02)	0.09 (0.01)
107	221[M+](1) 82(100) 67(39)	1808	0.10 (0.01)	0.13 (0.01)	0.09 (0.01)	0.10 (0.01)	-
	Total		95.94 (1.50)	98.53 (1.38)	97.54 (1.48)	96.36 (1.45)	98.08 (1.50)
	% Identified		69.52 (0.81)	69.60 (0.72)	68.13 (0.73)	68.37 (0.62)	62.01 (0.63)
	Including:						
	Aliphatics		0.91 (0.12)	1.48 (0.12)	1.45 ().12)	0.54 (0.08)	0.21 (0.03)

Aromatics	5.42 (0.17)	4.42 (0.15)	4.04 (0.12)	1.67 (0.12)	1.31 (0.13)
Monoterpene hydrocarbons	0.04 (0.00)	0.04 (0.00)	0.03 (0.00)	0.07 (0.01)	0.04 (0.00)
Monoterpenoide hydrocarbons	0.04 (0.00)	0.05 (0.00)	0.08 (0.00)	-	0.04 (0.01)
Sesquiterpene hydrocarbons	24.89 (0.41)	25.80 (0.35)	25.48 (0.40)	17.85 (0.32)	33.19 (0.39)
Sesquiterpenoide hydrocarbons	38.22 (0.11)	37.81 (0.10)	37.05 (0.09)	48.24 (0.09)	27.22 (0.07)

- less than 0.01%. [a] Retention index on Quadrex 007-5MS column. [b] For abbreviations of samples see Table S1. ( ) standard deviation.

Table S2b. Volatile compounds detected in the samples CSU1-22 – CSU1-26.

No.	Compounds	RI [a]	Code [b]				
			CSU1-22	CSU1-23	CSU1-24	CSU1-25	CSU1-26
1	hexanal	782	-	-	0.03 (0.01)	0.03 (0.01)	0.04 (0.01)
2	3-methylbutanoic acid	817	-	-	0.01 (0.01)	0.02 (0.01)	0.02 (0.00)
3	2-methylbutanoic acid	832	-	-	-	-	-
4	3-hexen-1-ol	858	0.02 (0.01)	0.03 (0.01)	-	-	-
5	1-hexanol	867	-	-	0.11 (0.03)	0.13 (0.02)	0.08 (0.03)
6	tricyclene	927	0.01 (0.00)	0.02 (0.01)	-	-	-
7	α-pinene	936	0.02 (0.01)	0.01 (0.00)	0.02 (0.01)	0.01 (0.01)	0.01 (0.00)
8	86[M+](50) 42(100) 86(38)	957	0.01 (0.00)	0.01 (0.00)	0.02 (0.01)	0.03 (0.01)	0.01 (0.00)
9	hexanoic acid	975	0.01 (0.01)	0.02 (0.01)	0.08 (0.01)	0.06 (0.01)	0.05 (0.01)
10	β-pinene	975	0.01 (0.00)	0.01 (0.00)	0.01 (0.01)	0.03 (0.01)	0.02 (0.01)
11	1-octen-3-ol	979	0.06 (0.02)	0.03 (0.01)	0.08 (0.02)	0.07 (0.02)	0.05 (0.02)
12	3-octanone	985	0.09 (0.01)	0.01 (0.00)	0.11 (0.01)	0.08 (0.01)	0.06 (0.01)
13	3-octanol	994	0.01 (0.00)	0.01 (0.00)	0.02 (0.01)	0.01 (0.01)	0.02 (0.00)
14	benzenemethanol	1033	0.18 (0.02)	0.15 (0.02)	0.05 (0.01)	0.06 (0.01)	0.09 (0.01)
15	benzeneacetaldehyde	1043	0.05 (0.01)	0.09 (0.00)	0.18 (0.03)	0.22 (0.03)	0.15 (0.03)
16	2-ethylhexanoic acid	1108	0.01 (0.00)	0.02 (0.00)	0.06 (0.01)	0.07 (0.01)	0.05 (0.01)
17	benzeneethanol	1116	0.36 (0.02)	0.99 (0.04)	0.38 (0.03)	0.47 (0.04)	0.62 (0.04)
18	126[M+](11) 55(100) 98(84)	1154	-	-	0.07 (0.01)	0.08 (0.01)	0.10 (0.01)
19	122[M+](20) 91(100) 44(58)	1164	-	-	-	-	-
20	140[M+](4) 43(100) 57(60)	1200	0.05 (0.01)	0.02 (0.01)	0.01 (0.01)	0.02 (0.01)	0.01 (0.00)
21	128[M+](5) 44(100) 57(63)	1203	0.38 (0.03)	0.37 (0.02)	0.03 (0.01)	0.04 (0.01)	0.02 (0.00)
22	152[M+](92) 67(100) 109(98)	1217	0.11 (0.01)	0.24 (0.03)	0.72 (0.06)	0.24 (0.06)	0.29 (0.06)
23	phenoxyethanol	1223	0.35 (0.03)	0.21 (0.06)	0.26 (0.03)	0.38 (0.03)	0.47 (0.03)
24	1-phenoxy-2-propanol	1247	-	-	0.04 (0.01)	0.08 (0.01)	0.03 (0.01)
25	144[M+](38) 44(100) 129(72)	1258	-	-	0.01 (0.01)	0.04 (0.01)	0.02 (0.00)
26	bornyl acetate	1285	-	-	-	-	-
27	isobornyl acetate	1290	0.08 (0.01)	0.06 (0.01)	-	-	-
28	189(8) 121(100) 93(82)	1320	0.03 (0.01)	0.01 (0.00)	0.04 (0.01)	0.05 (0.01)	0.03 (0.01)
29	bicycloelemene	1341	0.96 (0.03)	1.49 (0.03)	0.95 (0.04)	0.68 (0.04)	1.43 (0.04)
30	δ-elemene	1343	0.06 (0.00)	0.01 (0.00)	-	-	-
31	204[M+](17) 81(100) 93(83)	1355	-	-	-	-	-
32	anastreptene	1370	7.12 (0.06)	8.59 (0.05)	7.45 (0.06)	7.52 (0.07)	6.89 (0.07)
33	α-funebrene	1385	0.27 (0.02)	0.32 (0.02)	0.51 (0.03)	0.56 (0.03)	0.47 (0.03)
34	β-elemene	1394	0.05 (0.01)	0.02 (0.01)	0.15 (0.01)	0.11 (0.01)	0.13 (0.01)
35	7-epi-sesquithujene	1408	0.11 (0.01)	0.08 (0.01)	0.11 (0.02)	0.43 (0.02)	0.27 (0.02)
36	italicene	1409	0.09 (0.01)	0.12 (0.01)	0.05 (0.01)	0.06 (0.01)	0.11 (0.01)
37	9-aristolene	1423	0.08 (0.02)	0.19 (0.02)	0.08 (0.01)	0.09 (0.01)	0.04 (0.01)
38	1(10),8-aristoladiene	1429	5.63 (0.06)	5.47 (0.06)	4.02 (0.06)	3.64 (0.04)	3.89 (0.04)
39	204[M+](6) 107(100) 79(48)	1432	0.24 (0.02)	0.38 (0.02)	0.06 (0.02)	0.05 (0.02)	0.07 (0.02)
40	202[M+](4) 91(100) 185(89)	1434	-	-	-	-	-
41	204[M+](24) 91(100) 105(92)	1436	0.26 (0.01)	0.29 (0.01)	0.18 (0.03)	0.25 (0.02)	0.16 (0.02)
42	204[M+](18) 107(100) 161(88)	1438	7.41 (0.05)	8.42 (0.05)	0.22 (0.03)	0.19 (0.02)	0.16 (0.02)
43	204[M+](9) 119(100) 91(64)	1439	-	-	-	-	-
44	202[M+](30) 131(100) 159(62)	1440	0.51 (0.03)	0.69 (0.02)	6.97 (0.06)	7.94 (0.04)	8.03 (0.04)
45	202[M+](24) 69(100) 41(87)	1443	0.97 (0.03)	1.48 (0.03)	0.48 (0.03)	0.49 (0.03)	0.23 (0.03)
46	β-barbatene	1445	0.16 (0.01)	0.09 (0.01)	0.87 (0.04)	0.93 (0.03)	0.28 (0.03)
47	202[M+](19) 91(100) 41(85)	1450	0.38 (0.02)	0.64 (0.02)	0.13 (0.02)	0.24 (0.02)	0.13 (0.02)
48	202[M+](23) 91(100) 159(93)	1452	-	-	-	-	-
49	202[M+](23) 159(100) 131(74)	1455	2.29 (0.04)	3.21 (0.03)	1.46 (0.06)	2.79 (0.04)	2.18 (0.04)
50	204[M+](7) 159(100) 91(97)	1457	8.53 (0.06)	6.57 (0.07)	0.23 (0.02)	0.12 (0.02)	0.14 (0.02)
51	218[M+](26) 148(100) 133(75)	1466	-	-	-	-	-
52	204[M+](36) 119(100)93(61)	1469	2.57 (0.04)	6.37 (0.05)	6.42 (0.09)	7.98 (0.08)	6.27 (0.08)
53	γ-curcumene	1475	1.69 (0.02)	1.68 (0.03)	4.97 (0.06)	7.34 (0.05)	5.43 (0.05)
54	218[M+](12) 105(100) 91(92)	1476	-	-	-	-	-

55	α-curcumene	1477	4.96 (0.03)	1.46 (0.02)	3.38 (0.04)	3.67 (0.03)	2.32 (0.03)
56	218[M+](25) 105(100) 91(95)	1477	-	-	-	-	-
57	α-zingiberene	1479	0.42 (0.01)	0.29 (0.01)	0.96 (0.02)	1.18 (0.02)	0.43 (0.02)
58	bicyclogermacrene	1481	6.31 (0.05)	3.71 (0.03)	3.41 (0.05)	3.97 (0.04)	2.14 (0.04)
59	202[M+](29) 91(100) 133(92)	1500	2.38 (0.04)	1.24 (0.02)	4.02 (0.05)	2.78 (0.05)	3.05 (0.05)
60	γ-bisabolene	1505	3.34 (0.03)	3.46 (0.03)	1.38 (0.03)	1.28 (0.03)	1.15 (0.03)
61	202[M+](32) 133(100) 105(69)	1510	0.54 (0.04)	1.59 (0.03)	2.04 (0.04)	2.31 (0.02)	1.53 (0.02)
62	218[M+](29) 91(100) 93(97)	1513	-	-	0.06 (0.01)	0.04 (0.01)	0.03 (0.01)
63	218[M+](11) 132(100) 105(93)	1519	0.69 (0.03)	1.18 (0.03)	0.33 (0.03)	0.18 (0.03)	0.14 (0.03)
64	β-sesquiphellandrene	1524	1.37 (0.03)	1.69 (0.03)	1.34 (0.03)	1.53 (0.04)	1.03 (0.04)
65	218[M+](3) 159(100) 131(76)	1529	0.32 (0.02)	0.24 (0.02)	0.87 (0.02)	1.27 (0.02)	1.23 (0.02)
66	218[M+](24) 148(100) 133(63)	1532	-	-	-	-	-
67	220[M+](8) 85(100) 135(89)	1545	0.07 (0.01)	0.13 (0.01)	0.43 (0.03)	0.47 (0.03)	0.43 (0.03)
68	218[M+](4) 135(100) 107(42)	1548	-	-	-	-	-
69	202[M+](85) 131(100) 91(81)	1551	0.37 (0.03)	0.22 (0.02)	0.32 (0.02)	0.21 (0.02)	0.16 (0.02)
70	218[M+](6) 91(100) 157(90)	1554	-	-	-	-	-
71	218[M+](25) 145(100) 147(97)	1561	-	-	-	-	-
72	218[M+](4) 93(100) 43(75)	1568	-	-	-	-	-
73	4,5-dehydroviridiflorol	1572	0.01 (0.01)	0.04 (0.01)	0.53 (0.03)	0.64 (0.03)	0.71 (0.03)
74	222[M+](3) 43(100) 81(53)	1578	0.11 (0.01)	0.44 (0.01)	0.09 (0.02)	0.11 (0.02)	0.08 (0.02)
75	218[M+](6) 43(100) 93(57)	1579	0.39 (0.03)	0.16 (0.03)	0.12 (0.02)	0.13 (0.02)	0.09 (0.02)
76	218[M+](7) 43(100) 91(67)	1581	-	-	-	-	-
77	220[M+](14) 79(100) 93(93)	1584	1.08 (0.03)	0.82 (0.03)	0.16 (0.01)	0.23 (0.02)	0.18 (0.01)
78	220[M+](1) 94(100) 79(43)	1589	0.19 (0.02)	0.18 (0.02)	1.56 (0.04)	1.23 (0.04)	0.98 (0.03)
79	218[M+](25) 145(100) 147(87)	1594	-	-	-	-	-
80	bisabola-2,10-diene[1,9]oxide	1596	31.12 (0.06)	27.32 (0.09)	36.14 (0.10)	28.54 (0.09)	38.79 (0.09)
81	218[M+](1) 94(100) 79(52)	1605	-	-	-	-	-
82	218[M+](26) 145(100) 43(92)	1613	-	-	-	-	-
83	220[M+](2) 94(100) 79(39)	1625	2.24 (0.05)	2.34 (0.04)	1.04 (0.04)	2.11 (0.03)	2.47 (0.03)
84	218[M+](5) 145(100) 160(48)	1641	-	-	-	-	-
85	218[M+](18) 105(100) 120(83)	1646	0.02 (0.01)	0.02 (0.01)	0.11 (0.02)	0.07 (0.02)	0.06 (0.02)
86	218[M+](5) 135(100) 107(52)	1651	-	-	-	-	-
87	220[M+](2) 91(100) 43(91)	1658	-	-	-	-	-
88	220[M+](8) 159(100) 91(81)	1668	0.18 (0.02)	0.07 (0.01)	0.31 (0.02)	0.28 (0.03)	0.18 (0.03)
89	220[M+](5) 161(100) 91(69)	1670	-	-	-	-	-
90	218[M+](38) 145(100) 91(51)	1677	-	-	-	-	-
91	218[M+](2) 179(100) 161(92)	1686	-	-	-	-	-
92	218[M+](9) 105(100) 119(59)	1689	0.11 (0.02)	0.08 (0.02)	0.21 (0.03)	0.18 (0.02)	0.18 (0.02)
93	218[M+](22) 83(100) 94(92)	1699	0.04 (0.00)	0.04 (0.00)	0.18 (0.03)	0.20 (0.02)	0.15 (0.02)
94	218[M+](28) 135(100) 91(77)	1701	-	-	0.06 (0.01)	0.05 (0.01)	0.06 (0.01)
95	218[M+](20) 91(100) 133(98)	1706	0.09 (0.02)	0.11 (0.01)	0.06 (0.01)	0.09 (0.01)	0.05 (0.01)
96	220[M+](4) 110(100) 95(62)	1708	-	-	0.12 (0.02)	0.11 (0.02)	0.13 (0.02)
97	218[M+](11) 123(100) 95(62)	1712	0.14 (0.02)	0.14 (0.01)	-	-	-
98	220[M+](18) 83(100) 125(79)	1722	0.05 (0.01)	0.03 (0.01)	0.12 (0.02)	0.09 (0.03)	0.21 (0.03)
99	218[M+](2) 120(100) 83(34)	1729	0.02 (0.01)	0.02 (0.01)	0.04 (0.02)	0.03 (0.02)	0.04 (0.02)
100	218[M+](1) 121(100) 165(59)	1739	0.02 (0.01)	0.07 (0.01)	0.05 (0.02)	0.04 (0.02)	0.06 (0.02)
101	218[M+](2) 82(100) 41(50)	1744	0.01 (0.02)	0.05 (0.02)	0.10 (0.02)	0.06 (0.02)	0.07 (0.02)
102	218[M+](3) 82(100) 41(49)	1754	0.06 (0.02)	0.09 (0.02)	0.09 (0.02)	0.07 (0.02)	0.12 (0.02)
103	220[M+](9) 137(100) 135(78)	1759	0.05 (0.01)	0.03 (0.01)	0.16 (0.02)	0.11 (0.03)	0.09 (0.03)
104	218[M+](29) 136(100) 121(81)	1762	0.06 (0.02)	0.10 (0.02)	0.08 (0.01)	0.05 (0.01)	0.06 (0.01)
105	218[M+](1) 183(100) 198(61)	1795	0.07 (0.02)	0.07 (0.02)	0.05 (0.01)	0.09 (0.02)	0.07 (0.01)
106	218[M+](17) 82(100) 109(83)	1800	0.04 (0.01)	0.09 (0.02)	0.12 (0.02)	0.09 (0.01)	0.14 (0.02)
107	221[M+](1) 82(100) 67(39)	1808	-	-	0.12 (0.01)	0.08 (0.01)	0.13 (0.01)
	Total		98.09 (1.51)	95.94 (1.46)	97.81 (2.00)	97.20 (1.87)	97.29 (1.79)
	% Identified		65.01 (0.62)	57.69 (0.64)	67.74 (0.88)	63.89 (0.83)	67.27 (0.81)
	Including:						
	Aliphatics		0.20 (0.05)	0.12 (0.03)	0.50 (0.11)	0.47 (0.10)	0.37 (0.09)

Aromatics	0.94 (0.08)	1.44 (0.12)	0.91 (0.11)	1.21 (0.12)	1.36 (0.12)
Monoterpene hydrocarbons	0.04 (0.01)	0.04 (0.01)	0.03 (0.02)	0.04 (0.02)	0.03 (0.01)
Monoterpenoide hydrocarbons	0.08 (0.01)	0.06 (0.01)	-	-	-
Sesquiterpene hydrocarbons	32.62 (0.40)	28.67 (0.37)	29.63 (0.51)	32.99 (0.47)	26.01 (0.47)
Sesquiterpenoide hydrocarbons	31.13 (0.07)	27.36 (0.10)	36.67 (0.13)	29.18 (0.12)	39.50 (0.12)

- less than 0.01%. [a] Retention index on Quadrex 007-5MS column. [b] For abbreviations of samples see Table S1. ( ) standard deviation.

Table S2c. Volatile compounds detected in the samples CSU1-27 – CSU1-31.

No.	Compounds	RI [a]	Code [b]				
			CSU1-27	CSU1-28	CSU1-29	CSU1-30	CSU1-31
1	hexanal	782	0.01 (0.01)	0.03 (0.01)	0.02 (0.01)	0.04 (0.01)	0.01 (0.01)
2	3-methylbutanoic acid	817	0.05 (0.00)	0.03 (0.00)	0.01 (0.00)	0.04 (0.00)	0.02 (0.00)
3	2-methylbutanoic acid	832	-	-	-	-	-
4	3-hexen-1-ol	858	-	-	-	-	-
5	1-hexanol	867	0.07 (0.03)	0.18 (0.03)	0.08 (0.03)	0.09 (0.03)	0.06 (0.03)
6	tricyclene	927	-	-	-	-	-
7	α-pinene	936	0.02 (0.00)	0.01 (0.00)	0.02 (0.00)	0.01 (0.00)	0.01 (0.00)
8	86[M+](50) 42(100) 86(38)	957	0.03 (0.00)	0.03 (0.00)	0.03 (0.00)	0.01 (0.00)	0.04 (0.00)
9	hexanoic acid	975	0.08 (0.01)	0.10 (0.01)	0.13 (0.01)	0.09 (0.01)	0.15 (0.01)
10	β-pinene	975	0.02 (0.01)	0.05 (0.01)	0.03 (0.01)	0.02 (0.01)	0.05 (0.01)
11	1-octen-3-ol	979	0.09 (0.02)	0.07 (0.02)	0.06 (0.02)	0.05 (0.02)	0.08 (0.02)
12	3-octanone	985	0.09 (0.01)	0.80 (0.01)	0.11 (0.01)	0.13 (0.01)	0.08 (0.01)
13	3-octanol	994	-	0.02 (0.00)	0.01 (0.00)	0.03 (0.00)	0.02 (0.00)
14	benzenemethanol	1033	0.12 (0.01)	0.08 (0.01)	0.06 (0.01)	0.07 (0.01)	0.09 (0.01)
15	benzeneacetaldehyde	1043	0.18 (0.03)	0.24 (0.03)	0.17 (0.03)	0.19 (0.03)	0.13 (0.03)
16	2-ethylhexanoic acid	1108	0.07 (0.01)	0.12 (0.01)	0.15 (0.01)	0.14 (0.01)	0.09 (0.01)
17	benzeneethanol	1116	0.49 (0.04)	1.01 (0.04)	0.64 (0.04)	0.61 (0.04)	1.43 (0.04)
18	126[M+](11) 55(100) 98(84)	1154	0.06 (0.01)	0.11 (0.01)	0.07 (0.01)	0.08 (0.01)	0.07 (0.01)
19	122[M+](20) 91(100) 44(58)	1164	-	-	-	-	-
20	140[M+](4) 43(100) 57(60)	1200	0.03 (0.00)	0.01 (0.00)	0.02 (0.00)	0.01 (0.00)	0.05 (0.00)
21	128[M+](5) 44(100) 57(63)	1203	0.06 (0.00)	0.05 (0.00)	0.13 (0.00)	0.04 (0.00)	0.06 (0.00)
22	152[M+](92) 67(100) 109(98)	1217	0.42 (0.06)	0.37 (0.06)	0.35 (0.06)	0.65 (0.06)	0.27 (0.06)
23	phenoxyethanol	1223	0.31 (0.03)	0.34 (0.03)	0.28 (0.03)	0.24 (0.03)	0.39 (0.03)
24	1-phenoxy-2-propanol	1247	0.07 (0.01)	0.05 (0.01)	0.04 (0.01)	0.05 (0.01)	0.04 (0.01)
25	144[M+](38) 44(100) 129(72)	1258	0.03 (0.00)	0.04 (0.00)	0.04 (0.00)	0.03 (0.00)	0.04 (0.00)
26	bornyl acetate	1285	-	-	-	-	-
27	isobornyl acetate	1290	-	-	-	-	-
28	189(8) 121(100) 93(82)	1320	0.04 (0.01)	0.03 (0.01)	0.06 (0.01)	0.05 (0.01)	0.04 (0.01)
29	bicycloelemene	1341	1.45 (0.04)	1.34 (0.04)	1.18 (0.04)	1.37 (0.04)	1.29 (0.04)
30	δ-elemene	1343	-	-	-	-	-
31	204[M+](17) 81(100) 93(83)	1355	-	-	-	-	-
32	anastreptene	1370	7.28 (0.07)	6.14 (0.07)	6.94 (0.07)	8.74 (0.07)	6.43 (0.07)
33	α-funebrene	1385	0.93 (0.03)	0.48 (0.03)	0.72 (0.03)	0.64 (0.03)	0.37 (0.03)
34	β-elemene	1394	0.25 (0.01)	0.10 (0.01)	0.17 (0.01)	0.25 (0.01)	0.16 (0.01)
35	7-epi-sesquithujene	1408	0.16 (0.02)	0.12 (0.02)	0.17 (0.02)	0.18 (0.02)	0.21 (0.02)
36	italicene	1409	0.09 (0.01)	0.18 (0.01)	0.12 (0.01)	0.11 (0.01)	0.15 (0.01)
37	9-aristolene	1423	0.09 (0.01)	0.06 (0.01)	0.05 (0.01)	0.11 (0.01)	0.07 (0.01)
38	1(10),8-aristoladiene	1429	6.23 (0.04)	3.48 (0.04)	5.48 (0.04)	6.92 (0.04)	3.48 (0.04)
39	204[M+](6) 107(100) 79(48)	1432	0.09 (0.02)	0.03 (0.02)	0.08 (0.02)	0.06 (0.02)	0.07 (0.02)
40	202[M+](4) 91(100) 185(89)	1434	-	-	-	-	-
41	204[M+](24) 91(100) 105(92)	1436	0.21 (0.02)	0.12 (0.02)	0.18 (0.02)	0.22 (0.02)	0.16 (0.02)
42	204[M+](18) 107(100) 161(88)	1438	0.23 (0.02)	0.17 (0.02)	0.19 (0.02)	0.23 (0.02)	0.18 (0.02)
43	204[M+](9) 119(100) 91(64)	1439	-	-	-	-	-
44	202[M+](30) 131(100) 159(62)	1440	7.43 (0.04)	6.09 (0.04)	6.43 (0.04)	6.34 (0.04)	8.41 (0.04)
45	202[M+](24) 69(100) 41(87)	1443	0.92 (0.03)	0.32 (0.03)	0.68 (0.03)	0.72 (0.03)	0.27 (0.03)
46	β-barbatene	1445	1.24 (0.03)	0.15 (0.03)	1.05 (0.03)	0.48 (0.03)	0.58 (0.03)
47	202[M+](19) 91(100) 41(85)	1450	0.41 (0.02)	0.11 (0.02)	0.32 (0.02)	0.34 (0.02)	0.27 (0.02)
48	202[M+](23) 91(100) 159(93)	1452	-	-	-	-	-
49	202[M+](23) 159(100) 131(74)	1455	2.67 (0.04)	1.25 (0.04)	2.46 (0.04)	1.79 (0.04)	2.53 (0.04)
50	204[M+](7) 159(100) 91(97)	1457	0.47 (0.02)	0.23 (0.02)	0.32 (0.02)	0.42 (0.02)	0.23 (0.02)
51	218[M+](26) 148(100) 133(75)	1466	-	-	-	-	-
52	204[M+](36) 119(100)93(61)	1469	5.01 (0.08)	5.43 (0.08)	7.28 (0.08)	6.67 (0.08)	5.03 (0.08)
53	γ-curcumene	1475	4.71 (0.05)	7.64 (0.05)	7.02 (0.05)	6.48 (0.05)	7.39 (0.05)
54	218[M+](12) 105(100) 91(92)	1476	-	-	-	-	-

55	α-curcumene	1477	2.36 (0.03)	2.57 (0.03)	2.47 (0.03)	1.93 (0.03)	1.78 (0.03)
56	218[M+](25) 105(100) 91(95)	1477	-	-	-	-	-
57	α-zingiberene	1479	0.75 (0.02)	0.38 (0.02)	0.61 (0.02)	0.77 (0.02)	0.24 (0.02)
58	bicyclogermacrene	1481	4.38 (0.04)	2.53 (0.04)	3.42 (0.04)	3.45 (0.04)	2.87 (0.04)
59	202[M+](29) 91(100) 133(92)	1500	5.27 (0.05)	1.98 (0.05)	2.78 (0.05)	3.39 (0.05)	0.78 (0.05)
60	γ-bisabolene	1505	1.27 (0.03)	0.54 (0.03)	0.63 (0.03)	0.87 (0.03)	0.76 (0.03)
61	202[M+](32) 133(100) 105(69)	1510	2.21 (0.02)	1.90 (0.02)	2.32 (0.02)	1.53 (0.02)	1.04 (0.02)
62	218[M+](29) 91(100) 93(97)	1513	0.07 (0.01)	0.50 (0.01)	0.03 (0.01)	0.07 (0.01)	0.04 (0.01)
63	218[M+](11) 132(100) 105(93)	1519	0.27 (0.03)	0.15 (0.03)	0.31 (0.03)	0.42 (0.03)	0.32 (0.03)
64	β-sesquiphellandrene	1524	1.52 (0.04)	1.18 (0.04)	1.48 (0.04)	1.18 (0.04)	1.83 (0.04)
65	218[M+](3) 159(100) 131(76)	1529	1.18 (0.02)	1.27 (0.02)	1.14 (0.02)	1.29 (0.02)	2.10 (0.02)
66	218[M+](24) 148(100) 133(63)	1532	-	-	-	-	-
67	220[M+](8) 85(100) 135(89)	1545	0.28 (0.03)	0.26 (0.03)	0.31 (0.03)	0.18 (0.03)	0.35 (0.03)
68	218[M+](4) 135(100) 107(42)	1548	-	-	-	-	-
69	202[M+](85) 131(100) 91(81)	1551	0.18 (0.02)	0.17 (0.02)	0.31 (0.02)	0.28 (0.02)	0.17 (0.02)
70	218[M+](6) 91(100) 157(90)	1554	-	-	-	-	-
71	218[M+](25) 145(100) 147(97)	1561	-	-	-	-	-
72	218[M+](4) 93(100) 43(75)	1568	-	-	-	-	-
73	4,5-dehydroviridiflorol	1572	0.42 (0.03)	0.48 (0.03)	0.36 (0.03)	0.51 (0.03)	0.88 (0.03)
74	222[M+](3) 43(100) 81(53)	1578	0.12 (0.02)	0.07 (0.02)	0.11 (0.02)	0.14 (0.02)	0.17 (0.02)
75	218[M+](6) 43(100) 93(57)	1579	0.17 (0.02)	0.14 (0.02)	0.09 (0.02)	0.16 (0.02)	0.18 (0.02)
76	218[M+](7) 43(100) 91(67)	1581	-	-	-	-	-
77	220[M+](14) 79(100) 93(93)	1584	0.17 (0.01)	0.21 (0.01)	0.09 (0.01)	0.13 (0.01)	0.15 (0.01)
78	220[M+](1) 94(100) 79(43)	1589	1.21 (0.03)	1.45 (0.03)	1.53 (0.03)	1.69 (0.03)	2.02 (0.03)
79	218[M+](25) 145(100) 147(87)	1594	-	-	-	-	-
80	bisabola-2,10-diene[1,9]oxide	1596	31.41 (0.09)	39.54 (0.09)	34.54 (0.09)	33.27 (0.09)	37.26 (0.09)
81	218[M+](1) 94(100) 79(52)	1605	-	-	-	-	-
82	218[M+](26) 145(100) 43(92)	1613	-	-	-	-	-
83	220[M+](2) 94(100) 79(39)	1625	1.06 (0.03)	2.89 (0.03)	1.27 (0.03)	1.03 (0.03)	1.87 (0.03)
84	218[M+](5) 145(100) 160(48)	1641	-	-	-	-	-
85	218[M+](18) 105(100) 120(83)	1646	0.04 (0.02)	0.08 (0.02)	0.07 (0.02)	0.10 (0.02)	0.14 (0.02)
86	218[M+](5) 135(100) 107(52)	1651	-	-	-	-	-
87	220[M+](2) 91(100) 43(91)	1658	-	-	-	-	-
88	220[M+](8) 159(100) 91(81)	1668	0.23 (0.03)	0.17 (0.03)	0.19 (0.03)	0.17 (0.03)	0.20 (0.03)
89	220[M+](5) 161(100) 91(69)	1670	-	-	-	-	-
90	218[M+](38) 145(100) 91(51)	1677	-	-	-	-	-
91	218[M+](2) 179(100) 161(92)	1686	-	-	-	-	-
92	218[M+](9) 105(100) 119(59)	1689	0.23 (0.02)	0.20 (0.02)	0.17 (0.02)	0.18 (0.02)	0.15 (0.02)
93	218[M+](22) 83(100) 94(92)	1699	0.19 (0.02)	0.16 (0.02)	0.18 (0.02)	0.22 (0.02)	0.09 (0.02)
94	218[M+](28) 135(100) 91(77)	1701	0.07 (0.01)	0.03 (0.01)	0.08 (0.01)	0.05 (0.01)	0.03 (0.01)
95	218[M+](20) 91(100) 133(98)	1706	0.07 (0.01)	0.09 (0.01)	0.06 (0.01)	0.03 (0.01)	0.07 (0.01)
96	220[M+](4) 110(100) 95(62)	1708	0.12 (0.02)	0.10 (0.02)	0.15 (0.02)	0.12 (0.02)	0.09 (0.02)
97	218[M+](11) 123(100) 95(62)	1712	-	-	-	-	-
98	220[M+](18) 83(100) 125(79)	1722	0.09 (0.03)	0.15 (0.03)	0.08 (0.03)	0.20 (0.03)	0.12 (0.03)
99	218[M+](2) 120(100) 83(34)	1729	0.09 (0.02)	0.08 (0.02)	0.07 (0.02)	0.06 (0.02)	0.06 (0.02)
100	218[M+](1) 121(100) 165(59)	1739	0.05 (0.02)	0.08 (0.02)	0.05 (0.02)	0.03 (0.02)	0.06 (0.02)
101	218[M+](2) 82(100) 41(50)	1744	0.08 (0.02)	0.10 (0.02)	0.11 (0.02)	0.09 (0.02)	0.07 (0.02)
102	218[M+](3) 82(100) 41(49)	1754	0.13 (0.02)	0.10 (0.02)	0.06 (0.02)	0.09 (0.02)	0.06 (0.02)
103	220[M+](9) 137(100) 135(78)	1759	0.14 (0.03)	0.16 (0.03)	0.09 (0.03)	0.11 (0.03)	0.14 (0.03)
104	218[M+](29) 136(100) 121(81)	1762	0.08 (0.01)	0.09 (0.01)	0.06 (0.01)	0.07 (0.01)	0.11 (0.01)
105	218[M+](1) 183(100) 198(61)	1795	0.08 (0.01)	0.05 (0.01)	0.09 (0.01)	0.04 (0.01)	0.15 (0.01)
106	218[M+](17) 82(100) 109(83)	1800	0.16 (0.02)	0.11 (0.02)	0.09 (0.02)	0.08 (0.02)	0.11 (0.02)
107	221[M+](1) 82(100) 67(39)	1808	0.14 (0.01)	0.07 (0.01)	0.09 (0.01)	0.11 (0.01)	0.16 (0.01)
	Total		98.50 (1.79)	97.24 (1.79)	98.84 (1.79)	98.78 (1.79)	97.12 (1.79)
	% Identified		66.21 (0.81)	70.04 (0.81)	68.22 (0.81)	69.06 (0.81)	68.40 (0.81)
	Including:						
	Aliphatics		0.46 (0.09)	1.35 (0.09)	0.57 (0.09)	0.61 (0.09)	0.51 (0.09)

Aromatics	1.17 (0.12)	1.72 (0.12)	1.19 (0.12)	1.16 (0.12)	2.08 (0.12)
Monoterpene hydrocarbons	0.04 (0.01)	0.06 (0.01)	0.05 (0.01)	0.03 (0.01)	0.06 (0.01)
Monoterpenoide hydrocarbons	-	-	-	-	-
Sesquiterpene hydrocarbons	32.71 (0.47)	26.89 (0.47)	31.51 (0.47)	33.48 (0.47)	27.61 (0.47)
Sesquiterpenoide hydrocarbons	31.83 (0.12)	40.02 (0.12)	34.90 (0.12)	33.78 (0.12)	38.14 (0.12)

- less than 0.01%. [a] Retention index on Quadrex 007-5MS column. [b] For abbreviations of samples see Table S1. ( ) standard deviation.

Table S2d. Volatile compounds detected in the samples CSU1-32 – CSU2-13.

No.	Compounds	RI [a]	Code [b]				
			CSU1-32	CSU2-10	CSU2-11	CSU2-12	CSU2-13
1	hexanal	782	0.01 (0.01)	0.08 (0.01)	0.03 (0.01)	0.02 (0.01)	0.01 (0.01)
2	3-methylbutanoic acid	817	0.03 (0.00)	-	-	-	-
3	2-methylbutanoic acid	832	-	-	-	-	-
4	3-hexen-1-ol	858	-	-	-	-	0.03 (0.01)
5	1-hexanol	867	0.15 (0.03)	0.23 (0.03)	0.11 (0.02)	0.08 (0.01)	-
6	tricyclene	927	-	0.04 (0.01)	-	-	0.09 (0.01)
7	α-pinene	936	0.03 (0.00)	0.07 (0.01)	0.02 (0.01)	0.01 (0.00)	0.01 (0.01)
8	86[M+](50) 42(100) 86(38)	957	0.02 (0.00)	0.05 (0.01)	0.03 (0.01)	0.01 (0.01)	0.02 (0.01)
9	hexanoic acid	975	0.12 (0.01)	0.02 (0.01)	-	-	-
10	β-pinene	975	0.02 (0.01)	0.04 (0.01)	0.03 (0.01)	0.02 (0.01)	0.04 (0.01)
11	1-octen-3-ol	979	0.06 (0.02)	0.39 (0.03)	0.03 (0.01)	0.07 (0.01)	0.01 (0.00)
12	3-octanone	985	0.19 (0.01)	0.12 (0.05)	0.09 (0.01)	0.06 (0.01)	0.09 (0.01)
13	3-octanol	994	-	0.52 (0.04)	0.12 (0.03)	0.14 (0.02)	0.18 (0.02)
14	benzenemethanol	1033	0.11 (0.01)	0.25 (0.03)	0.21 (0.03)	0.17 (0.02)	0.18 (0.02)
15	benzeneacetaldehyde	1043	0.17 (0.03)	0.89 (0.04)	0.34 (0.03)	0.11 (0.02)	0.06 (0.01)
16	2-ethylhexanoic acid	1108	0.13 (0.01)	0.21 (0.02)	0.07 (0.02)	0.06 (0.01)	0.03 (0.01)
17	benzeneethanol	1116	0.48 (0.04)	1.54 (0.05)	0.38 (0.03)	0.45 (0.02)	1.52 (0.04)
18	126[M+](11) 55(100) 98(84)	1154	0.09 (0.01)	0.07 (0.01)	-	-	-
19	122[M+](20) 91(100) 44(58)	1164	-	0.05 (0.01)	0.03 (0.01)	0.02 (0.01)	0.04 (0.01)
20	140[M+](4) 43(100) 57(60)	1200	0.03 (0.00)	0.18 (0.02)	0.01 (0.01)	0.01 (0.01)	0.02 (0.01)
21	128[M+](5) 44(100) 57(63)	1203	0.04 (0.00)	-	0.01 (0.00)	0.01 (0.00)	0.09 (0.01)
22	152[M+](92) 67(100) 109(98)	1217	0.19 (0.06)	1.29 (0.03)	0.08 (0.01)	0.06 (0.01)	0.09 (0.01)
23	phenoxyethanol	1223	0.43 (0.03)	0.18 (0.02)	0.02 (0.01)	0.04 (0.01)	-
24	1-phenoxy-2-propanol	1247	0.06 (0.01)	0.09 (0.01)	0.12 (0.02)	0.09 (0.01)	0.02 (0.01)
25	144[M+](38) 44(100) 129(72)	1258	0.03 (0.00)	0.16 (0.02)	0.03 (0.01)	0.01 (0.00)	-
26	bornyl acetate	1285	-	0.13 (0.02)	-	-	-
27	isobornyl acetate	1290	-	0.07 (0.01)	-	-	0.05 (0.01)
28	189(8) 121(100) 93(82)	1320	0.03 (0.01)	-	0.06 (0.01)	0.02 (0.01)	0.03 (0.01)
29	bicycloelemene	1341	0.98 (0.04)	0.19 (0.02)	1.02 (0.04)	0.83 (0.03)	0.98 (0.02)
30	δ-elemene	1343	-	0.02 (0.01)	0.14 (0.02)	0.21 (0.02)	0.03 (0.01)
31	204[M+](17) 81(100) 93(83)	1355	-	0.34 (0.03)	0.41 (0.02)	0.28 (0.02)	0.04 (0.01)
32	anastreptene	1370	6.24 (0.07)	6.45 (0.06)	6.11 (0.05)	5.03 (0.08)	3.96 (0.06)
33	α-funebrene	1385	0.48 (0.03)	0.12 (0.02)	0.18 (0.02)	0.29 (0.02)	0.76 (0.04)
34	β-elemene	1394	0.19 (0.01)	0.50 (0.03)	0.69 (0.03)	0.62 (0.03)	0.01 (0.01)
35	7-epi-sesquithujene	1408	0.14 (0.02)	0.12 (0.02)	0.08 (0.02)	0.09 (0.01)	0.12 (0.02)
36	italicene	1409	0.12 (0.01)	0.19 (0.02)	0.16 (0.02)	0.17 (0.02)	0.15 (0.02)
37	9-aristolene	1423	0.08 (0.01)	0.32 (0.03)	0.15 (0.03)	0.11 (0.02)	0.11 (0.02)
38	1(10),8-aristoladiene	1429	4.02 (0.04)	0.11 (0.02)	3.79 (0.11)	2.86 (0.04)	3.87 (0.07)
39	204[M+](6) 107(100) 79(48)	1432	0.05 (0.02)	0.09 (0.02)	0.19 (0.03)	0.14 (0.02)	0.15 (0.02)
40	202[M+](4) 91(100) 185(89)	1434	-	0.11 (0.02)	0.12 (0.02)	0.15 (0.02)	0.07 (0.02)
41	204[M+](24) 91(100) 105(92)	1436	0.14 (0.02)	-	0.14 (0.02)	0.32 (0.03)	0.14 (0.02)
42	204[M+](18) 107(100) 161(88)	1438	0.17 (0.02)	-	0.18 (0.02)	0.35 (0.03)	5.23 (0.06)
43	204[M+](9) 119(100) 91(64)	1439	-	0.08 (0.01)	0.21 (0.03)	0.12 (0.02)	0.13 (0.02)
44	202[M+](30) 131(100) 159(62)	1440	6.23 (0.04)	9.24 (0.10)	6.04 (0.06)	5.64 (0.06)	0.44 (0.04)
45	202[M+](24) 69(100) 41(87)	1443	0.28 (0.03)	0.03 (0.01)	0.04 (0.01)	0.02 (0.01)	0.47 (0.03)
46	β-barbatene	1445	0.42 (0.03)	0.43 (0.03)	0.69 (0.03)	0.86 (0.03)	0.18 (0.02)
47	202[M+](19) 91(100) 41(85)	1450	0.16 (0.02)	2.38 (0.06)	-	-	1.15 (0.07)
48	202[M+](23) 91(100) 159(93)	1452	-	2.03 (0.07)	1.68 (0.03)	1.47 (0.04)	1.47 (0.06)
49	202[M+](23) 159(100) 131(74)	1455	1.78 (0.04)	0.32 (0.04)	1.79 (0.04)	1.64 (0.04)	0.21 (0.03)
50	204[M+](7) 159(100) 91(97)	1457	0.16 (0.02)	0.17 (0.01)	0.21 (0.02)	0.53 (0.03)	0.42 (0.02)
51	218[M+](26) 148(100) 133(75)	1466	-	0.23 (0.02)	0.54 (0.03)	1.03 (0.04)	1.42 (0.04)
52	204[M+](36) 119(100)93(61)	1469	7.13 (0.08)	0.06 (0.01)	-	-	-
53	γ-curcumene	1475	7.03 (0.05)	0.19 (0.02)	0.03 (0.01)	0.05 (0.01)	0.07 (0.02)
54	218[M+](12) 105(100) 91(92)	1476	-	1.34 (0.05)	0.52 (0.04)	0.63 (0.02)	0.69 (0.03)

55	α-curcumene	1477	3.27 (0.03)	-	-	-	0.09 (0.01)
56	218[M+](25) 105(100) 91(95)	1477	-	2.69 (0.07)	1.93 (0.06)	2.94 (0.04)	1.53 (0.05)
57	α-zingiberene	1479	0.48 (0.02)	-	-	-	-
58	bicyclogermacrene	1481	3.49 (0.04)	2.22 (0.08)	5.86 (0.07)	5.53 (0.05)	6.11 (0.08)
59	202[M+](29) 91(100) 133(92)	1500	1.97 (0.05)	-	-	-	-
60	γ-bisabolene	1505	1.14 (0.03)	8.74 (0.10)	5.60 (0.06)	5.00 (0.04)	6.72 (0.07)
61	202[M+](32) 133(100) 105(69)	1510	1.78 (0.02)	-	-	-	-
62	218[M+](29) 91(100) 93(97)	1513	0.03 (0.01)	1.47 (0.05)	1.43 (0.04)	2.54 (0.03)	0.97 (0.03)
63	218[M+](11) 132(100) 105(93)	1519	0.23 (0.03)	1.53 (0.04)	1.39 (0.04)	1.47 (0.03)	2.13 (0.04)
64	β-sesquiphellandrene	1524	1.54 (0.04)	0.03 (0.01)	0.38 (0.02)	0.41 (0.02)	0.12 (0.02)
65	218[M+](3) 159(100) 131(76)	1529	1.23 (0.02)	-	-	-	-
66	218[M+](24) 148(100) 133(63)	1532	-	18.21 (0.11)	11.49 (0.15)	11.63 (0.14)	11.03 (0.07)
67	220[M+](8) 85(100) 135(89)	1545	0.37 (0.03)	0.11 (0.01)	0.63 (0.03)	0.78 (0.05)	0.12 (0.02)
68	218[M+](4) 135(100) 107(42)	1548	-	0.11 (0.02)	0.13 (0.02)	0.21 (0.03)	0.23 (0.02)
69	202[M+](85) 131(100) 91(81)	1551	0.21 (0.02)	-	-	-	-
70	218[M+](6) 91(100) 157(90)	1554	-	0.13 (0.02)	2.19 (0.05)	2.54 (0.05)	2.15 (0.03)
71	218[M+](25) 145(100) 147(97)	1561	-	0.18 (0.03)	0.32 (0.03)	0.19 (0.02)	0.21 (0.02)
72	218[M+](4) 93(100) 43(75)	1568	-	0.55 (0.03)	0.81 (0.04)	0.34 (0.02)	0.89 (0.04)
73	4,5-dehydroviridiflorol	1572	0.43 (0.03)	1.01 (0.04)	0.50 (0.03)	1.54 (0.04)	0.19 (0.01)
74	222[M+](3) 43(100) 81(53)	1578	0.22 (0.02)	3.14 (0.06)	0.10 (0.02)	0.11 (0.02)	0.36 (0.02)
75	218[M+](6) 43(100) 93(57)	1579	0.23 (0.02)	0.43 (0.03)	0.48 (0.03)	0.83 (0.02)	1.07 (0.05)
76	218[M+](7) 43(100) 91(67)	1581	-	0.59 (0.03)	1.44 (0.05)	1.62 (0.04)	0.43 (0.02)
77	220[M+](14) 79(100) 93(93)	1584	0.17 (0.01)	6.53 (0.06)	0.12 (0.02)	0.18 (0.02)	-
78	220[M+](1) 94(100) 79(43)	1589	1.43 (0.03)	-	-	-	-
79	218[M+](25) 145(100) 147(87)	1594	-	8.81 (0.11)	15.94 (0.16)	16.51 (0.13)	17.38 (0.13)
80	bisabola-2,10-diene[1,9]oxide	1596	36.43 (0.09)	3.32 (0.04)	5.11 (0.09)	2.27 (0.06)	2.07 (0.06)
81	218[M+](1) 94(100) 79(52)	1605	-	0.09 (0.01)	4.76 (0.08)	5.63 (0.05)	5.76 (0.07)
82	218[M+](26) 145(100) 43(92)	1613	-	3.13 (0.05)	8.78 (0.09)	7.09 (0.06)	1.03 (0.04)
83	220[M+](2) 94(100) 79(39)	1625	2.16 (0.03)	-	-	-	-
84	218[M+](5) 145(100) 160(48)	1641	-	0.17 (0.02)	0.19 (0.03)	0.27 (0.02)	10.45 (0.06)
85	218[M+](18) 105(100) 120(83)	1646	0.09 (0.02)	0.03 (0.01)	0.15 (0.02)	0.09 (0.01)	0.15 (0.02)
86	218[M+](5) 135(100) 107(52)	1651	-	1.11 (0.06)	0.04 (0.01)	0.05 (0.01)	0.06 (0.01)
87	220[M+](2) 91(100) 43(91)	1658	-	0.16 (0.02)	0.11 (0.02)	0.13 (0.02)	0.92 (0.03)
88	220[M+](8) 159(100) 91(81)	1668	0.19 (0.03)	-	-	-	-
89	220[M+](5) 161(100) 91(69)	1670	-	0.42 (0.03)	0.88 (0.03)	1.03 (0.04)	0.38 (0.01)
90	218[M+](38) 145(100) 91(51)	1677	-	0.01 (0.00)	0.24 (0.02)	0.48 (0.03)	0.11 (0.02)
91	218[M+](2) 179(100) 161(92)	1686	-	0.02 (0.01)	0.06 (0.01)	0.04 (0.01)	0.11 (0.01)
92	218[M+](9) 105(100) 119(59)	1689	0.21 (0.02)	0.29 (0.03)	0.11 (0.01)	0.33 (0.02)	0.09 (0.01)
93	218[M+](22) 83(100) 94(92)	1699	0.15 (0.02)	0.08 (0.01)	0.12 (0.02)	0.32 (0.02)	0.19 (0.02)
94	218[M+](28) 135(100) 91(77)	1701	0.04 (0.01)	0.08 (0.01)	0.05 (0.01)	0.04 (0.01)	0.05 (0.01)
95	218[M+](20) 91(100) 133(98)	1706	0.09 (0.01)	0.08 (0.01)	0.06 (0.01)	0.19 (0.01)	0.09 (0.01)
96	220[M+](4) 110(100) 95(62)	1708	0.13 (0.02)	0.09 (0.02)	0.08 (0.01)	0.06 (0.01)	0.09 (0.01)
97	218[M+](11) 123(100) 95(62)	1712	-	0.72 (0.05)	-	-	0.11 (0.01)
98	220[M+](18) 83(100) 125(79)	1722	0.13 (0.03)	-	0.07 (0.01)	0.15 (0.01)	0.01 (0.00)
99	218[M+](2) 120(100) 83(34)	1729	0.07 (0.02)	-	-	-	-
100	218[M+](1) 121(100) 165(59)	1739	0.07 (0.02)	-	-	-	0.07 (0.01)
101	218[M+](2) 82(100) 41(50)	1744	0.10 (0.02)	0.01 (0.00)	-	-	0.05 (0.01)
102	218[M+](3) 82(100) 41(49)	1754	0.09 (0.02)	0.08 (0.01)	0.14 (0.02)	0.11 (0.02)	0.01 (0.01)
103	220[M+](9) 137(100) 135(78)	1759	0.15 (0.03)	-	-	-	-
104	218[M+](29) 136(100) 121(81)	1762	0.12 (0.01)	-	-	-	0.10 (0.02)
105	218[M+](1) 183(100) 198(61)	1795	0.13 (0.01)	0.49 (0.03)	0.14 (0.02)	0.28 (0.01)	0.01 (0.01)
106	218[M+](17) 82(100) 109(83)	1800	0.09 (0.02)	0.01 (0.01)	-	-	-
107	221[M+](1) 82(100) 67(39)	1808	0.09 (0.01)	0.02 (0.01)	-	-	0.08 (0.01)
	Total		96.97 (1.79)	98.62 (2.57)	98.76 (2.48)	97.83 (2.11)	98.60 (2.22)
	% Identified		68.47 (0.81)	28.83 (0.95)	32.06 (0.89)	27.19 (0.68)	27.86 (0.74)
	Including:						
	Aliphatics		0.69 (0.09)	1.57 (0.19)	0.45 (0.10)	0.43 (0.07)	0.35 (0.06)

Aromatics	1.25 (0.12)	2.95 (0.15)	1.07 (0.12)	0.86 (0.08)	1.78 (0.08)
Monoterpene hydrocarbons	0.05 (0.01)	0.15 (0.03)	0.05 (0.02)	0.03 (0.01)	0.14 (0.03)
Monoterpenoide hydrocarbons	-	0.20 (0.03)	-	-	0.05 (0.01)
Sesquiterpene hydrocarbons	29.62 (0.47)	19.63 (0.47)	24.88 (0.53)	22.06 (0.42)	23.28 (0.49)
Sesquiterpenoide hydrocarbons	36.86 (0.12)	4.33 (0.08)	5.61 (0.12)	3.81 (0.10)	2.26 (0.07)

- less than 0.01%. [a] Retention index on Quadrex 007-5MS column. [b] For abbreviations of samples see Table S1, S2. ( ) standard deviation.

Table S2e. Volatile compounds detected in the samples CSU2-14 – CSU2-18.

No.	Compounds	RI [a]	Code [b]				
			CSU2-14	CSU2-15	CSU2-16	CSU2-17	CSU2-18
1	hexanal	782	0.01 (0.01)	0.02 (0.01)	0.01 (0.01)	0.02 (0.01)	0.03 (0.01)
2	3-methylbutanoic acid	817	-	-	-	-	-
3	2-methylbutanoic acid	832	-	-	-	-	-
4	3-hexen-1-ol	858	0.02 (0.01)	0.02 (0.01)	0.01 (0.01)	0.01 (0.01)	0.05 (0.01)
5	1-hexanol	867	-	-	-	-	-
6	tricyclene	927	0.07 (0.01)	0.06 (0.01)	0.08 (0.01)	0.07 (0.01)	0.08 (0.01)
7	α-pinene	936	0.03 (0.01)	0.01 (0.01)	0.02 (0.01)	0.01 (0.01)	0.02 (0.01)
8	86[M+](50) 42(100) 86(38)	957	0.04 (0.01)	0.02 (0.01)	0.03 (0.01)	0.01 (0.01)	0.06 (0.01)
9	hexanoic acid	975	-	-	-	-	-
10	β-pinene	975	0.03 (0.01)	0.05 (0.01)	0.03 (0.01)	0.06 (0.01)	0.07 (0.01)
11	1-octen-3-ol	979	0.02 (0.01)	0.02 (0.01)	0.04 (0.01)	0.03 (0.01)	0.02 (0.01)
12	3-octanone	985	0.11 (0.02)	0.07 (0.01)	0.06 (0.01)	0.09 (0.01)	0.11 (0.02)
13	3-octanol	994	0.12 (0.02)	0.08 (0.01)	0.14 (0.02)	0.15 (0.02)	0.21 (0.02)
14	benzenemethanol	1033	0.27 (0.02)	0.45 (0.02)	0.97 (0.03)	0.23 (0.02)	0.54 (0.03)
15	benzeneacetaldehyde	1043	0.08 (0.01)	0.04 (0.01)	0.08 (0.02)	0.06 (0.01)	0.13 (0.02)
16	2-ethylhexanoic acid	1108	0.03 (0.01)	0.04 (0.01)	0.05 (0.01)	0.07 (0.01)	0.14 (0.02)
17	benzeneethanol	1116	1.96 (0.04)	1.53 (0.03)	1.47 (0.04)	1.23 (0.06)	0.34 (0.03)
18	126[M+](11) 55(100) 98(84)	1154	-	-	-	-	0.06 (0.01)
19	122[M+](20) 91(100) 44(58)	1164	0.02 (0.01)	0.01 (0.01)	0.03 (0.01)	0.04 (0.01)	0.09 (0.01)
20	140[M+](4) 43(100) 57(60)	1200	0.02 (0.01)	0.01 (0.01)	0.01 (0.01)	0.01 (0.01)	0.01 (0.01)
21	128[M+](5) 44(100) 57(63)	1203	0.19 (0.02)	0.15 (0.02)	0.09 (0.01)	0.09 (0.01)	0.33 (0.03)
22	152[M+](92) 67(100) 109(98)	1217	0.12 (0.02)	0.12 (0.01)	0.18 (0.02)	0.29 (0.02)	0.54 (0.04)
23	phenoxyethanol	1223	-	-	-	-	-
24	1-phenoxy-2-propanol	1247	0.01 (0.00)	0.02 (0.01)	0.01 (0.00)	0.02 (0.01)	-
25	144[M+](38) 44(100) 129(72)	1258	-	-	-	-	0.19 (0.02)
26	bornyl acetate	1285	-	-	-	-	0.23 (0.02)
27	isobornyl acetate	1290	0.06 (0.01)	0.07 (0.01)	0.04 (0.01)	0.05 (0.01)	0.07 (0.01)
28	189(8) 121(100) 93(82)	1320	0.11 (0.01)	0.09 (0.01)	0.14 (0.02)	0.16 (0.02)	0.02 (0.01)
29	bicycloelemene	1341	0.84 (0.02)	0.78 (0.02)	0.96 (0.04)	1.13 (0.04)	0.68 (0.04)
30	δ-elemene	1343	0.02 (0.01)	0.04 (0.01)	0.05 (0.01)	0.07 (0.02)	0.04 (0.01)
31	204[M+](17) 81(100) 93(83)	1355	0.14 (0.02)	0.19 (0.02)	0.21 (0.02)	0.09 (0.01)	0.57 (0.03)
32	anastreptene	1370	4.28 (0.05)	4.17 (0.05)	4.53 (0.06)	4.00 (0.06)	4.87 (0.05)
33	α-funebrene	1385	0.79 (0.03)	0.59 (0.03)	0.93 (0.05)	1.03 (0.04)	0.93 (0.03)
34	β-elemene	1394	0.03 (0.01)	0.06 (0.01)	0.04 (0.01)	0.07 (0.01)	0.08 (0.01)
35	7-epi-sesquithujene	1408	0.14 (0.01)	0.09 (0.01)	0.18 (0.02)	0.16 (0.02)	0.11 (0.01)
36	italicene	1409	0.18 (0.02)	0.11 (0.01)	0.17 (0.02)	0.21 (0.02)	0.18 (0.02)
37	9-aristolene	1423	0.07 (0.01)	0.13 (0.01)	0.14 (0.01)	0.16 (0.01)	0.17 (0.02)
38	1(10),8-aristoladiene	1429	3.91 (0.04)	2.86 (0.04)	3.42 (0.05)	4.59 (0.06)	3.09 (0.04)
39	204[M+](6) 107(100) 79(48)	1432	0.14 (0.02)	0.16 (0.02)	0.19 (0.02)	0.21 (0.02)	0.17 (0.02)
40	202[M+](4) 91(100) 185(89)	1434	0.16 (0.02)	0.19 (0.02)	0.23 (0.02)	0.18 (0.02)	0.16 (0.02)
41	204[M+](24) 91(100) 105(92)	1436	0.26 (0.02)	0.38 (0.02)	0.24 (0.02)	0.16 (0.02)	0.14 (0.02)
42	204[M+](18) 107(100) 161(88)	1438	5.03 (0.04)	4.98 (0.06)	5.23 (0.06)	5.62 (0.05)	4.54 (0.06)
43	204[M+](9) 119(100) 91(64)	1439	0.15 (0.02)	0.09 (0.01)	0.16 (0.02)	0.18 (0.02)	0.11 (0.01)
44	202[M+](30) 131(100) 159(62)	1440	0.34 (0.02)	0.73 (0.02)	0.49 (0.03)	0.52 (0.03)	0.78 (0.03)
45	202[M+](24) 69(100) 41(87)	1443	0.52 (0.02)	0.63 (0.02)	0.94 (0.04)	0.86 (0.03)	0.27 (0.02)
46	β-barbatene	1445	0.49 (0.02)	0.23 (0.02)	0.19 (0.02)	0.17 (0.02)	0.11 (0.01)
47	202[M+](19) 91(100) 41(85)	1450	1.11 (0.03)	1.23 (0.04)	1.34 (0.06)	1.67 (0.05)	1.48 (0.05)
48	202[M+](23) 91(100) 159(93)	1452	1.35 (0.03)	1.45 (0.04)	1.34 (0.05)	1.49 (0.04)	1.33 (0.04)
49	202[M+](23) 159(100) 131(74)	1455	0.22 (0.02)	0.38 (0.03)	0.35 (0.02)	0.39 (0.03)	0.09 (0.01)
50	204[M+](7) 159(100) 91(97)	1457	0.65 (0.02)	1.49 (0.03)	1.09 (0.04)	0.76 (0.03)	0.34 (0.02)
51	218[M+](26) 148(100) 133(75)	1466	0.68 (0.03)	0.53 (0.02)	0.40 (0.03)	0.51 (0.04)	0.64 (0.03)
52	204[M+](36) 119(100)93(61)	1469	-	-	-	-	-
53	γ-curcumene	1475	0.06 (0.01)	0.04 (0.01)	0.09 (0.01)	0.07 (0.01)	0.08 (0.01)
54	218[M+](12) 105(100) 91(92)	1476	1.83 (0.03)	2.20 (0.03)	2.08 (0.04)	1.64 (0.05)	1.18 (0.03)

55	α-curcumene	1477	0.12 (0.02)	0.13 (0.01)	0.16 (0.02)	0.12 (0.01)	0.29 (0.02)
56	218[M+](25) 105(100) 91(95)	1477	4.58 (0.05)	4.39 (0.04)	5.67 (0.06)	6.24 (0.05)	4.64 (0.05)
57	α-zingiberene	1479	-	-	-	-	-
58	bicyclogermacrene	1481	1.54 (0.04)	1.34 (0.02)	1.19 (0.05)	1.14 (0.04)	1.87 (0.03)
59	202[M+](29) 91(100) 133(92)	1500	-	-	-	-	-
60	γ-bisabolene	1505	5.54 (0.05)	4.28 (0.05)	4.41 (0.06)	6.64 (0.08)	6.12 (0.04)
61	202[M+](32) 133(100) 105(69)	1510	-	-	-	-	-
62	218[M+](29) 91(100) 93(97)	1513	1.38 (0.02)	1.89 (0.04)	2.01 (0.04)	1.38 (0.03)	0.69 (0.03)
63	218[M+](11) 132(100) 105(93)	1519	2.21 (0.03)	1.83 (0.04)	2.25 (0.05)	2.32 (0.04)	1.43 (0.04)
64	β-sesquiphellandrene	1524	0.13 (0.02)	0.52 (0.03)	0.33 (0.03)	0.21 (0.02)	0.09 (0.01)
65	218[M+](3) 159(100) 131(76)	1529	-	-	-	-	-
66	218[M+](24) 148(100) 133(63)	1532	10.78 (0.06)	11.24 (0.11)	10.48 (0.11)	10.16 (0.12)	10.57 (0.12)
67	220[M+](8) 85(100) 135(89)	1545	0.16 (0.02)	0.17 (0.02)	0.09 (0.01)	0.12 (0.01)	0.03 (0.01)
68	218[M+](4) 135(100) 107(42)	1548	0.28 (0.02)	0.34 (0.02)	0.33 (0.02)	0.28 (0.02)	0.10 (0.01)
69	202[M+](85) 131(100) 91(81)	1551	-	-	-	-	-
70	218[M+](6) 91(100) 157(90)	1554	2.23 (0.06)	2.59 (0.04)	2.41 (0.04)	2.26 (0.05)	2.07 (0.03)
71	218[M+](25) 145(100) 147(97)	1561	0.22 (0.02)	0.29 (0.01)	0.18 (0.02)	0.19 (0.02)	0.16 (0.02)
72	218[M+](4) 93(100) 43(75)	1568	0.96 (0.03)	0.67 (0.03)	0.45 (0.03)	0.32 (0.03)	0.68 (0.03)
73	4,5-dehydroviridiflorol	1572	0.23 (0.02)	1.49 (0.04)	0.97 (0.03)	0.33 (0.02)	0.18 (0.01)
74	222[M+](3) 43(100) 81(53)	1578	0.47 (0.03)	0.43 (0.02)	0.59 (0.03)	0.61 (0.04)	0.82 (0.03)
75	218[M+](6) 43(100) 93(57)	1579	1.23 (0.04)	1.54 (0.04)	1.49 (0.05)	1.22 (0.05)	1.04 (0.04)
76	218[M+](7) 43(100) 91(67)	1581	0.76 (0.03)	0.83 (0.03)	0.56 (0.03)	0.17 (0.02)	0.23 (0.02)
77	220[M+](14) 79(100) 93(93)	1584	-	-	-	-	-
78	220[M+](1) 94(100) 79(43)	1589	-	-	-	-	-
79	218[M+](25) 145(100) 147(87)	1594	16.68 (0.15)	17.23 (0.15)	16.78 (0.17)	15.01 (0.19)	18.10 (0.17)
80	bisabola-2,10-diene[1,9]oxide	1596	3.87 (0.04)	2.12 (0.04)	1.96 (0.03)	3.43 (0.06)	4.52 (0.05)
81	218[M+](1) 94(100) 79(52)	1605	2.96 (0.05)	1.69 (0.04)	2.21 (0.04)	2.32 (0.05)	6.99 (0.06)
82	218[M+](26) 145(100) 43(92)	1613	2.17 (0.04)	2.76 (0.03)	2.14 (0.05)	1.97 (0.05)	1.03 (0.04)
83	220[M+](2) 94(100) 79(39)	1625	-	-	-	-	-
84	218[M+](5) 145(100) 160(48)	1641	9.29 (0.07)	11.12 (0.12)	11.27 (0.12)	11.37 (0.13)	10.03 (0.14)
85	218[M+](18) 105(100) 120(83)	1646	0.19 (0.02)	0.22 (0.01)	0.18 (0.02)	0.17 (0.02)	-
86	218[M+](5) 135(100) 107(52)	1651	0.10 (0.01)	0.12 (0.01)	0.06 (0.01)	0.07 (0.01)	0.04 (0.01)
87	220[M+](2) 91(100) 43(91)	1658	1.33 (0.04)	1.37 (0.03)	0.65 (0.04)	0.62 (0.04)	0.67 (0.02)
88	220[M+](8) 159(100) 91(81)	1668	-	-	-	-	-
89	220[M+](5) 161(100) 91(69)	1670	0.29 (0.02)	0.47 (0.03)	0.38 (0.03)	0.39 (0.03)	0.27 (0.03)
90	218[M+](38) 145(100) 91(51)	1677	0.19 (0.02)	0.15 (0.02)	0.13 (0.02)	0.16 (0.02)	0.11 (0.02)
91	218[M+](2) 179(100) 161(92)	1686	0.13 (0.02)	0.10 (0.02)	0.12 (0.02)	0.11 (0.01)	0.15 (0.02)
92	218[M+](9) 105(100) 119(59)	1689	0.21 (0.02)	0.14 (0.02)	0.13 (0.02)	0.12 (0.02)	-
93	218[M+](22) 83(100) 94(92)	1699	0.09 (0.01)	0.16 (0.02)	0.15 (0.01)	0.21 (0.02)	0.17 (0.02)
94	218[M+](28) 135(100) 91(77)	1701	0.08 (0.01)	0.11 (0.01)	0.08 (0.01)	0.06 (0.01)	0.07 (0.01)
95	218[M+](20) 91(100) 133(98)	1706	0.06 (0.01)	0.07 (0.01)	0.02 (0.01)	0.04 (0.01)	0.08 (0.01)
96	220[M+](4) 110(100) 95(62)	1708	0.08 (0.01)	0.11 (0.01)	0.03 (0.01)	0.06 (0.01)	-
97	218[M+](11) 123(100) 95(62)	1712	0.06 (0.01)	0.04 (0.01)	0.11 (0.02)	0.09 (0.01)	0.05 (0.01)
98	220[M+](18) 83(100) 125(79)	1722	0.02 (0.01)	0.01 (0.00)	0.01 (0.01)	0.01 (0.01)	0.05 (0.01)
99	218[M+](2) 120(100) 83(34)	1729	-	-	-	-	-
100	218[M+](1) 121(100) 165(59)	1739	0.08 (0.01)	0.13 (0.02)	0.06 (0.01)	0.13 (0.02)	0.01 (0.01)
101	218[M+](2) 82(100) 41(50)	1744	0.07 (0.01)	0.03 (0.01)	0.01 (0.01)	0.02 (0.01)	-
102	218[M+](3) 82(100) 41(49)	1754	0.02 (0.01)	0.01 (0.01)	0.02 (0.01)	0.01 (0.01)	0.02 (0.01)
103	220[M+](9) 137(100) 135(78)	1759	-	-	-	-	-
104	218[M+](29) 136(100) 121(81)	1762	0.14 (0.02)	0.13 (0.02)	0.15 (0.02)	0.09 (0.01)	0.10 (0.02)
105	218[M+](1) 183(100) 198(61)	1795	0.03 (0.01)	0.02 (0.01)	0.02 (0.01)	0.02 (0.01)	-
106	218[M+](17) 82(100) 109(83)	1800	-	-	-	-	-
107	221[M+](1) 82(100) 67(39)	1808	0.15 (0.02)	0.07 (0.01)	0.11 (0.02)	0.13 (0.02)	0.03 (0.01)
	Total		97.82 (2.04)	98.96 (2.09)	98.83 (2.45)	98.96 (2.47)	98.98 (2.22)
	% Identified		25.06 (0.61)	21.46 (0.58)	22.73 (0.72)	25.63 (0.75)	25.45 (0.64)
	Including:						
	Aliphatics		0.31 (0.08)	0.25 (0.06)	0.31 (0.07)	0.37 (0.07)	0.56 (0.09)

Aromatics	2.32 (0.07)	2.04 (0.07)	2.53 (0.09)	1.54 (0.10)	1.01 (0.08)
Monoterpene hydrocarbons	0.13 (0.03)	0.12 (0.03)	0.13 (0.03)	0.14 (0.03)	0.17 (0.03)
Monoterpenoide hydrocarbons	0.06 (0.01)	0.07 (0.01)	0.04 (0.01)	0.05 (0.01)	0.30 (0.03)
Sesquiterpene hydrocarbons	18.14 (0.36)	15.37 (0.33)	16.79 (0.46)	19.77 (0.46)	18.71 (0.35)
Sesquiterpenoide hydrocarbons	4.10 (0.06)	3.61 (0.08)	2.93 (0.06)	3.76 (0.08)	4.70 (0.06)

- less than 0.01%. [a] Retention index on Quadrex 007-5MS column. [b] For abbreviations of samples see Table S2. ( ) standard deviation.

Table S3. The *Calypogeia suecica* group 1 sampling data in 2021 year used for studies.

Sample Code	Collection Place	Geographical Coordinates	Date month year
CSU1-1	SE Poland, Bieszczady Mts, Górnej Solinki Valley	49°06'25.1"N 22°32'29.3"E	08.2021
CSU1-2	SE Poland, Bieszczady Mts, Średni Lutowy Stream	49°07'29.5"N 22°28'15.5"E	08.2021
CSU1-3	SE Poland, Bieszczady Mts, Wielka Rawka	49°10'48.2"N 22°58'50.8"E	08.2021
CSU1-4	SE Poland, Bieszczady Mts, Terebowiec Stream	49°10'53.7"N 20°70'27.9"E	08.2021
CSU1-5	S Poland, Małe Pieniny Mts, tributary of Skalski	49°23'39.0"N 20°34'03.0"E	09.2021
CSU1-6	S Poland, Małe Pieniny Mts, Stary Stream	49°24'32.1"N 20°32'29.2"E	05.2021
CSU1-7	S Poland, Pieniny Mts, Łonny Stream	49°26′22.0″N 20°24′44.1″E	08.2021
CSU1-8	S Poland, Tatry Mts, Sucha Woda Valley	49°17'00.7"N 20°02'10.2"E	09.2021
CSU1-9	S Poland, Tatry Mts, Pańszczyca Valley	49°14'26.8"N 20°02'04.5"E	09.2021
CSU1-10	S Poland, Tatry Mts, Wyżni Staw Toporowy	49°16'46.4"N 20°01'47.7"E	08.2021
CSU1-11	S Poland, Tatry Mts, Wielka Sucha Valley	49°16'25.2"N 19°49'47.1"E	09.2021
CSU1-12	S Poland, Tatry Mts, Droga nad Reglami	49°15'45.1"N 19°52'16.4"E	09.2021
CSU1-13	S Poland, Tatry Mts, Suchej Wody Valley	49°27'09.2"N 20°03'55.3"E	09.2021
CSU1-14	S Poland, Tatry Mts, Skupniów Upłaz	49°15'36.6"N 19°59'51.3"E	09.2021
CSU1-15	S Poland, Tatry Mts, Kościeliska Valley	49°15'18.3"N, 19°51'55.4"E	09.2021
CSU1-16	S Poland, Tatry Mts, Kraków Gorge	49°23'86.3"N, 19°86'65.2"E	09.2021

Table S4. The *Calypogeia suecica* group 2 sampling data in 2021 year used for studies.

Sample Code	Collection Place	Geographical Coordinates	Date month year
CSU2-1	SE Poland, Bieszczady Mts, Wielki Lutowy Stream	49°06'44.9"N 22°27'01.8"E	08.2021
CSU2-2	S Poland, Beskid Sądecki Mts, Czarny Stream	49°24'51.6"N 20°55'40.7"E	08.2021
CSU2-3	S Poland, Beskid Sądecki Mts, Czarny Stream	49°24'51.6"N 20°55'40.7"E	08.2021
CSU2-4	S Poland, Pieniny Mts, Zamkowa Mts	49°25'12.6"N 20°25'14.1"E	09.2021
CSU2-5	S Poland, Pieniny Mts, Łonny Stream	49°26′22.0″N 20°24′44.1″E	08.2021
CSU2-6	S Poland, Pieniny Mts, Łonny Stream	49°26′22.0″N 20°24′44.1″E	09.2021
CSU2-7	S Poland, Pieniny Mts, forest Łupiska	49°43′01.8″N 20°43′15.5″E	09.2021
CSU2-8	S Poland, Pieniny Mts, Limbargowy Stream	49°25′09,1″N 20°22′16,0″E	08.2021
CSU2-9	S Poland, Pieniny Mts, Mts, Harczy Grunt Valley	49°43′08,1″N 20°34′11,1″E	08.2021

Table S5. The *Calypogeia suecica* group 1 sampling data in 2022 year used for studies.

Sample Code	Collection Place	Geographical Coordinates	Date month year
CSU1-17	SE Poland, Bieszczady Mts, Górnej Solinki Valley	49°06'25.1"N 22°32'29.3"E	08.2022
CSU1-18	SE Poland, Bieszczady Mts, Średni Lutowy Stream	49°07'29.5"N 22°28'15.5"E	08.2022
CSU1-19	SE Poland, Bieszczady Mts, Wielka Rawka	49°07'29.5"N 22°28'15.5"E	08.2022
CSU1-20	SE Poland, Bieszczady Mts, Terebowiec Stream	49°10'53.7"N 20°70'27.9"E	08.2022
CSU1-21	S Poland, Małe Pieniny Mts, tributary of Skalski	49°23'40.2"N 20°33'52.7"E	09.2022
CSU1-22	S Poland, Małe Pieniny Mts, Stary Stream	49°23'40.2"N 20°33'52.7"E	05.2022
CSU1-23	S Poland, Pieniny Mts, Łonny Stream	49°26′22.0″N 20°24′44.1″E	08.2022
CSU1-24	S Poland, Tatry Mts, Sucha Woda Valley	49°17'00.7"N 20°02'10.2"E	09.2022
CSU1-25	S Poland, Tatry Mts, Pańszczyca Valley	49°14'26.8"N 20°02'04.5"E	09.2022
CSU1-26	S Poland, Tatry Mts, Wyżni Staw Toporowy	49°16'46.4"N 20°01'47.7"E	08.2022
CSU1-27	S Poland, Tatry Mts, Wielka Sucha Valley	49°16'25.2"N 19°49'47.1"E	09.2022
CSU1-28	S Poland, Tatry Mts, Droga nad Reglami	49°15'45.1"N 19°52'16.4"E	09.2022
CSU1-29	S Poland, Tatry Mts, Suchej Wody Valley	49°15'45.1"N 19°52'16.4"E	09.2022
CSU1-30	S Poland, Tatry Mts, Skupniów Upłaz	49°15'36.6"N 19°59'51.3"E	09.2022
CSU1-31	S Poland, Tatry Mts, Kościeliska Valley	49°15'18.3"N, 19°51'55.4"E	09.2022
CSU1-32	S Poland, Tatry Mts, Kraków Gorge	49°15'18.3"N, 19°51'55.4"E	09.2022

Table S6. The *Calypogeia suecica* group 2 sampling data in 2022 year used for studies.

Sample Code	Collection Place	Geographical Coordinates	Date month year
CSU2-10	SE Poland, Bieszczady Mts, Wielki Lutowy Stream	49°06'44.9"N 22°27'01.8"E	08.2022
CSU2-11	S Poland, Beskid Sądecki Mts, Czarny Stream	49°24'51.6"N 20°55'40.7"E	08.2022
CSU2-12	S Poland, Beskid Sądecki Mts, Czarny Stream	49°24'51.6"N 20°55'40.7"E	08.2022
CSU2-13	S Poland, Pieniny Mts, Zamkowa Mts	49°25'12.6"N 20°25'14.1"E	09.2022
CSU2-14	S Poland, Pieniny Mts, Łonny Stream	49°26′22.0″N 20°24′44.1″E	08.2022
CSU2-15	S Poland, Pieniny Mts, Łonny Stream	49°26′22.0″N 20°24′44.1″E	09.2022
CSU2-16	S Poland, Pieniny Mts, forest Łupiska	49°26′22.0″N 20°24′44.1″E	09.2022
CSU2-17	S Poland, Pieniny Mts, Limbargowy Stream	49°25′09,1″N 20°22′16,0″E	08.2022
CSU2-18	S Poland, Pieniny Mts, Mts, Harczy Grunt Valley	49°43′08,1″N 20°34′11,1″E	08.2022

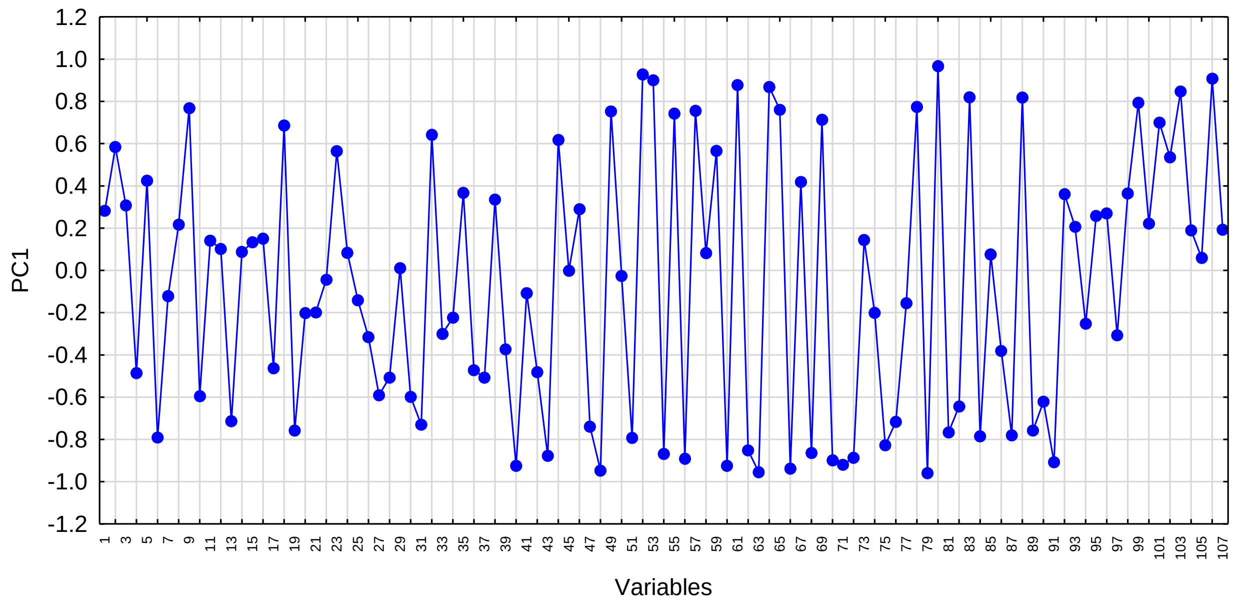

Figure S3. Linear plot of the lodgings for the first principal component PC1.

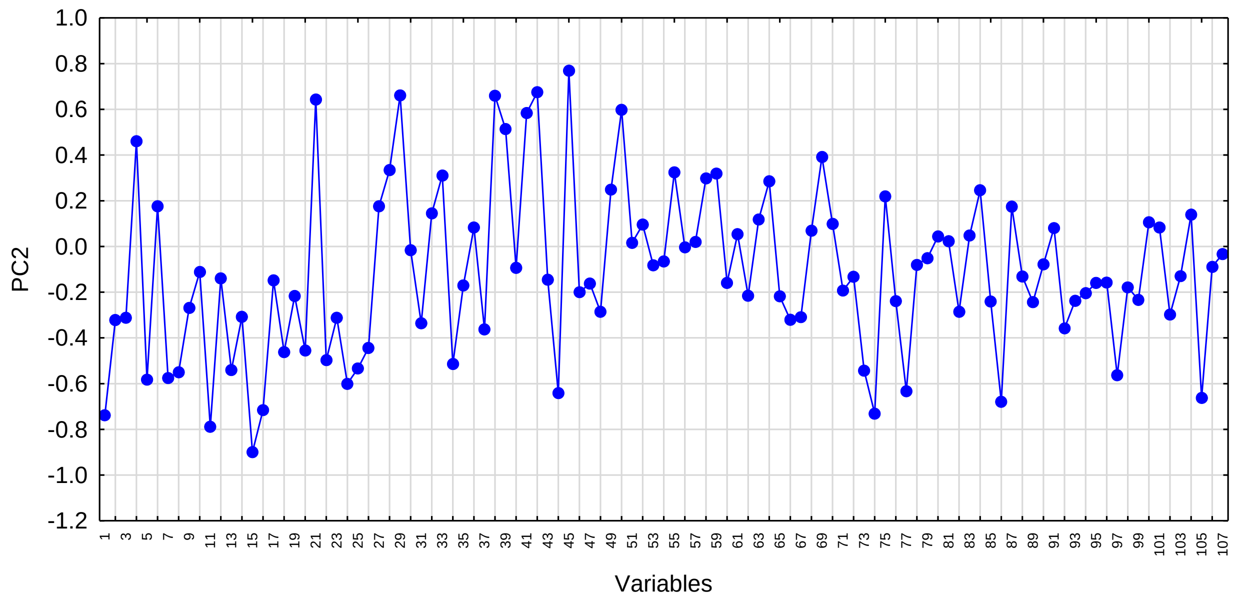

Figure S4. Linear plot of the lodgings for the second principal component PC2.

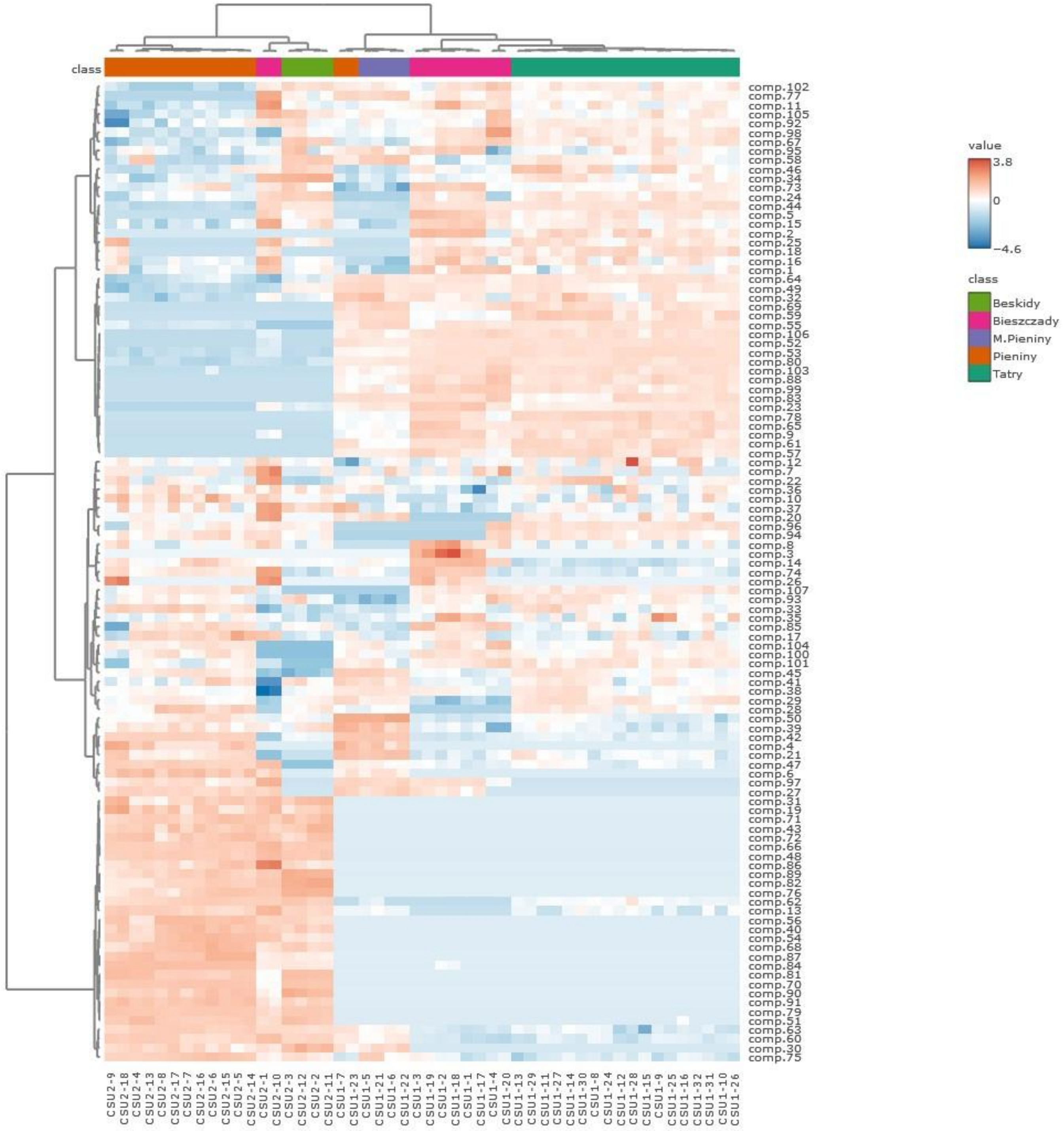

Figure S5. Clustering and heatmap analysis of the 107 chemical compounds detected in the studied *Calypogeia suecica* samples. Annotations bar show clustering of the samples by regions (class). Each cell was colored based on the level of the concentration of the chemical compound in the sample.

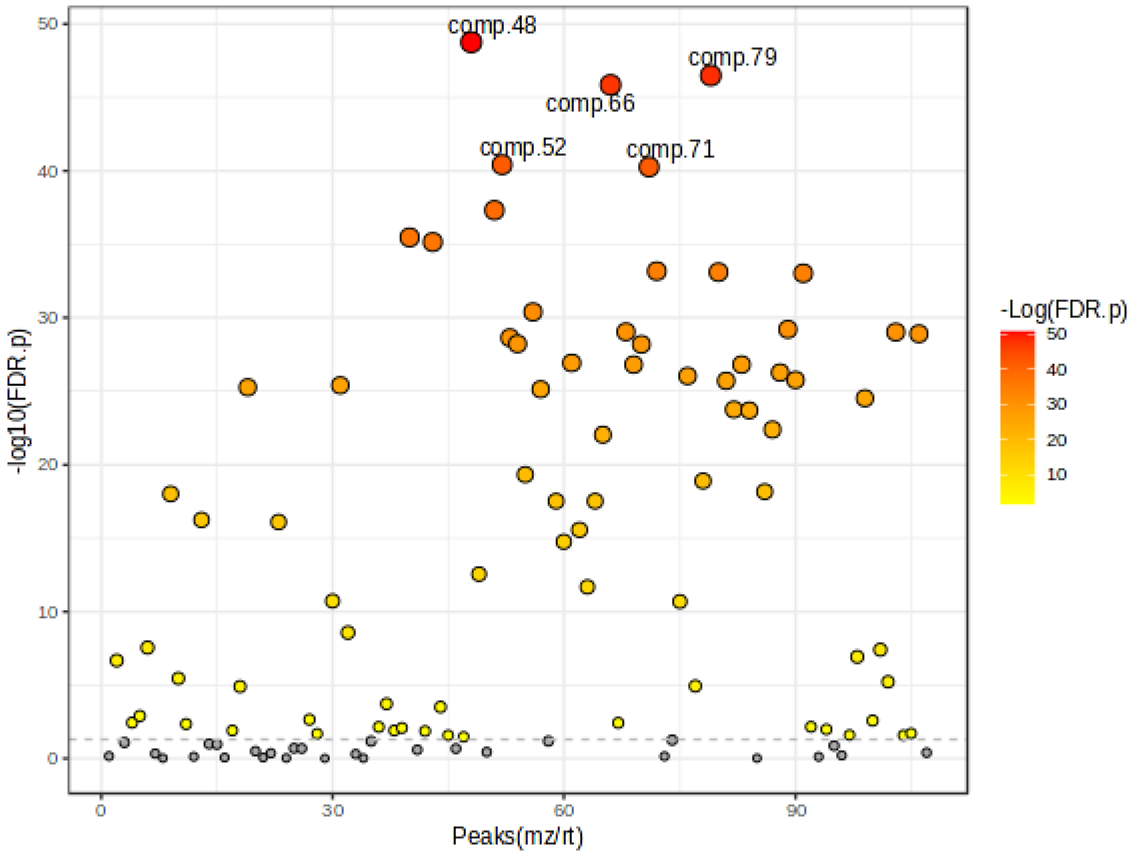

Figure S6. Important features selected by t-tests with threshold 0.1. The red circles represent features above the threshold. Note the p values are transformed by -log10 so that the more signicant features (with smaller p values) will be plotted higher on the graph [MetaboAnalyst 6.0].

Table S7. Top 50 features identified by t-test showing statistically significant differences between genetic groups (group 1 and 2) of *C. suecica*.

	Peaks(mz/rt)	t.stat	p.value	-log10(p)	FDR
1	comp.48	-75.398	1.6206e-51	50.79	1.7341e-49
2	comp.79	-66.582	5.9926e-49	48.222	3.206e-47
3	comp.66	-64.006	3.9015e-48	47.409	1.3915e-46
4	comp.52	48.815	1.4279e-42	41.845	3.8196e-41
5	comp.71	-48.213	2.559e-42	41.592	5.4764e-41
6	comp.51	-41.563	2.6988e-39	38.569	4.8128e-38
7	comp.40	-37.802	2.2427e-37	36.649	3.4281e-36
8	comp.43	-37.109	5.2963e-37	36.276	7.0839e-36
9	comp.72	-33.534	5.7352e-35	34.241	6.8186e-34
10	comp.80	33.345	7.4456e-35	34.128	7.9667e-34
11	comp.91	-33.147	9.7946e-35	34.009	9.5275e-34
12	comp.56	-28.974	4.6171e-32	31.336	4.1169e-31
13	comp.89	-27.234	7.6377e-31	30.117	6.2864e-30
14	comp.68	-26.955	1.2162e-30	29.915	9.2953e-30
15	comp.103	26.891	1.3524e-30	29.869	9.6473e-30
16	comp.106	26.693	1.8874e-30	29.724	1.2622e-29
17	comp.53	26.295	3.7103e-30	29.431	2.3353e-29
18	comp.54	-25.701	1.0335e-29	28.986	6.1436e-29
19	comp.70	-25.628	1.174e-29	28.93	6.6113e-29
20	comp.61	23.977	2.2906e-28	27.64	1.2255e-27
21	comp.69	23.795	3.2106e-28	27.493	1.5836e-27
22	comp.83	23.787	3.2559e-28	27.487	1.5836e-27
23	comp.88	23.102	1.1855e-27	26.926	5.5153e-27
24	comp.76	-22.799	2.122e-27	26.673	9.4607e-27
25	comp.90	-22.455	4.1431e-27	26.383	1.7733e-26
26	comp.81	-22.37	4.8936e-27	26.31	2.0139e-26
27	comp.31	-21.993	1.0307e-26	25.987	4.0845e-26
28	comp.19	-21.816	1.4655e-26	25.834	5.6005e-26
29	comp.57	21.646	2.061e-26	25.686	7.6043e-26
30	comp.99	20.939	8.7499e-26	25.058	3.1208e-25
31	comp.82	-20.085	5.2769e-25	24.278	1.8214e-24
32	comp.84	-20.004	6.276e-25	24.202	2.0985e-24
33	comp.87	-18.644	1.2631e-23	22.899	4.0955e-23
34	comp.65	18.282	2.8873e-23	22.54	9.0865e-23
35	comp.55	15.711	1.4997e-20	19.824	4.5848e-20
36	comp.78	15.313	4.1984e-20	19.377	1.2478e-19
37	comp.86	-14.67	2.3097e-19	18.636	6.6794e-19
38	comp.9	14.53	3.3676e-19	18.473	9.4825e-19
39	comp.64	14.107	1.0694e-18	17.971	2.9341e-18
40	comp.59	14.094	1.1057e-18	17.956	2.9577e-18
41	comp.13	-13.044	2.1421e-17	16.669	5.5902e-17
42	comp.23	12.921	3.0544e-17	16.515	7.7814e-17
43	comp.62	-12.491	1.0752e-16	15.969	2.6755e-16
44	comp.60	-11.861	7.1089e-16	15.148	1.7288e-15
45	comp.49	10.238	1.1653e-13	12.934	2.7708e-13
46	comp.63	-9.6192	8.8894e-13	12.051	2.0678e-12
47	comp.30	-8.9543	8.2785e-12	11.082	1.8847e-11
48	comp.75	-8.9222	9.2332e-12	11.035	2.0582e-11
49	comp.32	7.5093	1.2239e-09	8.9123	2.6726e-09
50	comp.6	-6.8369	1.308e-08	7.8834	2.7991e-08

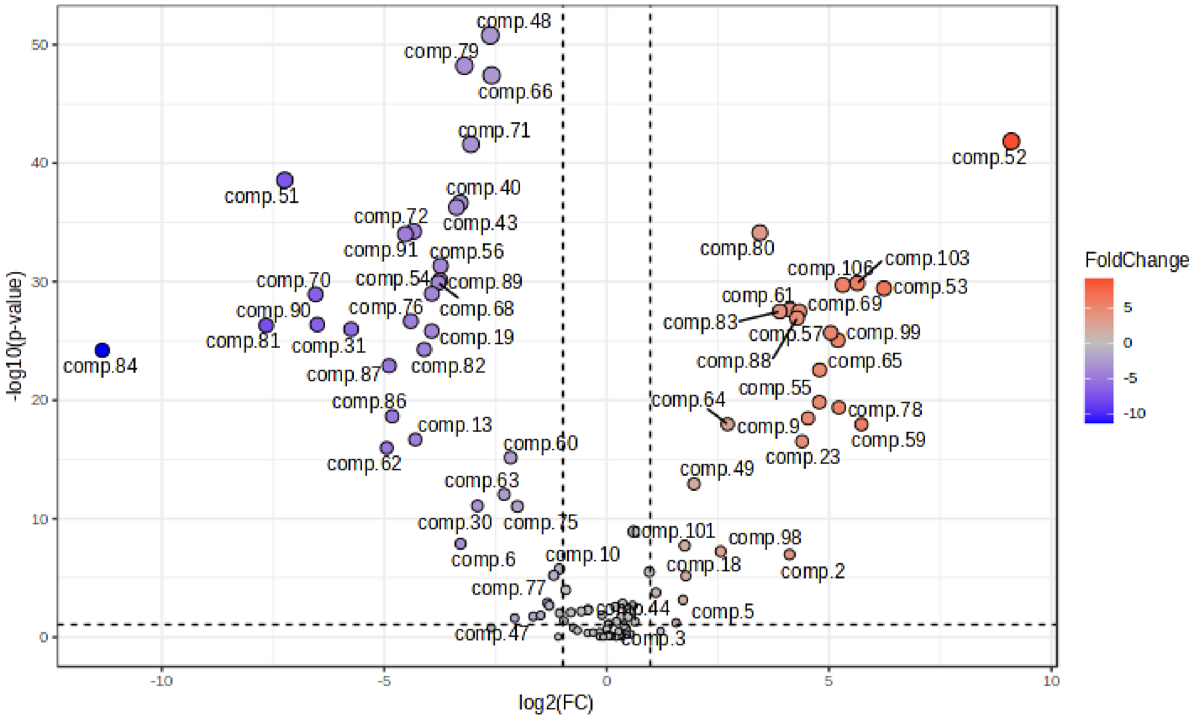

Figure S7. Important features selected by volcano plot with fold change threshold (x) 2 and t-tests threshold (y) 0.1. The red circles represent features above the threshold. Note both fold changes and p values are log transformed. The further its position away from the (0,0), the more signicant the feature is [MetaboAnalyst 6.0].

.

Table S8. Top 50 features identified by volcano plot distinguishing genetic groups (group 1 and 2) of *C. suecica*.

	Peaks(mz/rt)	FC	log2(FC)	raw.pval	-log10(p)
1	comp.48	0.16302	-2.6169	1.6206e-51	50.79
2	comp.79	0.10897	-3.1979	5.9926e-49	48.222
3	comp.66	0.16652	-2.5862	3.9015e-48	47.409
4	comp.52	548.01	9.0981	1.4279e-42	41.845
5	comp.71	0.12062	-3.0515	2.559e-42	41.592
6	comp.51	0.0066285	-7.2371	2.6988e-39	38.569
7	comp.40	0.10202	-3.293	2.2427e-37	36.649
8	comp.43	0.096429	-3.3744	5.2963e-37	36.276
9	comp.72	0.049405	-4.3392	5.7352e-35	34.241
10	comp.80	10.89	3.445	7.4456e-35	34.128
11	comp.91	0.043373	-4.527	9.7946e-35	34.009
12	comp.56	0.075141	-3.7342	4.6171e-32	31.336
13	comp.89	0.07451	-3.7464	7.6377e-31	30.117
14	comp.68	0.072544	-3.785	1.2162e-30	29.915
15	comp.103	49.73	5.636	1.3524e-30	29.869
16	comp.106	39.591	5.3071	1.8874e-30	29.724
17	comp.53	75.29	6.2344	3.7103e-30	29.431
18	comp.54	0.06559	-3.9304	1.0335e-29	28.986
19	comp.70	0.010726	-6.5428	1.174e-29	28.93
20	comp.61	17.417	4.1224	2.2906e-28	27.64
21	comp.69	20.13	4.3313	3.2106e-28	27.493
22	comp.83	14.852	3.8926	3.2559e-28	27.487
23	comp.88	19.427	4.28	1.1855e-27	26.926
24	comp.76	0.047259	-4.4033	2.122e-27	26.673
25	comp.90	0.011009	-6.5051	4.1431e-27	26.383
26	comp.81	0.004964	-7.6543	4.8936e-27	26.31
27	comp.31	0.018653	-5.7445	1.0307e-26	25.987
28	comp.19	0.065455	-3.9334	1.4655e-26	25.834
29	comp.57	32.847	5.0377	2.061e-26	25.686
30	comp.99	36.719	5.1984	8.7499e-26	25.058
31	comp.82	0.058239	-4.1019	5.2769e-25	24.278
32	comp.84	0.00038598	-11.339	6.276e-25	24.202
33	comp.87	0.033702	-4.891	1.2631e-23	22.899
34	comp.65	27.643	4.7889	2.8873e-23	22.54
35	comp.55	27.512	4.782	1.4997e-20	19.824
36	comp.78	37.302	5.2212	4.1984e-20	19.377
37	comp.86	0.03541	-4.8197	2.3097e-19	18.636
38	comp.9	23.044	4.5263	3.3676e-19	18.473
39	comp.64	6.5848	2.7191	1.0694e-18	17.971
40	comp.59	52.985	5.7275	1.1057e-18	17.956
41	comp.13	0.050771	-4.2998	2.1421e-17	16.669
42	comp.23	21.015	4.3934	3.0544e-17	16.515
43	comp.62	0.032515	-4.9428	1.0752e-16	15.969
44	comp.60	0.22392	-2.1589	7.1089e-16	15.148
45	comp.49	3.9025	1.9644	1.1653e-13	12.934
46	comp.63	0.20228	-2.3055	8.8894e-13	12.051
47	comp.30	0.13346	-2.9056	8.2785e-12	11.082
48	comp.75	0.249	-2.0058	9.2332e-12	11.035
49	comp.6	0.10267	-3.284	1.308e-08	7.8834
50	comp.101	3.3721	1.7537	1.8656e-08	7.7292
